# To name but a few: descriptions of five new species of *Terebellides* (Annelida, Trichobranchidae) from the North East Atlantic

**DOI:** 10.3897/zookeys.992.55977

**Published:** 2020-11-12

**Authors:** Julio Parapar, María Capa, Arne Nygren, Juan Moreira

**Affiliations:** 1 Departamento de Bioloxía, Universidade da Coruña, Spain Universidade da Coruña Coruña Spain; 2 Departament de Biologia, Universitat de les Illes Balears, Spain Universitat de les Illes Balears Mallorca Spain; 3 Sjöfartmuseet Akvariet, Göteborg, Sweden and Institutionen för marina vetenskaper, Göteborgs Universitet, Sweden Göteborgs Universitet Göteborg Sweden; 4 Departamento de Biología (Zoología) & Centro de Investigación en Biodiversidad y Cambio Global (CIBC-UAM), Facultad de Ciencias, Universidad Autónoma de Madrid, Spain Universidad Autónoma de Madrid Madrid Spain

**Keywords:** DNA barcoding, DNA species delineation, identification key, integrative taxonomy, new species, North East Atlantic, polychaetes, SEM, systematics

## Abstract

The number of described species of the genus *Terebellides* Sars, 1835 (Annelida, Trichobranchidae) has greatly increased in the last years, particularly in the North East Atlantic. In this context, this paper deals with several putative species recently delineated by molecular means within a well delimited clade of *Terebellides*. Species are characterised here by a combination of morphological characters, and a complementary nucleotide diagnostic approach. Three species were identified as the nominal species *T. stroemii* Sars, 1835, *T. bigeniculatus* Parapar, Moreira & Helgason, 2011 and *T. europaea*Lavesque et al., 2019. Five species are described as new: *T. bakkeni***sp. nov**., *T. kongsrudi***sp. nov**., *T. norvegica***sp. nov**., *T. ronningae***sp. nov**. and *T. scotica***sp. nov**. The distinctive morphological characters refer to the branchial shape, absence or presence of papillae on lamellae of anterior margin of branchial dorsal lobes, absence or presence of ciliated papillae dorsal to thoracic notopodia, geniculate chaetae in one or two chaetigers, and the morphology of thoracic and abdominal uncini teeth. Furthermore, the description of *T. bigeniculatus* is revised and complemented after examination of type specimens. An updated identification key to all species of the genus in NE Atlantic and a proposal of a classification of different types of abdominal uncini to be used in taxonomy are also included.

## Introduction

The species richness in the genus *Terebellides* Sars, 1835 (Annelida, Trichobranchidae) in the North East Atlantic (NEA hereafter) seemed to be well known after several taxonomic studies (Holthe 1986; Jirkov 1989, 2001; Gagaev 2009; Parapar et al. 2011, 2016c; Jirkov and Leontovich 2013; Parapar and Hutchings 2014). Nevertheless, molecular taxonomy approaches performed recently in a comprehensive sample of NEA*Terebellides* have substantially changed the understanding of the species diversity hidden within members of this genus in European waters. Studies by Nygren et al. (2018) and Lavesque et al. (2019) showed a number of genetic lineages, compatible with the species concept – independently evolving entities that are genetically (and phenotypically) distinct (Barraclough 2010). As a result, the total number of species in the NEA has increased dramatically from seven to 32 (Nygren et al. 2018; Lavesque et al. 2019), but some of these still remain unnamed or not formally described.

*Terebellides* is the most species-rich genus of trichobranchids, with 82 nominal species (Parapar et al. 2020; Read and Fauchald 2020) but fairly homogeneous morphologically. It is distinguished from other members in the family by their characteristic branchiae with a single mid-dorsal stalk on segment 3. However, species identification presents some difficulties as there are no clear boundaries between the intraspecific and interspecific variability of some of the morphological attributes considered of high taxonomic relevance. Species diagnostic features mainly rely on details of the branchiae, shape and size of anterior thoracic lateral lobes, and uncinal morphology (Parapar and Hutchings 2014; Parapar et al. 2016a, 2016b). Surprisingly, analyses of DNA sequences showed a large genetic diversity within the group, especially in mitochondrial markers, and while the genetic intraspecific divergence in the universal barcoding marker cytochrome c oxidase subunit I (COI) ranged from 0 to 3.4%, the interspecific distance between species varied from 8.8 to 22.9% (Nygren et al. 2018).

Phylogenetic analyses consistently showed that the NEA*Terebellides* are divided into four major clades, named Groups A–D in Nygren et al. (2018). The aim of the present paper is the systematic revision of members of Group A (according to Nygren et al. 2018), and the morphological characterization of the species assessed after phylogenetic and species delimitation analyses of DNA sequence data (Nygren et al. 2018). Given that there are some species complexes, with scarce morphological differences between the species, if any, a list of apomorphic nucleotides (present in all sequences of a certain species and unique of that species) is also provided as a complementary diagnostic feature (Rach et al. 2008; Wong et al. 2009).

## Materials and methods

This paper is based on the study of 132 specimens identified as belonging to Group A as defined in Nygren et al. (2018) and corresponding to several putative species. This material is deposited in the Zoological Museum Bergen (**ZMBN**, Bergen, Norway), Göteborg Natural History Museum (**GNM**, Goteborg, Sweden), the Norwegian University of Science and Technology, University Museum (**NTNU-VM**, Trondheim, Norway; Bakken et al. 2020) and the Senckenberg Museum Frankfurt (**SMF**, Frankfurt, Germany).

The sampling area covered in this paper is mostly the Norwegian and Swedish continental shelf but also includes some samples from the Irish and Celtic seas, North Sea, Barents Sea, Greenland Sea, South Icelandic coast and the Arctic Ocean (Suppl. material 1: Table S1; Nygren et al. 2018).

Light microscope images were obtained by means of an Olympus SZX12 stereomicroscope equipped with an Olympus C-5050 digital camera. Line drawings were made with an Olympus BX40 stereomicroscope equipped with camera lucida. Specimens for Scanning Electron Microscopy (SEM) were prepared by critical point drying, covered with gold and examined and photographed under a JEOL JSM-6400 electron microscope at the Servizos de Apoio á Investigación (SAI, Universidade da Coruña, Spain).

Methyl green (MG) staining patterns and thoracic uncini morphology were characterised based on the classification proposed by Schüller and Hutchings (2010) and Parapar et al. (2020) respectively; specimens of similar/comparable size were used.

The species dealt within the present study are quite homogenous morphologically. Therefore, common traits shared by all members of Group A are described first in order to avoid repetition of the same characters in each species description.

For each species, the list of the museum registration numbers and collection details (geographic area, locality, coordinates, depth, collecting date and habitat) is provided in Suppl. material 1: Table S1. Unless specified, each registration number holds a single specimen; associated GenBank DNA sequence accession numbers are provided in Suppl. material 2: Table S2.

**Figure 1.**
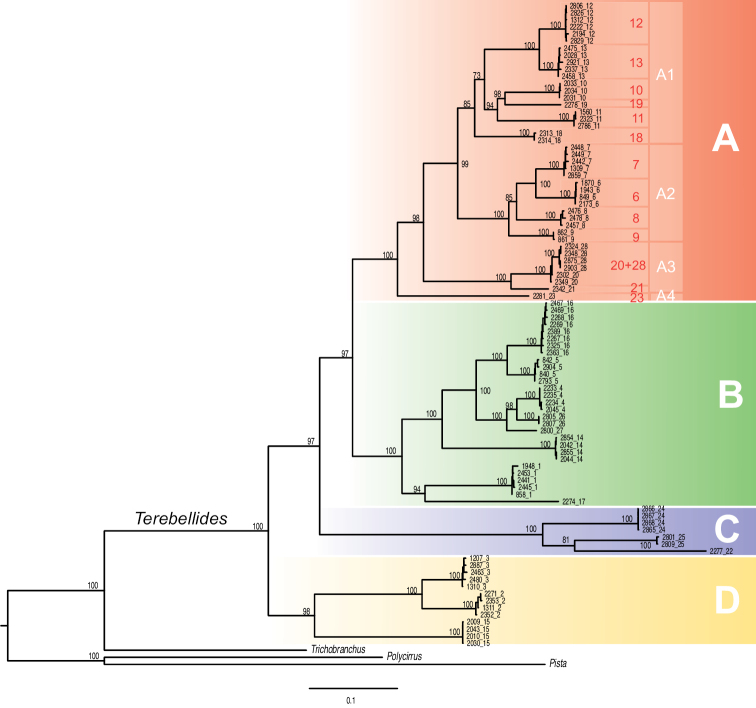
Phylogenetic tree after Maximum Likelihood analyses on a concatenated dataset of cox1, 16S rDNA, ITS2, and 28S rDNA (as in Nygren et al. 2018). Bootstrap support values above nodes. Coloured squares indicate the major clades referred herein as Groups A–D. Within Group A, the focus of present study, subgroups A1–A4 and species 6–13, 18–21, 23, 28 are labelled.

The present systematic account follows the phylogenetic hypothesis presented by Nygren et al. (2018), after phylogenetic analyses of mitochondrial COI (ca. 658bp) and 16S rDNA (ca. 440 bp), and the nuclear ITS2 (290–419 bp) and 28S rDNA (ca. 760 bp) sequences from 513 specimens of *Terebellides* species from the NEA. In their topology, four strongly supported major clades were recovered, and named Groups A–D. We are herein dealing only with members of Group A. Other subgroups (A1–A4) within Group A were established after analyses of combined datasets (Fig. 1; Nygren et al. 2018). In the present study comparison of the morphological traits of species within these subgroups were performed in order to find potential characteristic diagnostic features.

**Figure 2.**
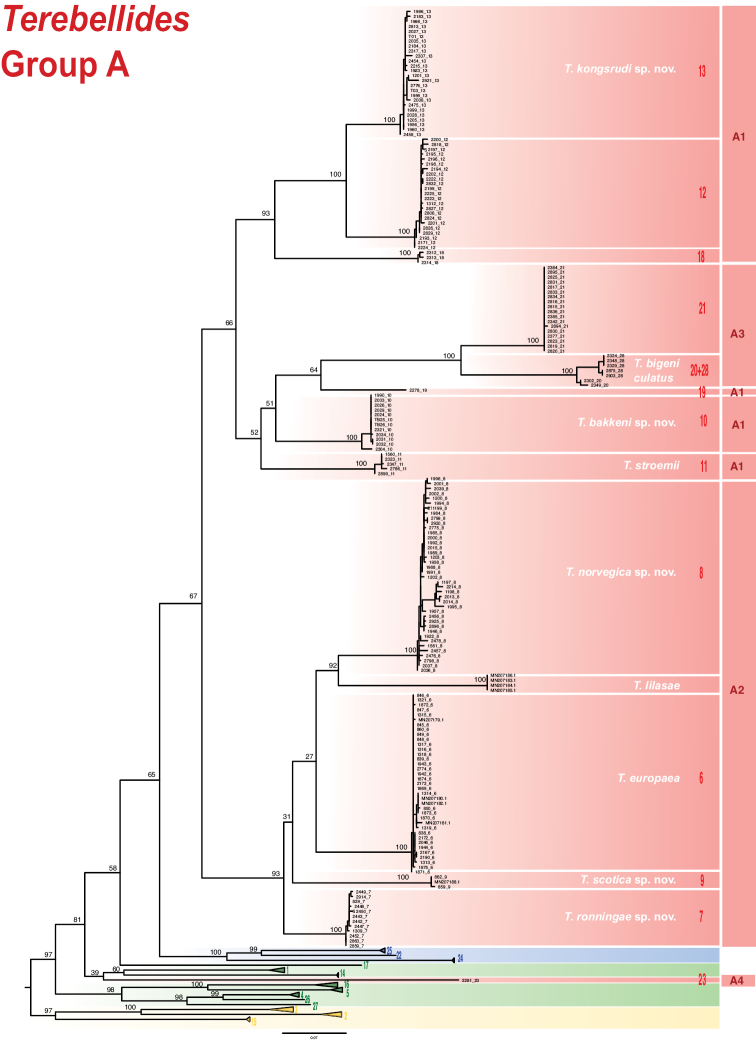
Phylogenetic tree after Maximum Likelihood analyses on a dataset of cox1 (including all sequences in Nygren et al. 2018 and in Lavesque et al. 2019). Bootstrap support values above nodes. Species other than members of Group A are collapsed. Species with names refer to those dealt with in present study.

The COI universal barcoding gene proved to be very informative for species delimitation purposes alone, but insufficient to resolve deeper relationships in the *Terebellides* radiation (Nygren et al. 2018). However, in the present study further analyses based on this mitochondrial marker alone have been performed in order to assess diagnostic nucleotides for each of the species and establish genetic distances between them. Phylogenetic analyses of COI*Terebellides* sequences in GenBank generated by Nygren et al. (2018) and Lavesque et al. (2019) were performed, using *Trichobranchus roseus* (Malm, 1874), *Polycirrus* sp., and *Pista cristata* (Müller, 1776) as outgroups (Nygren et al. 2018). Four hundred and seventy-one sequences were aligned with MAFFT version 7.017 (Katoh et al. 2002), and with default parameters, trimming some starting nucleotides of the sequence of *Terebellides* sp. (MN207188) to become 659 bp alignment. Best-fit model according to Bayesian information criterion – BIC (TVM+F+I+G4), was calculated with IQTREE version 1.6.11 (Nguyen et al. 2015). Maximum likelihood phylogenetic analyses were also run in IQTREE version 1.6.11 (Nguyen et al. 2015), with ultrafast bootstrap (Hoang et al. 2018). Tree topology and support values for the nodes are found in Fig. 2. Given the morphological homogeneity in the *Terebellides* Group A species, GenBank accession numbers (COI sequences) are provided for each species, indicating those belonging to type series. Moreover, unequivocal nucleotide diagnostic characters are provided as the positions in the alignment (nucleotide), with the alignment available in Suppl. material 2: Table S2.

Abbreviations used in text, tables and figures:

**abl** anterior branchial lobe (lobe #5);

**babv** branchial afferent blood vessel;

**bbv** branchial blood vessel;

**bdl** branchial dorsal lobes;

**bdlfl** branchial dorsal lobes fusion line;

**bdltp** branchial dorsal lobe terminal papilla;

**blp** branchial lamellar papillae;

**bst** branchial stem;

**bt** buccal tentacles;

**bvl** branchial ventral lobes;

**bvltp** branchial ventral lobe terminal papilla;

**cap** capitium;

**cbh** contractile branchial heart;

**cr** ciliary row;

**ct** ciliary tuft;

**ctrX** capitium teeth row X;

**dg** digestive gland;

**dpn** dorsal projection of notopodium;

**fi** fore intestine;

**fs** fore stomach;

**gc** geniculate chaetae;

**gr** glandular region;

**hs** hind stomach;

**loli** lower lip;

**MG** Methyl Green;

**nop** notopodial protuberance;

**np** nephridial papilla;

**oes** oesophagus;

**ooc** oocytes;

**ros** rostrum;

**SEM** Scanning Electron Microscope;

**SG** segment;

**STM** stereomicroscope;

**TC** thoracic chaetiger;

**tdp** thoracic dorsal papilla;

**tll** thoracic lateral lappets;

**tm** tentacular membrane;

**TU** thoracic unciniger.

## Systematics

The revision of the specimens of *Terebellides* Group A as found in Nygren et al. (2018) resulted in the identification of three nominal species: *Terebellides stroemii* Sars, 1835, *Terebellides bigeniculatus* Parapar, Moreira & Helgason, 2011 and *T. europaea* Lavesque, Hutchings, Daffe, Nygren & Londoño-Mesa, 2019, and five new species described herein as *T. bakkeni* sp. nov., *T. kongsrudi* sp. nov., *T. norvegica* sp. nov., *T. ronningae* sp. nov. and *T. scotica* sp. nov. The remaining five species will be dealt with in future studies.

Species included in Group A have been grouped as follows: A) subgroup A1 (species 10, 11, 12, 13, 18, 19; as in Nygren et al. 2018), B) subgroup A2 (species 6, 7, 8, 9; as in Nygren et al. 2018), C) subgroup A3 (clades 20 + 28, 21; as in Nygren et al. 2018) and D) subgroup A4 (species 23) (Figs 1, 2, Table 1); material will be described here following this order. Material corresponding to species 12, 18, 19 (A1), 21 (A3) and 23 (A4) is not described/named here. Species 18, 19 and 23 were represented by 1–3 specimens each (see Appendix S36 in Nygren et al. 2018) and are pending formal description until more material is available. Clades 12 and 21 will be described elsewhere by D. Gaeva and I. Jirkov (Shirshov Institute of Oceanology, Russia).

**Table 1.** Comparison of discriminatin g taxonomic characters of the species studied in this work. Cells with text in italic show discriminatory characters of each subgroup. Species 18, 19, and 23 were not studied and 12 and 21 only examined with SEM.

Subgroups		A1						A2				A3		A4
Species sensu Nygren et al. (2018)		10	11	12	13	18	19	6	7	8	9	20 + 28	21	23
SPECIES		*T. bakkeni* sp. nov.	*T. stroemii* Sars, 1835	*Terebellides* sp. 1	*T. kongsrudi* sp. nov.			*T. europaea* Lavesque et al., 2019	*T. ronningae* sp. nov.	*T. norvegica* sp. nov.	*T. scotica* sp. nov.	*T. bigeniculatus* Parapar et al., 2011	*Terebellides* sp. 2	
(as reported/described here)														
Branchiae	type ^(1)^	1	1	1	1	–	–	1	1	1	1	*1 ^(2)^*	*1 ^(2)^*	–
	papillae on lamellae edge	no	no	no	no	–	–	*yes*	*yes*	*yes*	*yes*	no	no	–
Thorax	ciliated papilla dorsal to notopodium	*yes*	*yes*	*yes*	*yes*	–	–	no	no (?)	no	no	*yes*	*yes*	–
	chaetiger(s) with geniculate chaetae	TC6	TC6	TC6	TC6	–	–	TC6	TC6	TC6	TC6	*TC5 + TC6*	*TC5 + TC6*	–
	uncini type ^(3)^	3	3	3	3	–	–	3	1	3	3	3	3	–
Abdomen	uncini type ^(4)^	1A	2	2	1A	–	–	*2*	*2*	*2*	*2*	*1B*	*1B*	–
Bathymetry – Above (A) / Below (B) 200 m depth ^(5)^		A / **B**	A / **B**	A	A / **B**	B	B	A	A	B	A	B	A / **B**	B
Distribution – North (N) /South (S) of 60°N ^(5)^		N	N	S	N / S	N	N	S ^(6)^	N / S	**N** / S	S ^(7)^	N	N	N

^(1)^ sensu Parapar et al. (2016c); ^(2)^ sometimes irregular; ^(3)^ sensu Parapar et al. (2020); ^(4)^ this work; ^(5)^ dominant trend in bold; ^(6)^ Skagerrak and Kattegat; ^(7)^ Irish Sea

### Family Trichobranchidae Malmgren, 1866

#### Genus Terebellides

Taxon classificationAnimaliaTerebellidaTrichobranchidae

Sars, 1835 emended by Schüller & Hutchings, 2013

DFED5DE6-D9D5-56B2-8892-0F7E7BA7DEAD

##### Type species.

*Terebellides stroemii* Sars, 1835, redescribed by Parapar and Hutchings (2014) and neotype deposited.

#### Terebellides

Taxon classificationAnimaliaTerebellidaTrichobranchidae

Group A (sensu Nygren et al. 2018)

C08F6C59-B495-5836-AA9E-9EA4CACE44F1

##### Description.

The morphological features shared by all studied species in Group A are itemized below. Some of these are also shared by Groups B, C and D as defined in Nygren et al. (2018) (see Remarks below).

##### Body appearance.

Complete individuals ranging from 10.0–50.0 mm in length. Body tapering posteriorly with segments increasingly shorter and crowded towards pygidium (Fig. 14A–C). Prostomium compact; large tentacular membrane surrounding mouth (Figs 5C, 14B), with typical buccal tentacles with expanded tips (Figs 15A, 20A). SGI as an expanded structure below tentacular membrane in a lower lip (Figs 14C, 15A, 22A, 24A).

**Figure 3.**
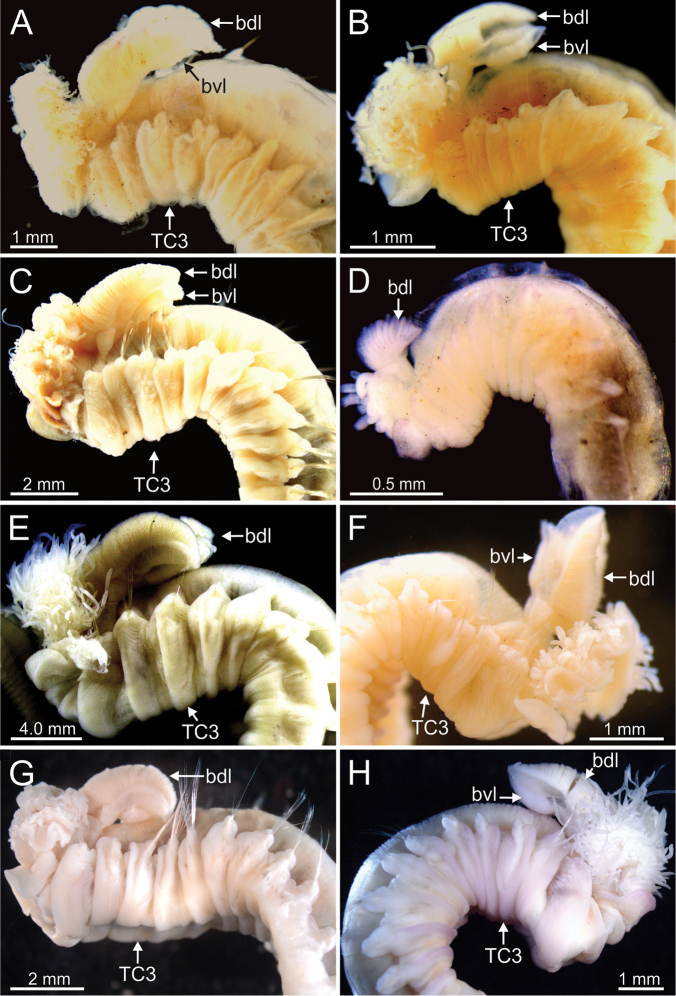
STM photographs of several *Terebellides* species. **A***Terebellides bakkeni* sp. nov. (species 10; holotype, ZMBN116395) **B***Terebellides stroemii* Sars, 1835 (species 11; non-type specimen, ZMBN116397) **C***Terebellides kongsrudi* sp. nov. (species 13; holotype, GNM14632) **D***Terebellides bigeniculatus* Parapar, Moreira & Helgason, 2011 (species 20 + 28; non-type specimen, ZMBN116514) **E***Terebellides europaea* Lavesque, Hutchings, Daffe, Nygren & Londoño-Mesa, 2019 (species 6; non-type specimen, GNM14628) **F***Terebellides ronningae* sp. nov. (species 7; holotype, ZMBN116357) **G***Terebellides norvegica* sp. nov. (species 8; holotype, ZMBN416378) **H***Terebellides scotica* sp. nov. (species 9; holotype, ZMBN116385). Abbreviations: bdl – branchial dorsal lobe; bvl – branchial ventral lobe; TC – thoracic chaetiger.

**Figure 4.**
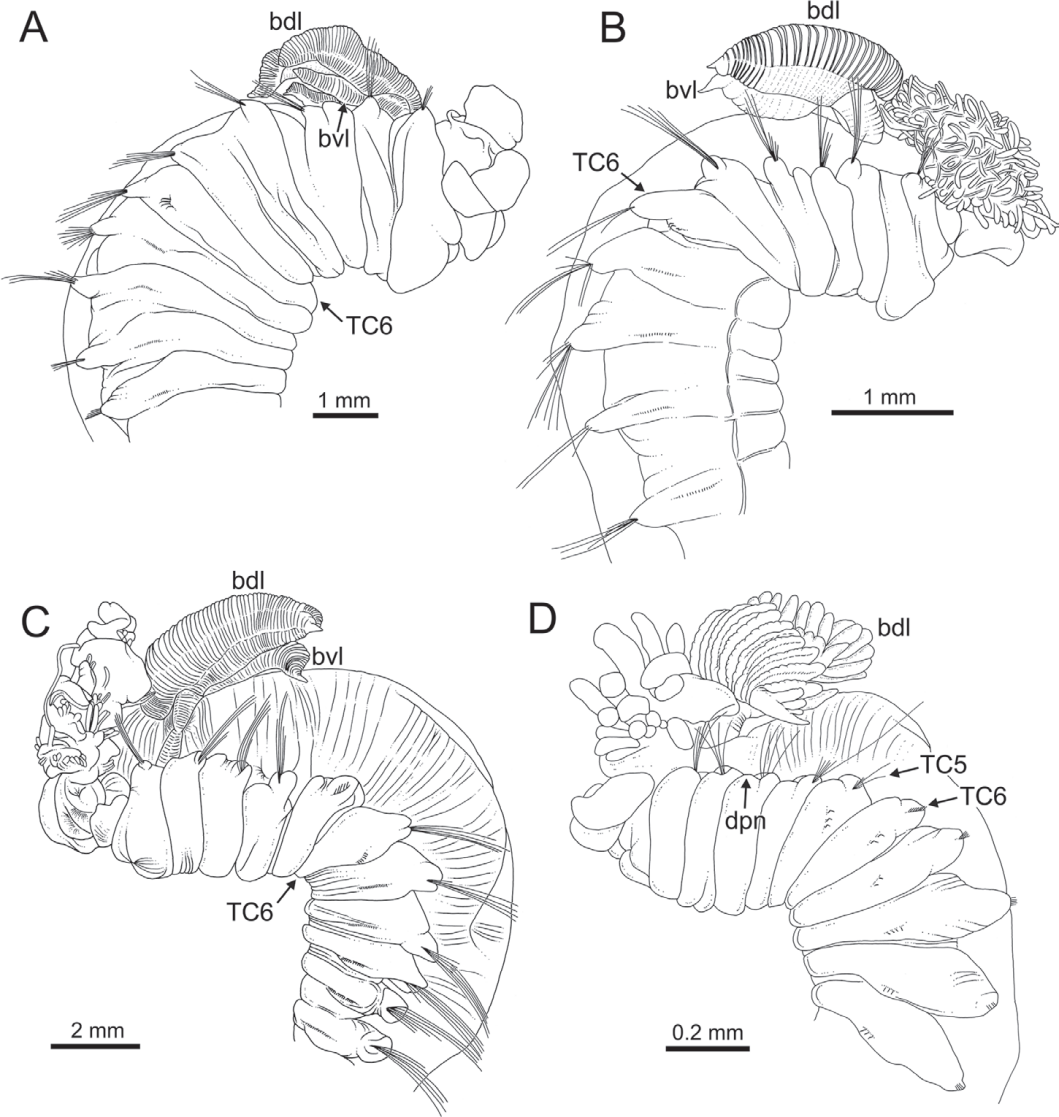
Line drawings of several *Terebellides* species. **A***Terebellides bakkeni* sp. nov. (species 10; holotype, ZMBN116395), anterior end, right lateral view **B***Terebellides stroemii* Sars, 1835 (species 11; non-type specimen, ZMBN116397), anterior end, right lateral view **C***Terebellides kongsrudi* sp. nov. (species 13; holotype, GNM14632), anterior end, left lateral view **D***Terebellides bigeniculatus* Parapar, Moreira & Helgason, 2011 (species 20 + 28; non-type specimen, ZMBN116514), anterior end, left lateral view. Abbreviations: bdl – branchial dorsal lobe; bvl – branchial ventral lobes; dpn – dorsal projection of notopodium ; TC – thoracic chaetiger.

**Figure 5.**
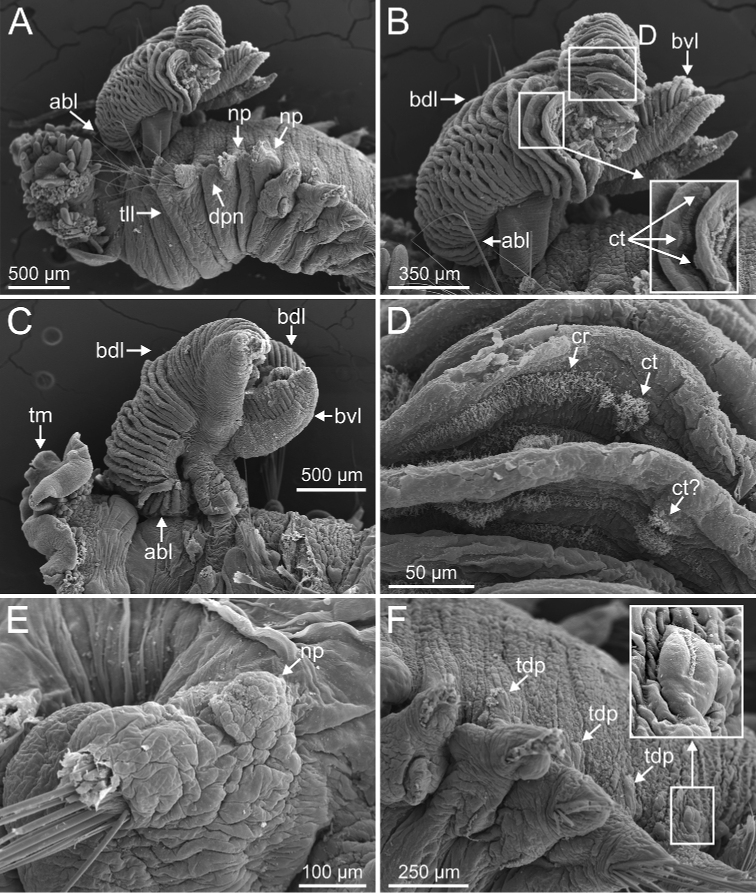
*Terebellides bakkeni* sp. nov. (species 10; paratypes, NTNU-VM-61376 and NTNU-VM-61377), SEM micrographs. **A** anterior end, left lateral view **B, C** branchial lamellae **D** branchial ciliary rows (framed in **B**) **E** nephridial papilla **F** thoracic notopodial papillae (framed: detail of one papilla). Abbreviations: abl – anterior branchial lobe; bdl – branchial dorsal lobe; bvl – branchial ventral lobe; cr – ciliary row; ct – ciliary tuft; dpn – dorsal projection of notopodium; np – nephridial papilla; TC – thoracic chaetiger; tdp – thoracic dorsal papilla; tll – thoracic lateral lobes; tm – tentacular membrane.

***Branchiae***. Branchiae arising as single structure from SGIII, with a single stalked mid-dorsal stem (Figs 5A, 11C, 15A), one pair of dorsal (upper) partially fused lobes (Figs 11B, 15B, 20A), and a pair of shorter ventral (lower) lobes (Fig. 5A, B) obscured or not by dorsal ones (Figs 5A, C, 15A, B). Both dorsal and ventral branchial lobes ending each posteriorly in short terminal papilla (Fig. 20B). Anterior projection of dorsal lobes (fifth lobe) present but short (Fig. 5A, B) and usually obscured by tentacular membrane and buccal tentacles (Fig. 14A, C). Posterior dorsal lobes reaching TC4 (Figs 3, 4, 19). Branchial lamellae provided with several parallel rows of cilia in inner face (Fig. 15C); ciliated papillae not present, ciliary tufts present, sometimes not clearly visible (Fig. 5B, D).

***Thorax***. Eighteen pairs of notopodia (SGIII-SGXX) (Fig. 14B, D), those of TC1 approximately as long as following ones (Figs 20A, 22A) or slightly shorter (Fig. 15A). Lateral lappets and dorsal projections of notopodia in anterior thoracic chaetigers with different degree of development depending on size and preservation conditions, but both more conspicuous on TC2–4/5 (Figs 15A, 22A). All notochaetae as simple capillaries (Figs 11F, 15A). Neuropodia as sessile pinnules from TC5 or TC6 to body end, with uncini in single or double rows, from TC7 throughout. Neuropodia on TC5 or TC5 and TC6, provided with several sharply bent, acute-tipped, geniculate chaetae (Figs 16B, 23A) with minute teeth forming an ill-defined capitium only visible with SEM (Figs 12B, 25B). From TC7, neuropodia with one or several rows of uncini per torus (Figs 16C, 23C), with long shafted denticulate hooks, with large main fang (rostrum) longer than upper crest of teeth (capitium), which is composed by several teeth above main fang of decreasing length (Figs 23D, 25D, E).

**Figure 6.**
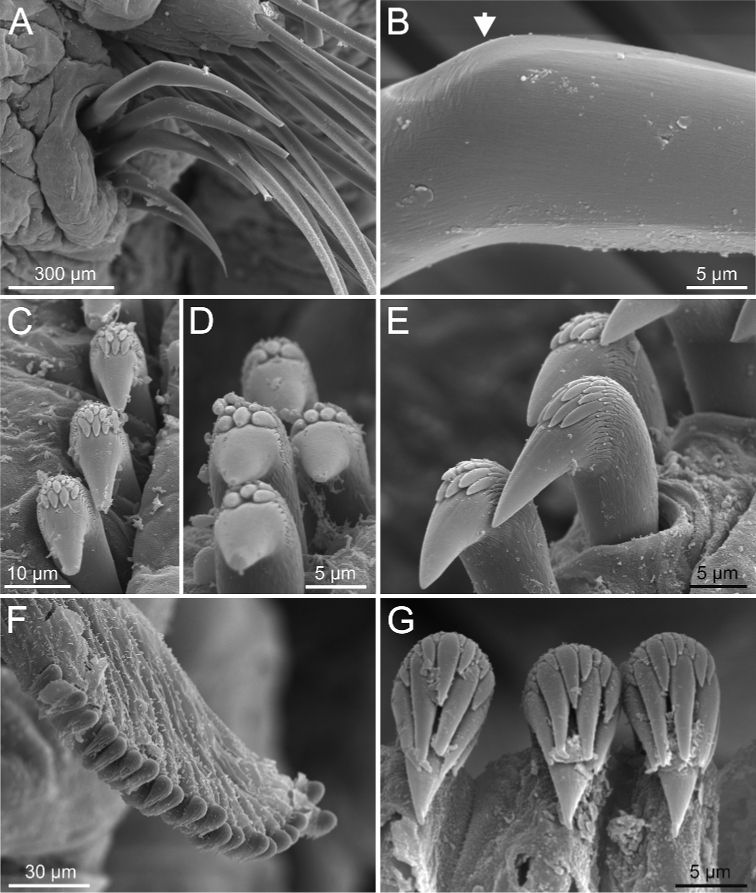
*Terebellides bakkeni* sp. nov. (species 10; paratypes, NTNU-VM-61376 and NTNU-VM-61377), SEM micrographs. **A** TC6 (TU1) geniculate chaetae **B** geniculate chaeta (arrow pointing to capitium) **C–E** thoracic uncini **F** abdominal unciniger **G** detail of three abdominal uncini, frontal view.

***Abdomen and pygidium***. Approximately half as long as thorax and progressively thinner (Fig. 14B). Neuropodia ranging from 18–38 chaetigers and forming erect pinnules (Figs 6F, 12F) with several uncini per torus, number depending of specimen size. Uncini provided with several teeth above rostrum surmounted by a capitium composed of several teeth of decreasing length (Figs 6G, 16E, 21F). Pygidium blunt, as funnel-like depression.

***Colour pattern*.** Colour in preserved specimens pale brown (Fig. 3). MG staining pattern 1 sensu Schüller and Hutchings (2010: 10, fig. 4) and characterised by compact green colouration in CH1–3, then turning into striped pattern in CH4–12 and fading in following segments.

##### Remarks.

Among the aforementioned characters, branchial features might serve to distinguish most of Group A species (except for A3 species) from those in Groups B–D. Those include branchial size, lobes size (i.e., whether dorsal and ventral are of similar size or differ), presence of terminal papilla/filament on posterior lobes, and presence of ciliary structures (rows, tufts or buttons) on lamellae. Other taxa described or reported worldwide bear similar branchiae including *T. stroemii* sensu Parapar et al. (2011) from Iceland and sensu Parapar et al. (2013) from the Adriatic Sea, *T. kerguelensis* McIntosh, 1885 and *T. longicaudatus* Hessle, 1917 from Antarctic latitudes (Parapar and Moreira 2008a, 2008b), and *T. kobei* Hessle, 1917 from Japan (Imajima and Williams 1985).

The other species groups as found in Nygren et al. (2018) were not studied in depth here and will be the aim of a subsequent study. However, Group B seems to be characterised by having a shorter body and free branchial lobes; these features are shared with *T. atlantis* Williams, 1984 and *T. irinae* Gagaev, 2009 as already suggested by Nygren et al. (2018). Members of Group C are apparently not defined by any unique shared morphological character but show the same geographic distribution as *T. irinae*. Finally, the three putative species in Group D were related to *T. gracilis* Malm, 1874 and *T. williamsae* Jirkov, 1989 by Nygren et al. (2018) even though the latter was proposed to be synonymised with the former by Parapar et al. (2011). These species seem characterised by having ventral white colouration in a number of anterior chaetigers and similar-sized branchial lobes; these characters are not shared with Group A.

Regarding Group A, six morphological characters have been considered to delineate subgroups and species (Table 1). Two characters can be determined with the aid of the STM: 1) general branchial shape, 2) number of thoracic chaetigers with geniculate chaetae; four characters require SEM examination: 3) presence of papillae on lamellae of dorsal branchial lobes, 4) presence of ciliated papillae dorsal to thoracic notopodia, 5) features of thoracic and 6) abdominal uncini shape dentition. Branchial typology (1) is defined according to Parapar et al. (2016c) and thoracic uncini (5) follows Parapar et al. (2020). Typology of abdominal uncini (6) is described here (see Discussion).

Furthermore, species will be also characterised according to geographic and bathymetric distribution according to available data.

### Subgroup A1

Analyses of molecular data found low or no support for monophyly of this clade (Figs 1, 2) and there is no apparent morphological synapomorphy supporting this clade either. Cohesion of members of this group needs to be studied further, but meanwhile, it is considered herein as a morphologically homogenous gathering of species 10–13 and 18–19 (Figs 1, 2). As it was indicated above, only species 10, 11, and 13 will be described herein, of which 10 and 13 are new to science and 11 corresponds to *T. stroemii*; some comments on species 12 (*Terebellides* sp. 1 hereafter) are also provided.

#### Characters present only in subgroup A1

None (Table 1).

#### Character/s shared with subgroup A2

- Branchiae of type 1 (stroemii-type, comma-shaped), all four lobes fused for approximately half of their length and ventral ones usually obscured by dorsal ones (Fig. 11A–C).
- First thoracic neuropodia on TC6, with chaetiger provided with several sharply bent, acute-tipped geniculate chaetae (Figs 6A, 15A, 16B).

#### Character/s shared with subgroup A3

**Figure 7.**
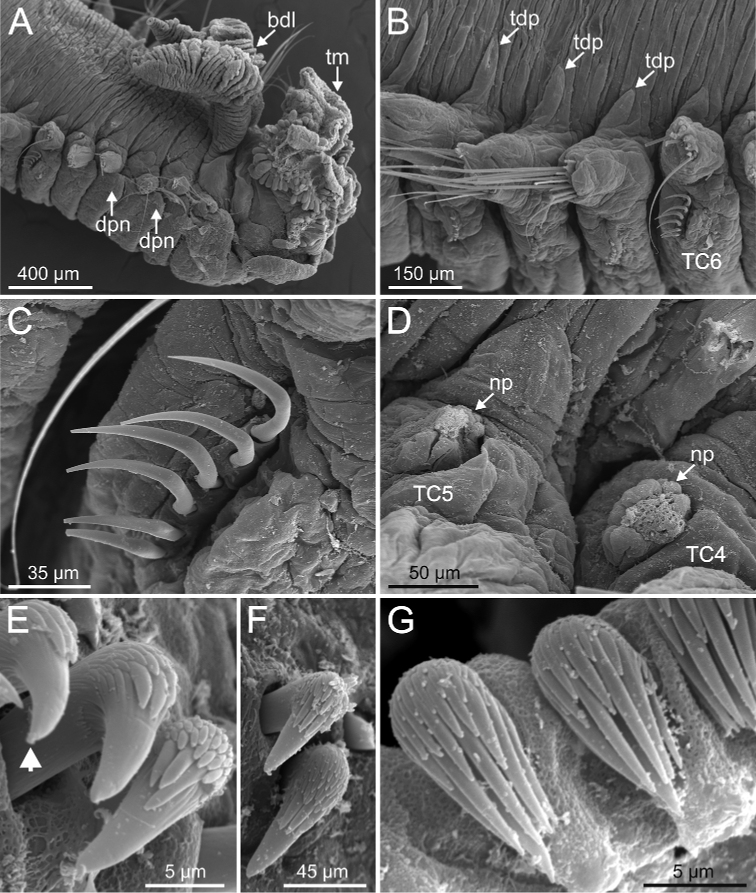
*Terebellides stroemii* Sars, 1835 (species 11; non-type specimen, ZMBN 116399), SEM micrographs. **A** anterior end, right lateral view **B** TC6 to TC8, lateral view **C** geniculate chaetae **D** TC4 and TC5, nephridial papillae **E, F** thoracic uncini (arrow in **E** pointing to rostrum curved at distal end) **G** abdominal uncini. Abbreviations: bdl – branchial dorsal lobes; dpn – dorsal projection of notopodium; np – nephridial papilla; TC – thoracic chaetiger; tdp – thoracic dorsal papilla; tm – tentacular membrane.

- Border of anterior region of dorsal branchial lamellae not provided with papillary projections.
- One ciliated papilla is present, dorsal to thoracic notopodia (Fig. 5F).
- Thoracic uncini type 3 (Figs 6E, 7E, F, 16D).

#### Character/s variable within subgroup A1

- Abdominal uncini type 1 (Fig. 6G) and 2 (Fig. 7G) (see Conclusions Section).

Lavesque et al. (2019) describe several species from French waters similar to those of Group A in terms of body and branchial shape. Among them, *Terebellides gralli* Lavesque, Hutchings, Daffe, Nygren & Londoño-Mesa, 2019 is described as lacking papillary projections on branchial lamellae, but no mention is made to whether or not ciliated papillae are present dorsal to thoracic notopodia. The sequences of this species do not relate with those of any putative species as defined in Nygren et al. (2018). Moreover, *T. gralli* differs morphologically from other congeners in having longer branchiae that may reach TC4–6 (Lavesque et al. 2019: 169, fig. 12A) instead of only reaching TC3–4.

##### Terebellides bakkeni sp. nov.

Taxon classificationAnimaliaTerebellidaTrichobranchidae

505C0C3D-21C6-556E-9A82-9AA68503D265

http://zoobank.org/0D530A3C-65B2-4F9D-A78A-051AE5B62110

Figs 1 , 2 , 3A , 4A , 5 , 6 , 8A , 9 , 17A ; Table 1 ; Suppl. material 1 : Table S1; Suppl. material 2: Table S2

Species 10 Nygren et al. 2018: 18–22, figs 6, 10.

###### Material examined.

**Type material. *Holotype***: ZMBN116395. ***Paratypes*** (10 specimens): Barents Sea (ZMBN116388, ZMBN116389), Norwegian coast and shelf (ZMBN116390, ZMBN116391, ZMBN116392, ZMBN116393, ZMBN116394, ZMBN116396, NTNU–VM61376, NTNU–VM61377).

***Holotype*.** Complete specimen, 32.0 mm long and 2.0 mm width (Figs 3A, 4A).

###### GenBank accession numbers of material examined (COI).

***Holotype***: MG025165; ***Paratypes***: MG025159, MG025160, MG025161, MG025162, MG025163, MG025164, MG025165, MG025166, MG025168, MG025169, MG025170. ***Additional material***: MG025167.

###### Diagnostic features of type material.

Complete individuals ranging from 23.0–32.0 mm in length (Fig. 17A). Branchial dorsal lobes lamellae without papillary projections. Ventral branchial lobes generally hidden behind dorsal ones (Figs 3A, 4A, 5A–C). Lateral lappets and dorsal projection of thoracic chaetigers present on TC2(TC3)–TC5(TC4) (Fig. 5A). Geniculate chaetae in TC6 acutely bent, with low marked capitium (Fig. 6A, B). Ciliated papilla dorsal to thoracic notopodia (Fig. 5F). Thoracic uncini in one row with rostrum/capitium length ratio of approximately 2 : 1 and capitium with a first row of three or four medium-sized teeth, followed by several smaller teeth (Fig. 6C–E). Abdomen with 25–29 pairs of neuropodia (Fig. 6F) with type 1 uncini (Fig. 6G).

###### Nucleotide diagnostic features.

Members of *T. bakkeni* sp. nov. share the following unique nucleotides at these given positions of our alignement: 162 (G), 168 (C), 345 (G; shared only with one specimen from species 17).

###### Type locality.

Nordland, Sortlaandssunder (Lofoten Islands); 119 m deep (Suppl. material 1: Table S1).

###### Distribution and bathymetry.

**Figure 8.**
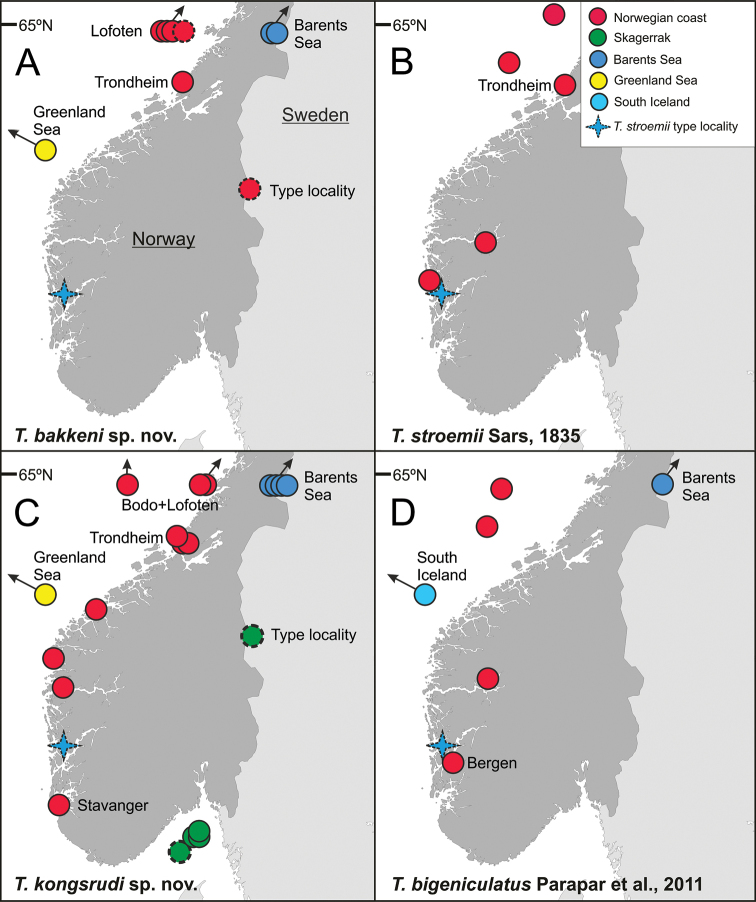
Geographic distribution of **A***T. bakkeni* sp. nov. **B***T. stroemii* Sars, 1835 **C***T. kongsrudi* sp. nov. **D***T. bigeniculatus* Parapar, Moreira & Helgason, 2011.

**Figure 9.**
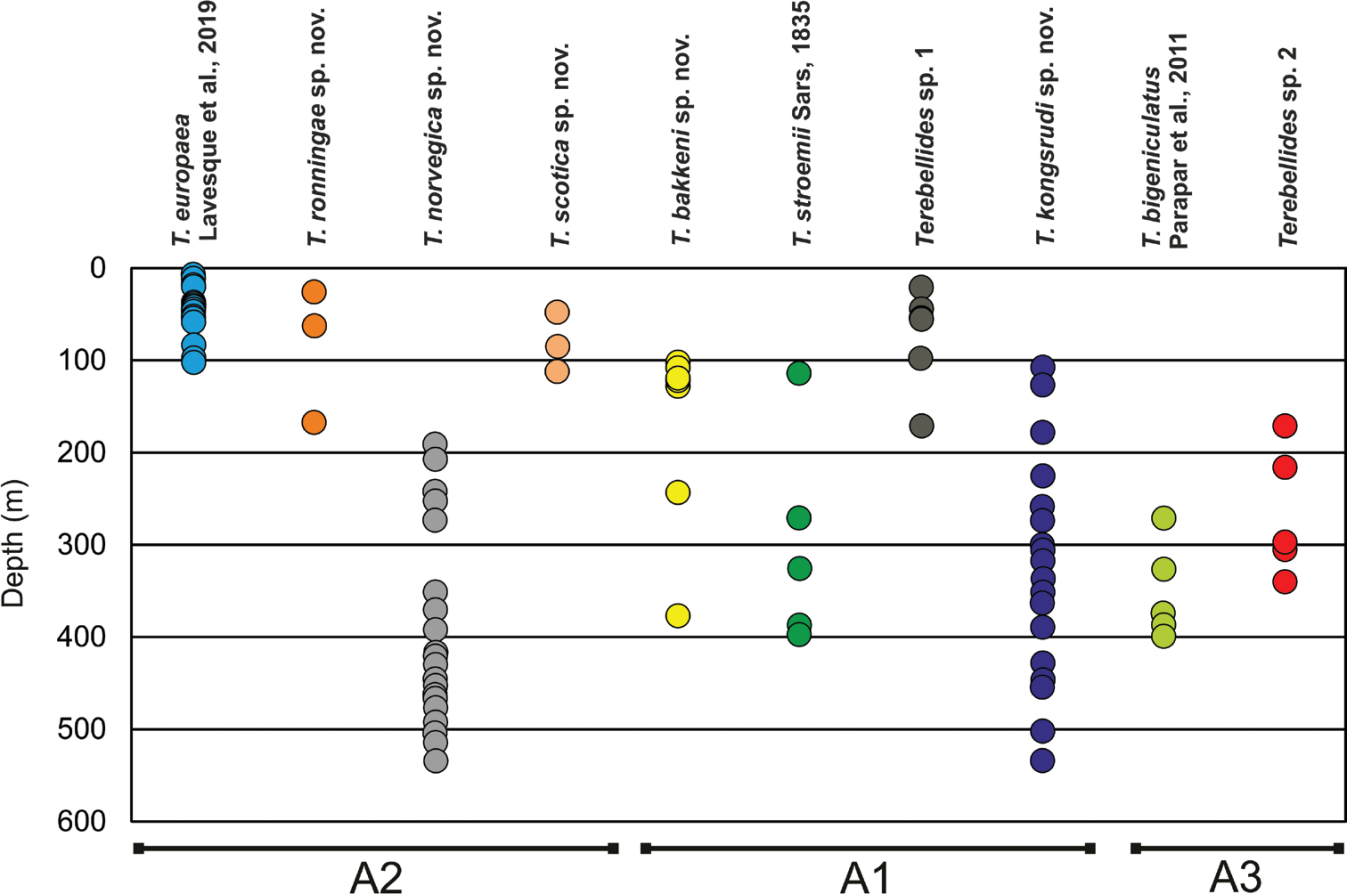
Bathymetric distribution of *Terebellides* species studied in this work. Subgroups (A1–3) within group A sensu Nygren et al. (2018) are indicated.

Barents Sea, Greenland Sea, northern Norwegian coasts from the Lofoten Islands to Trondheim; at depths of102–378 m (Nygren et al. 2018) (Figs 8A, 9; Suppl. material 1: Table S1). One specimen found in North Iceland at 1,250 m deep.

###### Etymology.

This species is named after Dr. Torkild Bakken, from the NTNU–University Museum, Trondheim (Norway), housing institution of some of the specimens used in the present study, for his dedication to the study of Norwegian polychaetes and his friendship.

###### Remarks.

*Terebellides bakkeni* sp. nov. is a small-sized species, maximum-sized specimens reaching 20.0 mm in length (n = 3). This species is characterised by the presence of ciliated papilla dorsal to thoracic notopodia, lack of papillae on the margins of branchial lamellae and presenting abdominal uncini of type 1. Most of these features are also shared by the closest relative, *T. stroemii* (species 11 herein), but they differ in the morphology of the abdominal uncini, being of type 2 in *T. stroemii* and type 1 in *T. bakkeni* sp. nov. (Table 1). One specimen studied with SEM showed ciliary tufts in the inner side of the branchial lamellae (Fig. 5D). If this feature is not an artefact and is confirmed in all members of the species – so far only two specimens were examined under SEM – it would be an autapomorphy for the species. A similar feature was found in the non-closely related *T. gracilis*, that is also present in NEA. The ciliary tufts in *T. bakkeni* sp. nov. are, however, connected by rows of cilia (Fig. 5D), while in *T. gracilis* they are confined to isolated tufts (Parapar et al. 2011: 12, fig. 9c). On the other hand, there are no clear morphological differences between *T. bakkeni* sp. nov. and *T. kongsrudi* sp. nov. (species 13). These sympatric species differ in the southern limit of their geographic distribution: *T. bakkeni* sp. nov., as *T. kongsrudi* sp. nov. are present above 65°N (Fig. 8A, C) while the latter and *T. stroemii* reach more southern latitudes, such as the Skagerrak and Bergen respectively (Fig. 8B, C).

Of the 462 sequences, including all NEA species, and 659 positions in the COI alignment, the 12 sequences assigned to *T. bakkeni* sp. nov. hold two unique nucleotides positions, and an additional one only shared by a single specimen from another clade (see Suppl. material 2: Table S2). The species also showed 0–1.9% of intraspecific divergence in the COI marker, and a minimum of 11.5% uncorrected genetic distance with congeners (in this case *T. stroemii*) (Nygren et al. 2018).

##### Terebellides stroemii

Taxon classificationAnimaliaTerebellidaTrichobranchidae

Sars, 1835

4AB21008-C857-5292-8E8C-6076C4AC2266

Figs 1 , 2 , 3B , 4B , 7 , 8B , 9 , 10 , 17A , 28D ; Suppl. material 1 : Table S1; Suppl. material 2: Table S2

Terebellides stroemii Sars, 1835: 48–50, pl. 13, fig. 31a–e. Parapar and Hutchings 2014: 10, fig. 5–10. *Non*Parapar et al. 2011: 14–17, figs 11, 12, 13G.
Species 11 – Nygren et al. 2018: 18–22, figs 6, 10. *Non* Clade 6 in Nygren et al. (2018) (see Remarks).

###### Type locality.

Helle, Manger, Bergenfjord (Norway) (Parapar and Hutchings 2014).

###### Material examined.

5 specimens (Suppl. material 1: Table S1), Norwegian coast and shelf: ZMBN 116397, ZMBN 116398, ZMBN 116399, ZMBN 116400, ZMBN 116401.

###### A﻿dditional material.

**Neotype** (NHMOC5896) and seven “**neoparatypes**” (NHMOC5899, NHMOC5902, NHMOC5904, NHMOC5905, NHMOC5907, NHMOC5956, NHMOC5968) of *T. stroemii* (Suppl. material 1: Table S1).

###### GenBank accession numbers of material examined (COI).

MG025171, MG025172, MG025173, MG025174, MG025175.

###### Diagnostic features of studied material.

Complete individuals ranging from 6.0–20.0 mm in length (Fig. 17A). Branchial dorsal lobes lamellae without papillary projections. Ventral branchial lobes hidden behind dorsal lobes (Figs 3B, 4B). Lateral lappets present on TC1–TC4; dorsal projection well marked from TC3–TC4 (Fig. 7A). Geniculate chaetae in TC6, acutely bent (Fig. 7C) with low marked capitium. Ciliated papilla dorsal to thoracic notopodia (Fig. 7B). Thoracic uncini in one row with rostrum/capitium length ratio approximately 2 : 1 and capitium with a first row of three or four medium-sized teeth, followed by several smaller teeth (Fig. 7E, F). Abdomen with 23–32 chaetigers (Fig. 17A) with type 2 uncini (Figs 7G, 28D).

###### Nucleotide diagnostic features.

There are no unique apomorphic nucleotides in the fragments of COI analysed for *T. stroemii*, when considering all *Terebellides* species present in the NEA (Suppl. material 2: Table S2). However, when comparing homologous nucleotide positions with members of only Group A (183 sequences in the COI alignment), the following autapomorphies arise: 174 (C), 183 (C), 453 (A), 612 (C).

###### Distribution and bathymetry.

**Figure 10.**
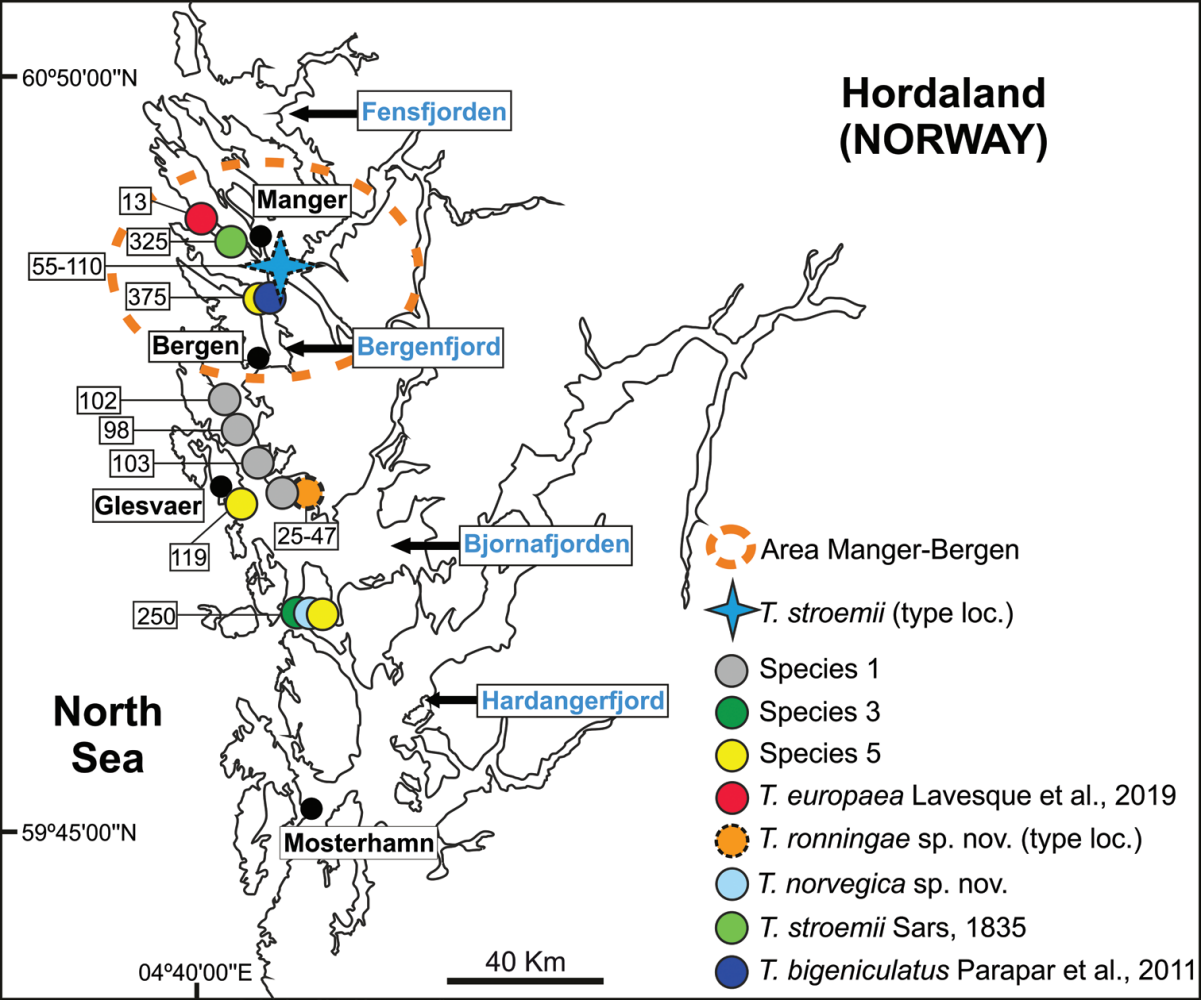
Map of Hordaland area (SW Norway) showing collecting sites of *Terebellides* species as found in Nygren et al. (2018) near type locality of *T. stroemii* Sars, 1835. Depth ranges shown in boxes.

*Terebellides stroemii* was traditionally considered as a cosmopolitan species, but its known distribution seems in fact restricted to the Norwegian coastline (Parapar et al. 2011; Parapar and Hutchings 2014; Lavesque et al. 2019). Specimens examined by Nygren et al. (2018) and in the present paper, obtained after comprehensive sampling in the NEA, were found only in W Norway, between 115 and 388 m deep (Figs 8B, 10; Suppl. material 1: Table S1).

###### Remarks.

In the five sequences belonging to this species, there were four haplotypes showing 0–1.1% of intraspecific divergence, and a minimum of 11.5% uncorrected genetic distance with members of the closest relative, *T. bakkeni* sp. nov. (Nygren et al. 2018).

*Terebellides stroemii* is a large species, reaching up to 52 mm in length (Parapar and Hutchings 2014) and is characterised by the presence of ciliated papilla dorsal to thoracic notopodia, lack of papillae on margins of branchial lamellae, thoracic uncini of type 3 and abdominal uncini of type 2. All these features are shared with *T. kongsrudi* sp. nov.; *T. bakkeni* sp. nov. is also very close morphologically to *T. stroemii* but they differ in the morphology of the abdominal uncini as explained above.

Nygren et al. (2018) misidentified species 6 as *T. stroemii*, but this was later corrected by Lavesque et al. (2019) who pointed out that the molecular sequences of these specimens fit with those of *T. europaea*.

Specimens examined here bear thoracic uncini that are most similar to other members of Group A; SEM examination showed, however, that some uncini have a rostrum distal tip that is distinctly bent downwards (deformity?) (Fig. 7E, arrow) as already described for the type specimens by Parapar and Hutchings (2014: 8, fig. 7F, G), and attributed to preservation for too long in EtOH. However, we have found similar bent rostrum among specimens of *T. kongsrudi* sp. nov. (Fig. 12D, arrow), *T. ronningae* sp. nov. (species 7) (Fig. 21C, arrows) and *T. bigeniculatus* (species 20 + 28) (Fig. 26E, frame) suggesting this may not be related to preservation. The abdominal uncini are quite similar to those described in Parapar and Hutchings (2014: 9, fig. 8C–E) also showing a small gap among the anteriormost teeth of rostrum (Parapar and Hutchings 2014: 8–9, fig. 8F; Fig. 7G); these features are not shared by other species of subgroup A1, i.e., *T. bakkeni* sp. nov. and *T. kongsrudi* sp. nov. In all, species 11 agrees well with the redescription of *T. stroemii*.

Geographic and bathymetric distribution of our specimens also agree with that of *T. stroemii* (see Parapar and Hutchings 2014), with Manger (Norway) (i.e., type locality of *T. stroemii*; Fig. 10) being its southernmost distribution limit. The other three taxa, i.e., species 5, *T. europaea* and *T. bigeniculatus*, were also found near Manger, but all can be clearly distinguished morphologically from each other (see above and below for *T. europaea* and *T. bigeniculatus*) and species 5 belongs to Group B and seems closer morphologically to *T. atlantis*. On the other hand, type specimens of *T. stroemii* come from depths of 55–110 m (Parapar and Hutchings 2014) as well as specimens belonging to *T. europaea*, *T. ronningae* sp. nov., *T. scotica* sp. nov. (species 9) and species 12 (<200 m), and therefore they seem to constitute a shallow-water assemblage of species from an ecological point of view.

Finally, the Icelandic specimens reported as *T. stroemii* by Parapar et al. (2011) might not correspond to this species. In fact, it is likely that they represent at least two different species, namely *T. bakkeni* sp. nov. and *T. kongsrudi* sp. nov., both reported here to the North and East of Iceland. Therefore, the aforementioned specimens deserve further revision.

##### Terebellides kongsrudi sp. nov.

Taxon classificationAnimaliaTerebellidaTrichobranchidae

07813522-4D07-54F7-A003-46F128BD706F

http://zoobank.org/541890B5-C55E-4716-BB42-0D87E7184885

Figs 1 , 2 , 3C , 4C , 8C , 9 , 11 , 12 , 17B , 28A ; Table 1 ; Suppl. material 1 : Table S1; Suppl. material 2: Table S2

Species 13 – Nygren et al. 2018: 18–22, figs 6, 10.

###### Material examined.

**Type material. *Holotype***: GNM14632. ***Paratypes*** (20 specs): Barents Sea (ZMBN116409, ZMBN116411, ZMBN116414); Norwegian coast and shelf (ZMBN116412, ZMBN116413, ZMBN116415, ZMBN116416, ZMBN116417, ZMBN116418, NTNU-VM66568, NTNU-VM66570, NTNU-VM66571, NTNU-VM66572, NTNU-VM68195, NTNU-VM72560, NTNU-VM72561, NTNU-VM72562, NTNU-VM72563); Skagerrak (GNM15136, GNM14632, GNM14638).

***Holotype*.** Complete specimen, 50.0 mm long and 5.0 mm width (Figs 3C, 4C).

###### GenBank accession numbers of material examined (COI).

Paratypes: MG025201, MG025202, MG025203, MG025204, MG025210, MG025211, MG025212, MG025214, MG025216, MG025217, MG025218, MG025219, MG025223. Additional material: MG025199, MG025200, MG025205, MG025206, MG025207, MG025208, MG025209, MG025213, MG025215, MG025220, MG025221, MG025222, MG025224.

###### Diagnostic features of type material.

**Figure 11.**
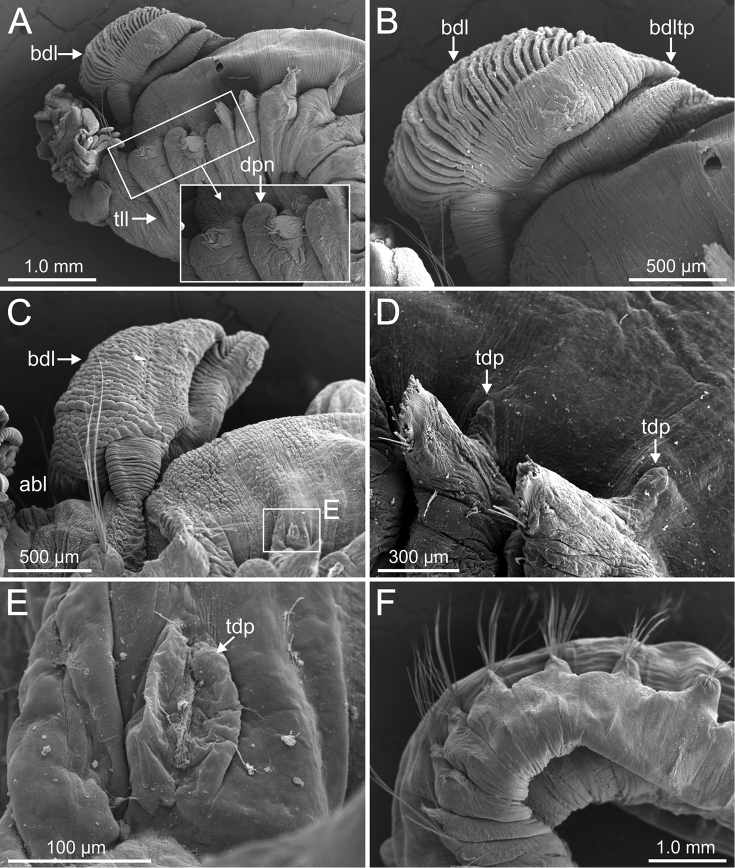
*Terebellides kongsrudi* sp. nov. (species 13; paratypes, ZMBN 116409 and ZMBN 116411), SEM micrographs. **A** anterior end, left lateral view **B** branchiae, left side **C** anterior end, left lateral view **D** TC1 and TC2, thoracic dorsal papillae **E** TC3, thoracic dorsal papilla (framed in **C**) **F** several thoracic chaetigers, left lateral view. Abbreviations: abl – anterior branchial lobe; bdl – branchial dorsal lobe; bdltp – branchial dorsal lobe terminal papilla; dpn – dorsal projection of notopodium; tdp – thoracic dorsal papilla; tll – thoracic lateral lobes.

**Figure 12.**
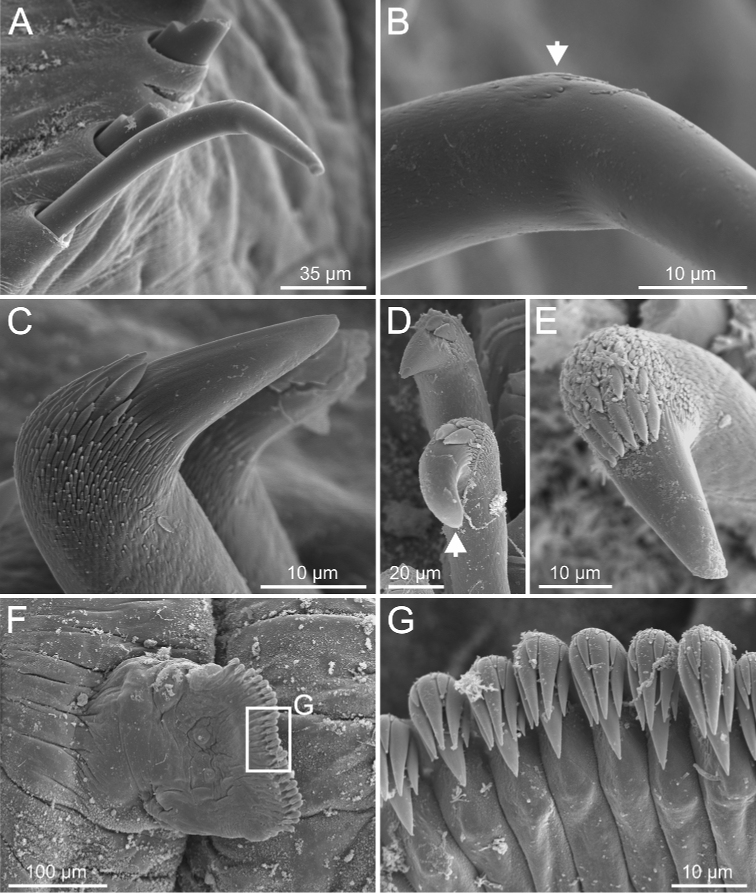
*Terebellides kongsrudi* sp. nov. (species 13; paratype, ZMBN 116409), SEM micrographs. **A** TC6 (TU1) geniculate chaeta **B** detail of geniculate chaeta (arrow pointing to capitium) **C–E** thoracic uncini, lateral and frontal views (arrow in **D** pointing to rostrum curved at distal end) **F** abdominal unciniger **G** abdominal uncini, frontal view (framed in **F**).

Complete individuals 12.0–50.0 mm in length (Fig. 17B). Branchial dorsal lobes lamellae without papillary projections. Ventral branchial lobes hidden in between dorsal ones (Figs 3C, 4C, 11A–C). Lateral lappets and dorsal projection of thoracic notopodia on TC2(3)–TC5(4) (Fig. 11A). Geniculate chaetae in TC6, acutely bent, with low marked capitium (Fig. 12A, B). Two pairs of nephridial pores in TC4 and TC5 and ciliated papilla dorsal to thoracic notopodia (Fig. 11D, E). Thoracic uncini in one row with rostrum/capitium length ratio approximately 2 : 1 and capitium with a first row of 2–5 medium-sized teeth, followed by several smaller teeth (Fig. 12C–E). Abdomen with 25–35 uncinigers (Fig. 12F) with type 1 uncini (Figs 12G, 28A).

###### Nucleotide diagnostic features.

All sequences of *T. kongsrudi* sp. nov. share the unique apomorphic nucleotides in positions 300 (G) and 624 (G) of our alignement.

###### Type locality.

Skagerrak; 429–445 m deep (Fig. 8C; Suppl. material 1: Table S1).

###### Distribution and bathymetry.

Barents Sea, Greenland Sea, along the Norwegian coast and shelf, reaching the Skagerrak to the South; 108–534 m deep (Nygren et al. 2018) (Figs 8C, 9; Suppl. material 1: Table S1).

###### Etymology.

This species is named after Dr. Jon Anders Kongsrud, Department of Natural History, Zoological Museum Bergen–ZMB (Norway), housing institution of some of the specimens used in the present study, for his dedication to the study of Norwegian polychaetes and his friendship.

###### Remarks.

This is a large species reaching up to 50.0 mm long, and is characterised by the presence of ciliated papilla dorsal to thoracic notopodia, lack of papillae on the margins of branchial lamellae, thoracic uncini of type 3 and abdominal uncini of type 1. These features are also shared by species 12 (sensu Nygren et al. 2018), which will be described elsewhere (Gaeva and Jirkov, pers. comm.). *Terebellides kongsrudi* sp. nov. is also morphologically similar to *T. bakkeni* sp. nov. (see above) but *T. kongsrudi* sp. nov. and species 12 show a wider geographic distribution; on the contrary, species 12 is present at shallower depths (<200 m) while *T. kongsrudi* sp. nov. extends to deeper depths (>500 m).

Finally, in the 26 sequences belonging to this species (see Suppl. material 2: Table S2), there were fourteen haplotypes showing 0–1.9% of intraspecific divergence, and a minimum of 8.2% uncorrected genetic distance with members of species 12 which is the closest relative (sensu Nygren et al. 2018).

##### Terebellides

Taxon classificationAnimaliaTerebellidaTrichobranchidae

sp. 1

631CD078-2A77-590F-A839-68A38A4BF1F9

**Figure 13.**
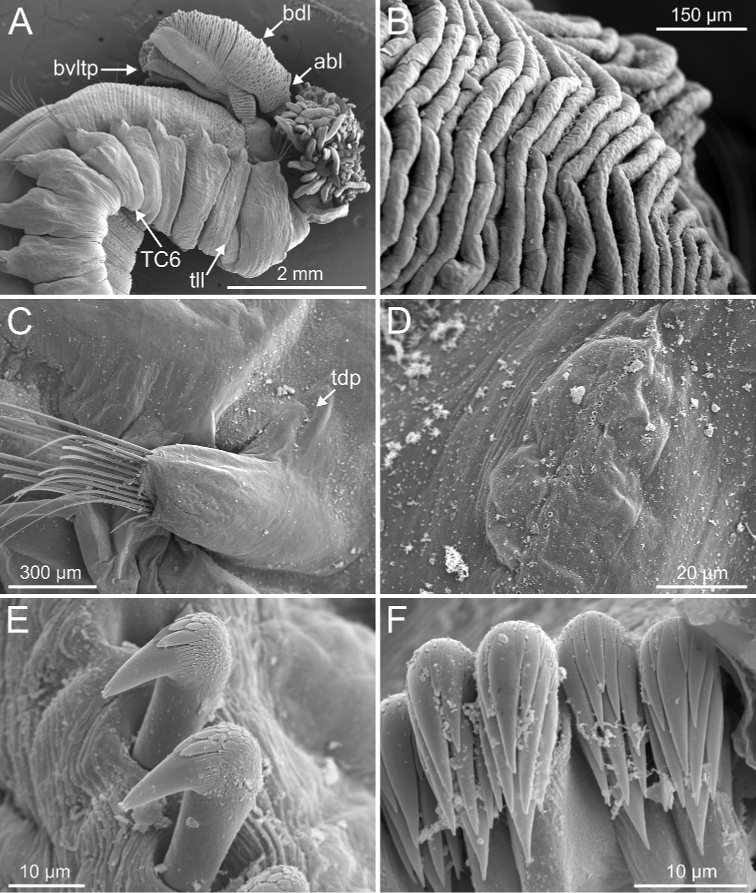
*Terebellides* sp. 1 (species 12; GNM 14630-4 and GNM 14640-8), SEM micrographs. **A** anterior end, right lateral view **B** detail of anterior branchial lamellae **C** TC16 **D** notopodial papilla **E** thoracic uncini **F** abdominal uncini. Abbreviations: abl – anterior branchial lobe; bdl – branchial dorsal lobe; bvltp – branchial ventral lobe terminal papilla; TC – thoracic chaetiger; tdp – thoracic dorsal papilla; tll – thoracic lateral lobes.

Figs 1 , 2 , 9 , 13 ; Table 1 ; Suppl. material 1 : Table S1; Suppl. material 2: Table S2

Species 12 – Nygren et al. 2018: 18–22, figs 5, 6, 10.

###### Material examined.

4 specimens. **Skagerrak**. GNM 14630-4; GNM 14630-8.

###### Remarks.

This species will be described elsewhere by D. Gaeva and I. Jirkov (pers. comm.). In order to confirm characters here used to link species within each subgroup, two specimens were examined under the SEM that share with subgroup A1 the following features: branchiae type 1 sensu Parapar et al. (2016c) (Fig. 13A), lack of papillae on border of branchial lamellae (Fig. 13B), geniculate chaetae on TC6, ciliated papilla dorsal to thoracic notopodia (Fig. 13C, D), and thoracic uncini of type 3 (Fig. 13E). Nevertheless, abdominal uncini are of type 2 (Fig. 13F), as it occurs in *T. stroemii* and differently to *T. bakkeni* sp. nov. and *T. kongsrudi* sp. nov., that are the most similar species within subgroup A1 (Table 1).

### Subgroup A2

Molecular analyses of mitochondrial and nuclear markers recovered a strongly supported subgroup A2 (Fig. 1). This subgroup is composed by species 6, 7, 8, and 9 (sensu Nygren et al. 2018). Analyses of the COI dataset alone also find support for this clade, and incorporate the recently described *T. lilasae* Lavesque, Hutchings, Daffe, Nygren & Londoño-Mesa, 2019 (Fig. 2). There are several morphological features that are shared, and exclusive to, all members of subgroup A2, and includes other NEA species (see below). Three (7, 8, 9) of these four species are described herein as new to science and the fourth species (6) corresponds to *T. europaea*.

#### Character/s present only in Group A2

- Border of anterior region of dorsal branchial lamellae provided with papillary projections (Figs 15C, 20C, 22C).
- Ciliated papilla dorsal to thoracic notopodia not present.
- Abdominal uncini type 2 (Figs 16E, 21F, 23E, 25F).

#### Character/s shared with subgroup A1

- Branchiae of type 1 (stroemii-type, comma-shaped), all four lobes fused for approximately half of their length and ventral ones usually obscured by dorsal ones (Fig. 20A).
- First thoracic neuropodia on TC6, with chaetiger provided with several sharply bent, acute-tipped geniculate chaetae (Figs 15A, 16B).

#### Character/s shared with subgroup A3

None (Table 1).

#### Character/s variable within subgroup A2

- Thoracic uncini type 1 and 3 (Figs 21E, 16D).

Several species described by Lavesque et al. (2019) have a similar body and branchiae appearance to those of subgroup A2 species; however, only four species bear papillae on the anterior border of branchial lamellae: *Terebellides bonifi* Lavesque, Hutchings, Daffe, Nygren & Londoño-Mesa, 2019, *T. europaea*, *T. gentili* Lavesque, Hutchings, Daffe, Nygren & Londoño-Mesa, 2019 and *T. lilasae*. Molecular sequences were available for all except *T. gentili*, with *T. europaea* being the only species found among the material sequenced and analysed by Nygren et al. (2018), as species 6, and initially misidentified as *T. stroemii*.

*Terebellides gentili* does not fit morphologically within any clade defined here because of having numerous marginal branchial lamellae that reach the posterior end of dorsal lobes, the dorsal lobes are longer and reach TC5(TC6) instead of TC3(TC4), and TC3 has a distinct whitish glandular region with a well-defined central white line. On the contrary, *T. lilasae* was found within subgroup A2 according to molecular-based analyses (Fig. 2); this species also fits well morphologically in A2 by having similar branchiae (shape), papillae on branchial lamellae, thoracic uncini of type 3 and abdominal uncini of type 2, only differing in having comparatively larger branchiae. The original description does, however, not mention whether notopodial papillae are present or not. This species was described from the French Mediterranean and Atlantic waters and is not present in northern latitudes, as suggested by Lavesque et al. (2019) and confirmed here. On the other hand, *T. bonifi* bears similar branchiae (shape, size, papillae) and thoracic uncini of type 3 (Lavesque et al. 2019: 159, fig. 4A–C) to those of A2; however, it bears abdominal uncini of type 1 instead of type 2.

##### Terebellides europaea

Taxon classificationAnimaliaTerebellidaTrichobranchidae

Lavesque, Hutchings, Daffe, Nygren & Londoño-Mesa, 2019

F2914BAE-42BF-5749-86BA-06CE82A50BDD

Figs 1 , 2 , 3E , 9 , 10 , 14A , 15 , 16 , 17C , 18A , 19A ; Table 1 ; Suppl. material 1 : Table S1; Suppl. material 2: Table S2

Terebellides europaea Lavesque et al. 2019: 163–165, figs 1, 7, 8.
Species 6 – T. stroemii (*non* Sars, 1835). Nygren et al. 2018: 18–22, figs 6, 10.

###### Material examined.

31 specimens: Norwegian coast and shelf (GNM14625, GNM14628, GNM15107, GNM15114, GNM15115, GNM15116, GNM15120, GNM15121, GNM15122, GNM15123, GNM15124, GNM15125, GNM15126, GNM15127, GNM15128, ZMBN116334, ZMBN116335, ZMBN116343, ZMBN116344, ZMBN116346, ZMBN116347); Irish Sea (ZMBN116336, ZMBN116337, ZMBN116338, ZMBN116339, ZMBN116340, ZMBN116341, ZMBN116342).

###### GenBank accession numbers of material examined (COI).

MG025072, MG025073, MG025074, MG025075, MG025076, MG025077, MG025078, MG025079, MG025080, MG025081, MG025082, MG025083, MG025084, MG025085, MG025086, MG025087, MG025088, MG025089, MG025090, MG025091, MG025092, MG025093, MG025094, MG025095, MG025096, MG025097, MG025098, MG025099, MG025100, MG025101, MG025102, MG025103, MG025104. Paratypes (not examined): MN207179, MN207181. Additional sequences (material not examined): MN207180, MN207182.

###### Diagnostic features of type material.

**Figure 14.**
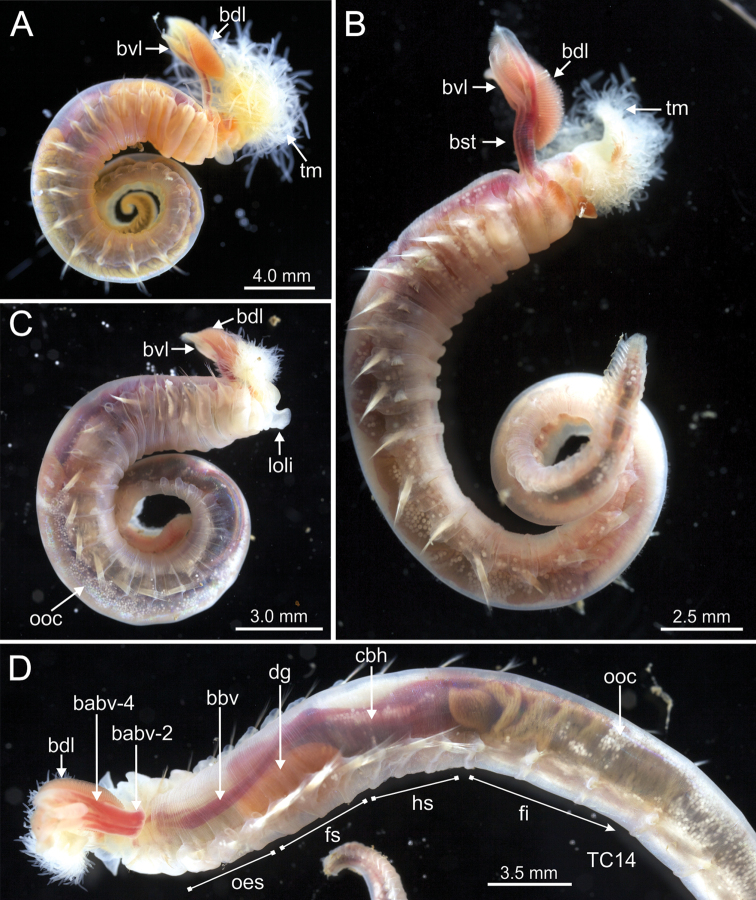
STM photographs of live specimens of several *Terebellides* species in lateral view. **A***Terebellides europaea* Lavesque, Hutchings, Daffe, Nygren & Londoño-Mesa, 2019 (ZMBN 116343) **B***Terebellides ronningae* sp. nov. (ZMBN 116349) **C, D***Terebellides norvegica* sp. nov. (GNM 15131 and GNM 15130 respectively). Abbreviations: babv – branchial afferent blood vessel; bbv – branchial blood vessel; bdl – branchial dorsal lobe; bst – branchial stem; bvl – branchial ventral lobes; cbh – contractile branchial heart; dg – digestive gland; fi – fore intestine; fs – fore stomach; hs – hind stomach; loli – lower lip; oes – oesophagus; ooc – oocytes; tm – tentacular membrane.

**Figure 15.**
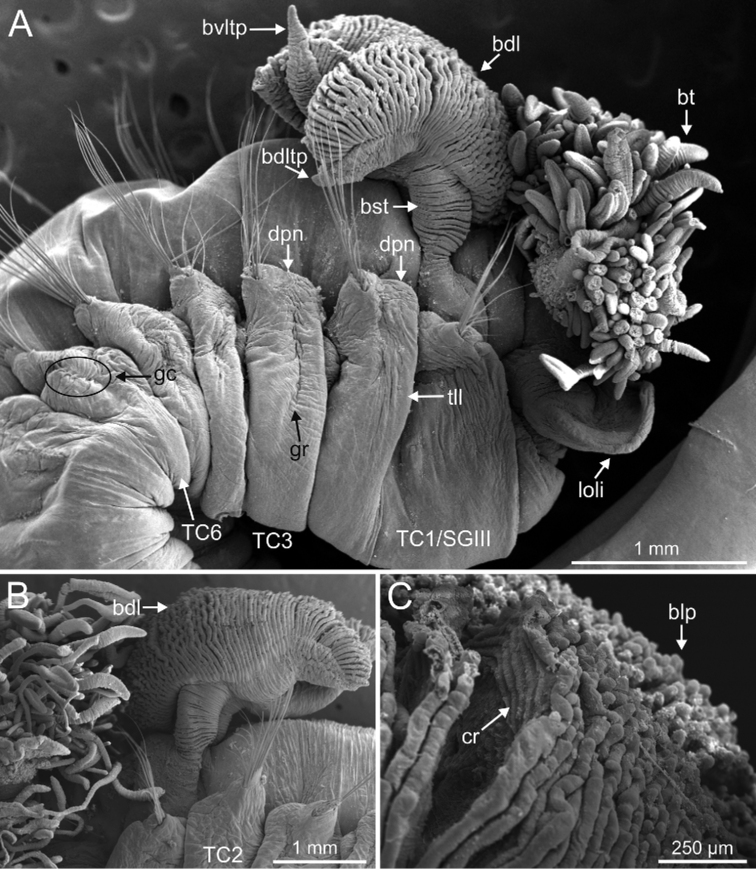
*Terebellides europaea* Lavesque, Hutchings, Daffe, Nygren & Londoño-Mesa, 2019 (species 6; non-type specimens, GNM15116 and GNM15118), SEM micrographs. **A** anterior end, right lateral view **B** buccal tentacles and branchiae, left lateral view **C** branchial lamellae, detail. Abbreviations: bdl – branchial dorsal lobe; bdltp – branchial dorsal lobe terminal papilla; blp – branchial lamellae papillae; bst – branchial stem; bt – buccal tentacles; bvltp – branchial terminal lobe terminal papilla; cr – ciliary row; dpn – dorsal projection of notopodium; gc – geniculate chaetae; gr – glandular region; loli – lower lip; SG – segment; TC – thoracic chaetiger; tll – thoracic lateral lobes.

**Figure 16.**
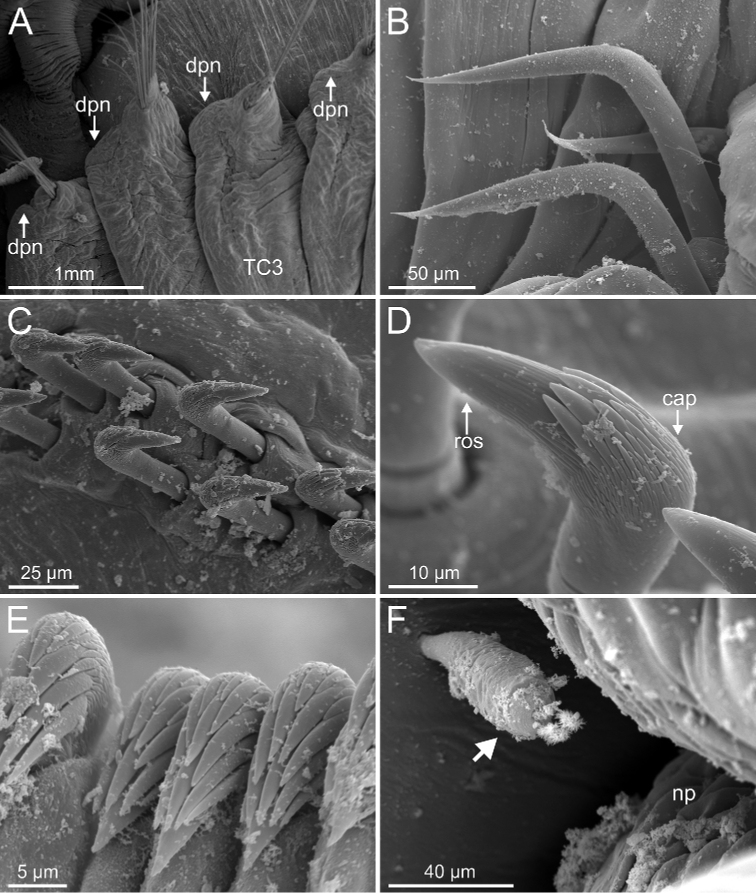
SEM images, *Terebellides europaea* Lavesque, Hutchings, Daffe, Nygren & Londoño-Mesa, 2019 (species 6; non-type specimen, GNM15116). **A** TC1 to TC4, lateral view **B** TC6 (TU1), geniculate chaetae **C** thoracic double row of uncini **D** thoracic uncinus, capitium, upper view **E** abdominal uncini **F** epibiont ciliate (position pointed by arrowhead) attached near TC5 nephridial papilla. Abbreviations: cap – capitium; dpn – dorsal projection of notopodium; ros – rostrum; TC – thoracic chaetiger.

**Figure 17.**
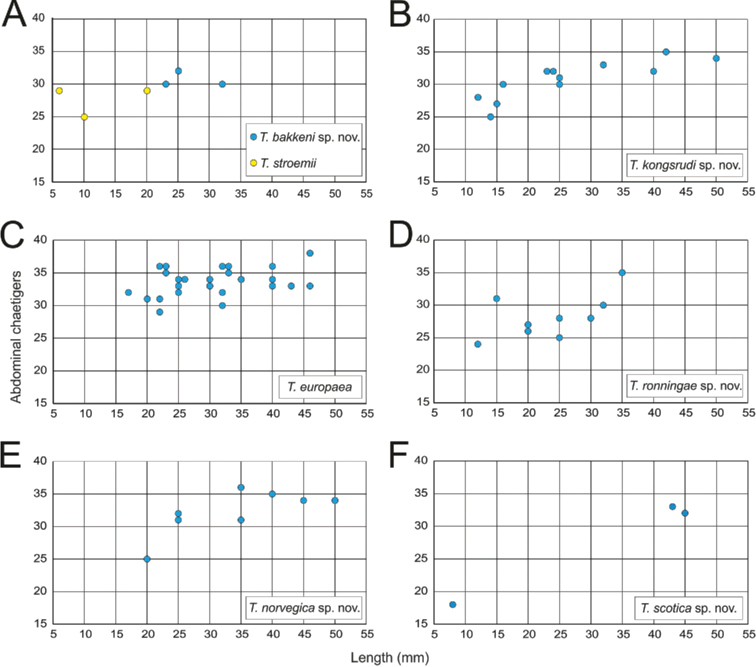
Relationship between number of abdominal chaetigers and body length (complete specimens) for *Terebellides* species described in this work.

Complete individuals ranging from 17.0–46.0 mm in length and 2.0–5.0 mm in width (Fig. 17C). Branchial dorsal lobes lamellae provided with well-developed anterior papillary projections (Fig. 15C). Ventral branchial lobes normally hidden by dorsal ones (Figs 3E, 15B, 19A) but sometimes discernible below (Fig. 14A). Lateral lappets and dorsal projection on thorax present on TC1–TC4 (Fig. 16A) or TC2–TC3 in (Fig. 15A). Geniculate chaetae acutely bent (Fig. 16B). Ciliated papilla dorsal to thoracic notopodia not observed (Figs 15A, 16A). Thoracic uncini in one or two rows (Fig. 16C) with rostrum/capitium length ratio for approximately 2 : 1 (Fig. 16D), and capitium with a first row of four medium-sized teeth, followed by several smaller teeth. Abdomen with 29–38 uncinigers provided with type 2 uncini (Fig. 16E). Epibiont ciliates observed in some specimens (Fig. 16F).

###### Nucleotide diagnostic features.

All sequences belonging to *T. europaea* share the unique apomorphic nucleotide in position 240 (C) of the alignement.

###### Type locality.

Bay of Brest (Brittany, France) (Lavesque et al. 2019).

###### Distribution and bathymetry.

**Figure 18.**
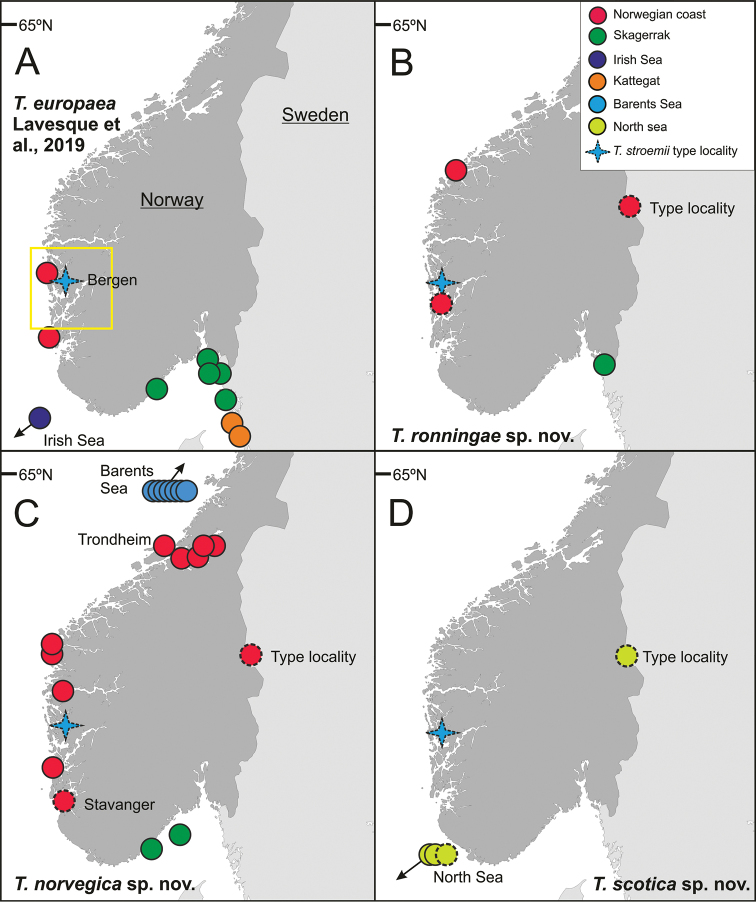
Geographic distribution of **A***T. europaea*Lavesque et al., 2019, **B***T. ronningae* sp. nov., **C***T. norvegica* sp. nov., **D***T. scotica* sp. nov. Yellow frame showing Hordaland (Fig. 10).

Bay of Biscay (Lavesque et al. 2019); Kattegat, Skagerrak, North Sea, Irish Sea, Celtic Sea and Norwegian coast and shelf, 8–173 m deep (Nygren et al. 2018) (Figs 9, 10, 18A; Suppl. material 1: Table S1). Lavesque et al. (2019) included the Ría de Ferrol (Galicia, NW Spain) as part of the Bay of Biscay, but this locality belongs to the northern Galician Rias that are out of the western limit of this bay.

###### Remarks.

This species is characterised by the combination of the following features: presence of papillary projections over the edge of the anterior border of dorsal branchial lamellae, lack of ciliated papilla dorsal to thoracic notopodia, thoracic uncini of type 3 and abdominal uncini of type 2. The original description states that body length is less than 17 mm, but maximal length of specimens examined here was up to 46.0 mm. Examination of live and preserved specimens has revealed that the size ratio between the ventral and dorsal branchial lobes is similar in all specimens; however, their arrangement differs among specimens, i.e., the ventral lobes are visible in some while in others are hidden behind the dorsal lobes.

*Terebellides europaea* was misidentified as *T. stroemii* by Nygren et al. (2018; species 6) due to their morphological similarities and coexistence near the type locality of the latter (Fig. 9). Nevertheless, Lavesque et al. (2019) found that members of species 6 have papillae on the edge of the dorsal branchial lobes, unlike the neotypes of *T. stroemii* described by Parapar and Hutchings (2014). Molecular analyses show that the sequences of specimens found in the Bay of Biscay belong to species 6 (Lavesque et al. 2019); examination of all specimens also confirmed the presence of the aforementioned papillae. Moreover, *T. europaea* is generally found in bottoms above 100 m deep while *T. stroemii* is present in deeper environments (>100 m) (Fig. 9).

In the 37 sequences analysed attributed to this species (see Suppl. material 2: Table S2), there were ten haplotypes showing 0–0.8% of intraspecific divergence, and a minimum of 8.8% uncorrected genetic distance with members of the closest relative, *T. ronningae* sp. nov.

##### Terebellides ronningae sp. nov.

Taxon classificationAnimaliaTerebellidaTrichobranchidae

3727819C-0357-5682-94EA-4F34A3ED8A51

http://zoobank.org/7A447FDE-5934-483F-95F3-D178A0857A4A

Figs 1 , 2 , 3F , 9 , 10 , 14B , 17D , 18B , 19B , 20 , 21 , 28C ; Table 1 ; Suppl. material 1 : Table S1; Suppl. material 2: Table S2

Species 7 – Nygren et al. 2018: 18–22, figs 5, 6, 10, Suppl. material 1: Table S1.

###### M﻿aterial examined.

**Type material. *Holotype***: ZMBN116357. ***Paratypes*** (8 specs): Norwegian coast (ZMBN 116350, ZMBN 116352, ZMBN 116353, ZMBN 116354, ZMBN 116355, ZMBN 116356, ZMBN 116358, ZMBN 116359); Skagerrak (ZMBN 116348, ZMBN 116349).

**Figure 19.**
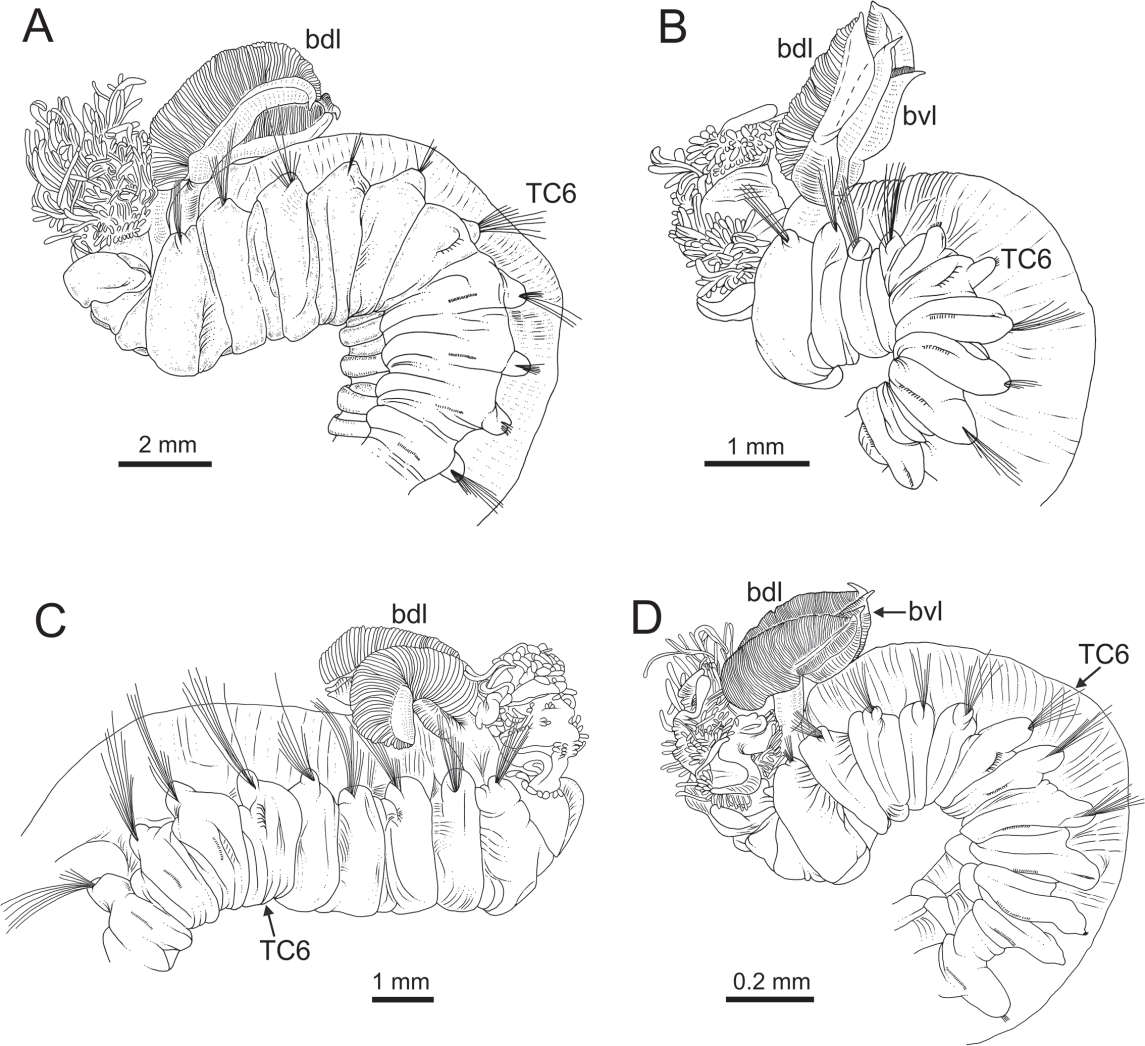
Line drawings of several *Terebellides* species. **A***Terebellides europaea* Lavesque, Hutchings, Daffe, Nygren & Londoño-Mesa, 2019 (species 6; non-type specimen, GNM14628), anterior end, left lateral view **B***Terebellides ronningae* sp. nov. (species 7; holotype, ZMBN116357), anterior end, left lateral view **C***Terebellides norvegica* sp. nov. (species 8; holotype, ZMBN416378), anterior end, right lateral view **D***Terebellides scotica* sp. nov. (species 9; holotype, ZMBN116385), anterior end, left lateral view. Abbreviations: bdl – branchial dorsal lobe; bvl – branchial ventral lobe; TC – thoracic chaetiger.

***Holotype*.** Complete specimen, 19.0 mm long and 2.0 mm width (Figs 3F, 19B).

###### GenBank accession numbers of material examined (COI).

***Holotype***: MG025114; ***Paratypes***: MG025105, MG025106, MG025107, MG025109, MG025110, MG025111, MG025112, MG025113, MG025115, MG025116. ***Additional material***: MG025108,

###### Diagnostic features of type material.

**Figure 20.**
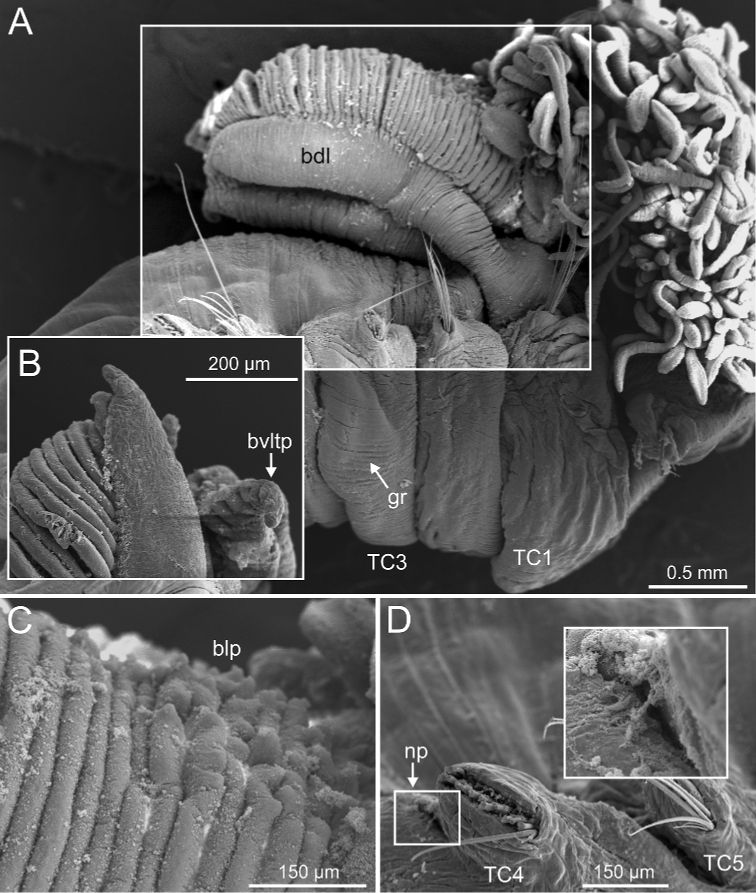
*Terebellides ronningae* sp. nov. (species 7; paratypes, ZMBN 116349 and ZMBN 116353), SEM micrographs. **A** anterior end, right lateral view **B** dorsal branchial lobes, terminal papilla **C** anterior branchial lamellae papillae **D** TC4, nephridial papilla (framed: detail). Abbreviations: bdl – branchial dorsal lobe; blp – branchial lamellae papillae; bvltp – branchial ventral lobe terminal papilla; gr – glandular region; np – nephridial papilla; TC – thoracic chaetiger.

**Figure 21.**
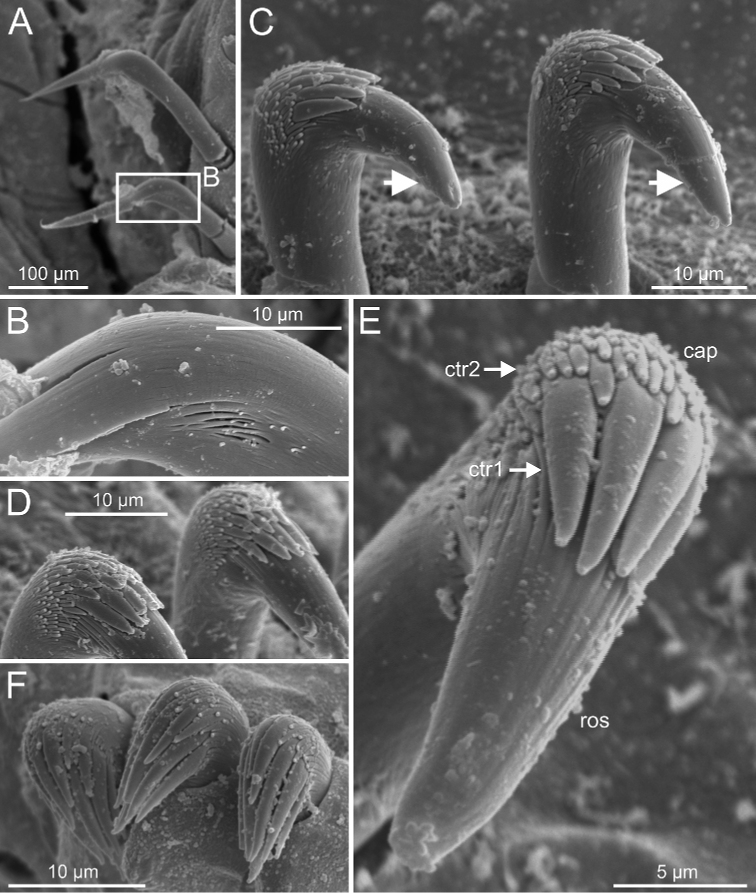
*Terebellides ronningae* sp. nov. (species 7; paratypes, ZMBN 116349 and ZMBN 116353), SEM micrographs. **A** TC6 (TU1), geniculate chaetae **B** geniculate chaeta, detail (framed in **A**) **C–E** thoracic uncini (arrows in **C** pointing to rostrum curved at distal end) **F** abdominal uncini. Abbreviations: cap – capitium; ctr1/2 – first and second rows of capitium teeth; ros – rostrum.

Complete individuals ranging from 12.0–35.0 mm in length and 1.5–3.0 mm in width (Fig. 17D). Branchial dorsal lobes lamellae with poorly-developed anterior papillary projections (Fig. 20C). Ventral branchial lobes hidden (Fig. 20A) or not (Figs 3F, 19B) by dorsal ones. Lateral lappets and dorsal projection ill-defined, only slightly developed on TC2 (Fig. 20A). Geniculate chaetae acutely bent (Fig. 21A, B) and with very low capitium. Ciliated papilla dorsal to thoracic notopodia not observed. Thoracic uncini in one row with rostrum/capitium length ratio of approximately 2 : 1, and capitium with a first row of four or five (sometimes six) large-sized teeth, followed by several progressively smaller teeth (Fig. 21C–E). Abdomen with 24–35 uncinigers with type 2 uncini (Figs 21F, 28C).

###### Nucleotide diagnostic features.

All sequences of *T. ronningae* sp. nov. share the unique apomorphic nucleotides in positions 129 (G), 399 (G) and 435 (G).

###### Type locality.

Hordaland, Lysefjord (Norway); 25–47 m deep (Figs 10, 18B).

###### Distribution and bathymetry.

Norwegian coast and shelf, Skagerrak; 25–188 m deep (Nygren et al. 2018) (Figs 9, 18B; Suppl. material 1: Table S1).

###### Etymology.

This species is named after Dr. Ann-Helén Rønning, Head Engineer of the Department of Technical and Scientific Conservation, Natural History Museum–NHMO (Oslo), for her help and friendship.

###### Remarks.

*Terebellides ronningae* sp. nov. is characterised by the lack of ciliated papilla dorsal to thoracic notopodia and the presence of papillary projections pointing over the edge of the dorsal anterior border of branchial lamellae, thoracic uncini of type 1 and abdominal of type 2 (Table 1). It is distinguished from the closest relatives of subgroup A2 by the presence of thoracic uncini type 1 instead of type 3 (Table 1).

Specimens examined with SEM bear thoracic uncini with rostrum bendings (Fig. 21C) similar to those of other NEA species (see Discussion for *T. stroemii*). The branchial ventral lobes show variability in their arrangement that is similar to that of *T. europaea*.

Twelve sequences (see Suppl. material 2: Table S2), in ten haplotypes, have been attributed to this species (Nygren et al. 2018). They show 0–0.6% intraspecific divergence, and a minimum of 8.8% uncorrected genetic distance, its closest relative being *T. europaea* (Fig. 2).

##### Terebellides norvegica sp. nov.

Taxon classificationAnimaliaTerebellidaTrichobranchidae

ABF9AE55-A322-51CB-919D-97ECEDB5DCC7

http://zoobank.org/659C513E-01DD-43A0-AC29-D1A744EDA9B0

Figs 1 , 2 , 3G , 9 , 10 , 14C–D , 17E , 18C , 19C , 22 , 23 ; Table 1 ; Suppl. material 1 : Table S1; Suppl. material 2: Table S2

Species 8 – Nygren et al. 2018: 18–22, figs 5, 6, 10, Suppl. material 1: Table S1.

###### Material examined.

**Type material. *Holotype***: ZMBN116378. ***Paratypes*** (36 specs): Barents Sea (ZMBN11636, ZMBN116365, ZMBN116366, ZMBN116367); Norwegian coast (GNM146323, NTNU-VM61388, NTNU-VM61389, NTNU-VM61390, NTNU-VM66569, NTNU-VM66573, NTNU-VM66574, NTNU-VM68197, NTNU-VM68198, ZMBN116362, ZMBN116363, ZMBN116368, ZMBN116369, ZMBN116370, ZMBN116371, ZMBN116372, ZMBN116373, ZMBN116374, ZMBN116375, ZMBN116376, ZMBN116377, ZMBN116379, ZMBN116380, ZMBN116381, ZMBN116382, ZMBN116383, ZMBN116384); Skagerrak (GNM14637, GNM15131, GNM15232, GNM15134, ZMBN116361).

***Holotype*.** Complete specimen, 19.0 mm long and 1.5 mm wide (Figs 3G, 19C); female with oocytes in body cavity.

###### GenBank accession numbers of material examined (COI).

Holotype: MG025148. Paratypes: MG025119, MG025120, MG025122, MG025124, MG025126, MG025127, MG025128, MG025129, MG025131, MG025132, MG025134, MG025135, MG025136, MG025137, MG025138, MG025139, MG025140, MG025141, MG025142, MG025143, MG025144, MG025145, MG025146, MG025147, MG025149, MG025151, MG025152, MG025153, MG025154, MG025155, MG025156. Additional material: MG025117, MG025118, MG025121, MG025123, MG025125, MG025130, MG025133, MG025150.

###### Diagnostic features of type material.

**Figure 22.**
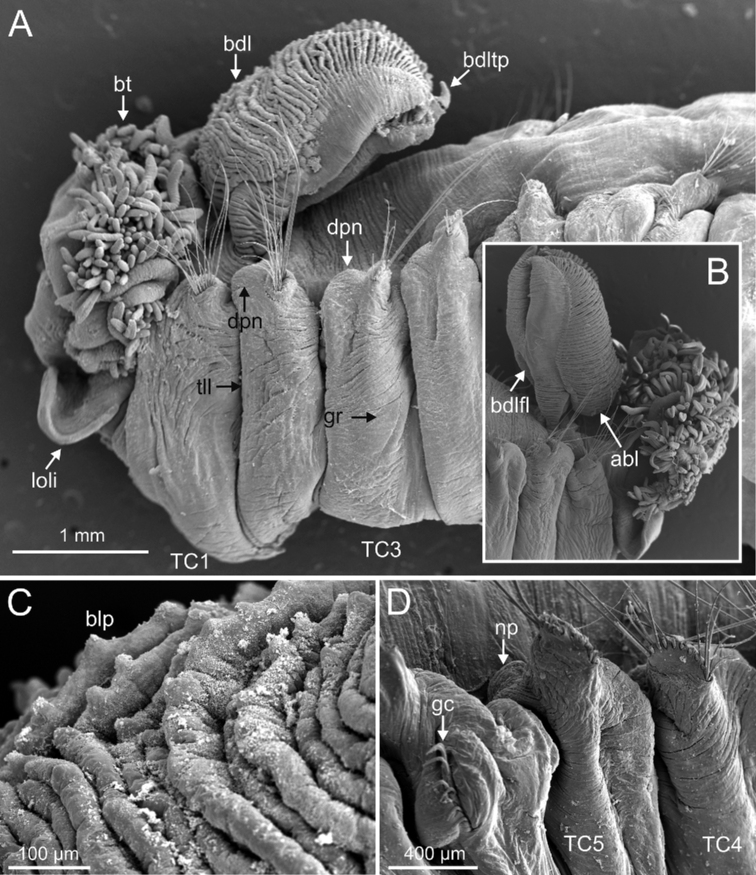
*Terebellides norvegica* sp. nov. (species 8; paratypes, GNM15130 and GNM15134), SEM micrographs. **A** anterior end, left lateral view **B** branchial lobes, ventral view **C** anterior dorsal branchial lamellae and papillae **D** TC4 to TC6, lateral view. Abbreviations: abl – anterior branchial lobe; bdl – branchial dorsal lobe; bdlfl – branchial dorsal lobes fusion line; bdltp – branchial dorsal lobe terminal papilla; blp – branchial lamellae papillae; bt – buccal tentacles; dpn – dorsal projection of notopodium; gc – geniculate chaetae; gr – glandular region; loli – lower lip; np – nephridial papilla; TC – thoracic chaetiger; tll – thoracic lateral lappets.

**Figure 23.**
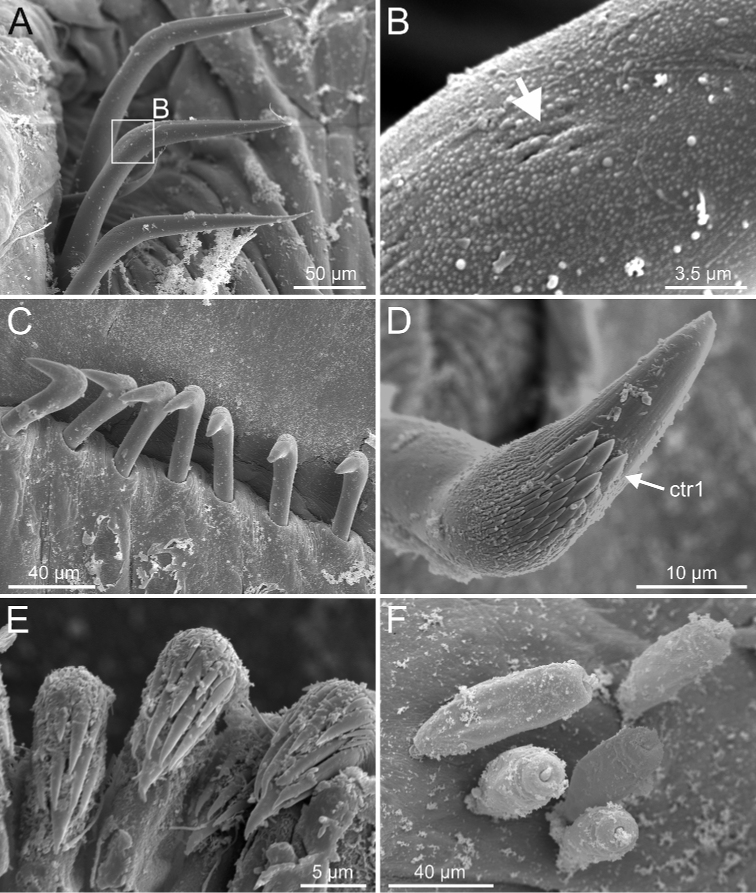
*Terebellides norvegica* sp. nov. (species 8; paratypes, GNM15130 and GNM15134), SEM micrographs. **A** TC6 (TU1), geniculate chaetae **B** detail of geniculate chaeta, arrow pointing to capitium (framed in **A**) **C** simple row of uncini **D** thoracic uncinus, capitium **E** abdominal uncini **F** ciliate epibionts. Abbreviations: ctr1 – first row of capitium teeth.

Complete individuals ranging from 20.0–50.0 mm in length and 1.2–5.0 mm in width (Fig. 17E). Branchial dorsal lobes lamellae with well-developed anterior papillary projections (Fig. 22C). Ventral branchial lobes hidden (Figs 19C, 22A, B) or not (Fig. 3G) by dorsal ones. Lateral lappets and dorsal projection low marked, only partially present on TC2 (Fig. 22A, D). Geniculate chaetae acutely bent, with poorly marked capitium (Fig. 23A, B). Ciliated papilla dorsal to thoracic notopodia not observed. Thoracic uncini in one row (Fig. 23C) with rostrum/capitium length ratio of approximately 2 : 1 and capitium with a first row of two or three medium-sized teeth, followed by several progressively smaller teeth (Fig. 23D). Abdomen with 29–38 chaetigers with type 2 uncini (Fig. 23E). Epibiont ciliates observed in some specimens (Fig. 23F).

###### Nucleotide diagnostic features.

All sequences of *T. norvegica* sp. nov. share the unique apomorphic nucleotides in positions 48 (C) and 285 (G) of the alignement.

###### Typ﻿e locality.

Rogaland (Norway); at depths of between 226 and 242 m (Fig. 18C).

###### Distribution and bathymetry.

Barents Sea, Norwegian coast, Skagerrak; 190–1,268 m deep (Nygren et al. 2018) (Figs 9, 18C; Suppl. material 1: Table S1).

###### Etymology.

The name of the new species refers to the country where members of this lineage were found, along the Norwegian coast from the Barents Sea to the Skagerrak Strait.

###### Remarks.

*Terebellides norvegica* sp. nov. is characterised by the presence of marginal papillae in the anterior region of branchial dorsal lamellae, thoracic uncini of type 3 and abdominal uncini of type 2, and by lacking ciliated papilla dorsal to thoracic notopodia (Table 1). These features are shared with species of subgroup A2: *T. europaea*, *T. ronningae* sp. nov. and *T. scotica* sp. nov. (Table 1), apart from the thoracic uncini type that is different in *T. ronningae* sp. nov. Furthermore, *T. norvegica* sp. nov., *T. europaea* and *T. scotica* sp. nov. also show the same variability in whether ventral branchial lobes are hidden or not by dorsal lobes. Therefore, it seems that members of these three species can only be distinguished according to the DNA sequences. However, they show little overlapping in their geographic distribution and bathymetric ranges (Figs 9, 18A, C, D). *Terebellides norvegica* sp. nov. inhabits deep-water habitats (mostly below 200 m) along the Norwegian coast; its distribution only overlaps with that of *T. europaea* in southern waters (Skagerrak). As stated before, *T. europaea* has a broader distribution reaching to the South NW Iberian Peninsula and is generally found in shallower habitats (<100 m) similarly to *T. scotica* sp. nov. Ciliate epibionts attached over dorsal body surface were also observed (Fig. 23F).

On the other hand, the internal anatomy of *T. norvegica* sp. nov. has been examined by transparency in one alive specimen (Fig. 14D). The digestive tract is divided in an oesophagus clearly distinguishable between TC1 and TC3, that is followed by the stomach and the associated digestive gland (TC4–TC7) and then by the intestine (from TC11). Regarding the circulatory system, a double dorsal blood vessel is present in anterior body end from which arise four afferent vessels at the level of branchial stem and into the branchiae; the coelomic cavity bears oocytes from TC11. All these internal features agree with those described by Jouin-Toulmond and Hourdez (2006) and Parapar and Hutchings (2014) for other species of the genus.

Forty sequences (see Suppl. material 2: Table S2), in 33 haplotypes, have been attributed to this species (Nygren et al. 2018). They show 0–3.1% intraspecific divergence, larger than in other *Terebellides* species, and a minimum of 10.5% uncorrected genetic distance, with its closest relative being *T. scotica* sp. nov. (Fig. 1).

##### Terebellides scotica sp. nov.

Taxon classificationAnimaliaTerebellidaTrichobranchidae

B6E8A2A7-543E-58DD-9CAB-A1FC63969512

http://zoobank.org/74511F62-C57D-4BF7-8B63-48997EB1C8E9

Figs 1 , 2 , 3H , 9 , 17F , 18D , 19D , 24 , 25 ; Table 1 ; Suppl. material 1 : Table S1; Suppl. material 2: Table S2

Species 9 – Nygren et al. 2018: 18–22, figs 5, 6, 10, Suppl. material 1: Table S1.

###### Material examined.

**Type material. *Holotype***: ZMBN116385. ***Paratypes*** (3 specs), North Sea (ZMBN 116382, ZMBN 116386, ZMBN 116387).

***Holotype***. Complete specimen, 45.0 mm long and 4.5 mm width (Fig. 3H, 19D).

###### Additional material.

SMA_BR_23 (GenBank number: MN207187) and SMA_BR_33 (GenBank number: MN207188) of *Terebellides* sp. in Lavesque et al. (2019) (Suppl. material 1: Table S1).

###### GenBank accession numbers of material examined (COI).

***Holotype***: MG025157. ***Paratype***: MG025158.

###### Diagnostic features of type material.

**Figure 24.**
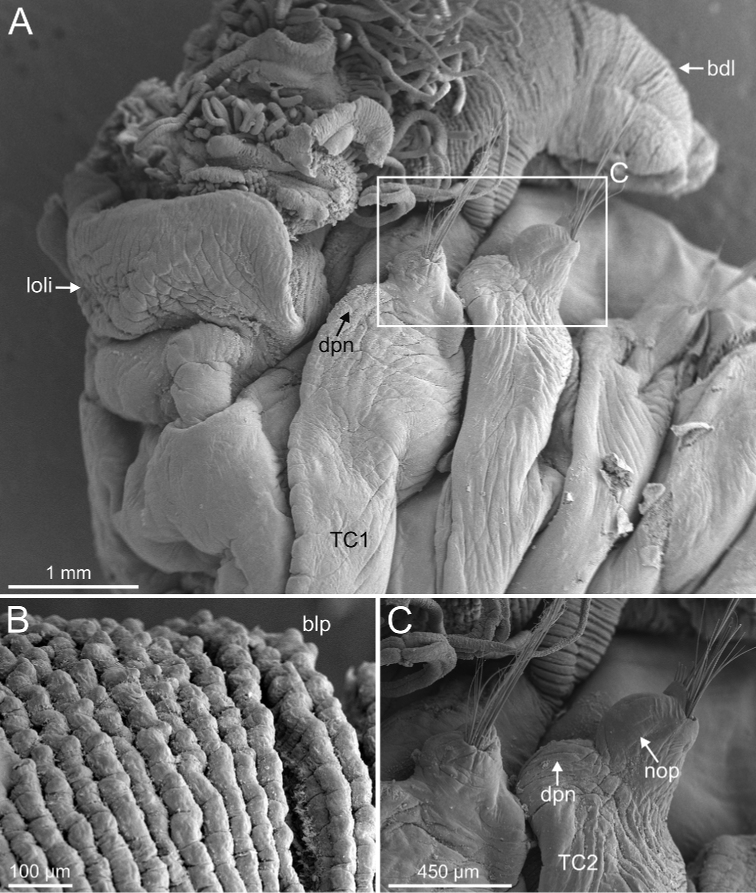
*Terebellides scotica* sp. nov. (species 9; paratype, ZMBN 1163887), SEM micrographs. **A** anterior end, left lateral view **B** anterior dorsal branchial lamellae and papillae **C** TC1 and TC2, lateral view (framed in **A**). Abbreviations: bdl – branchial dorsal lobe; blp – branchial lamellae papillae; dpn – dorsal projection of notopodium; loli – lower lip; nop – notopodial protuberance; TC – thoracic chaetiger.

**Figure 25.**
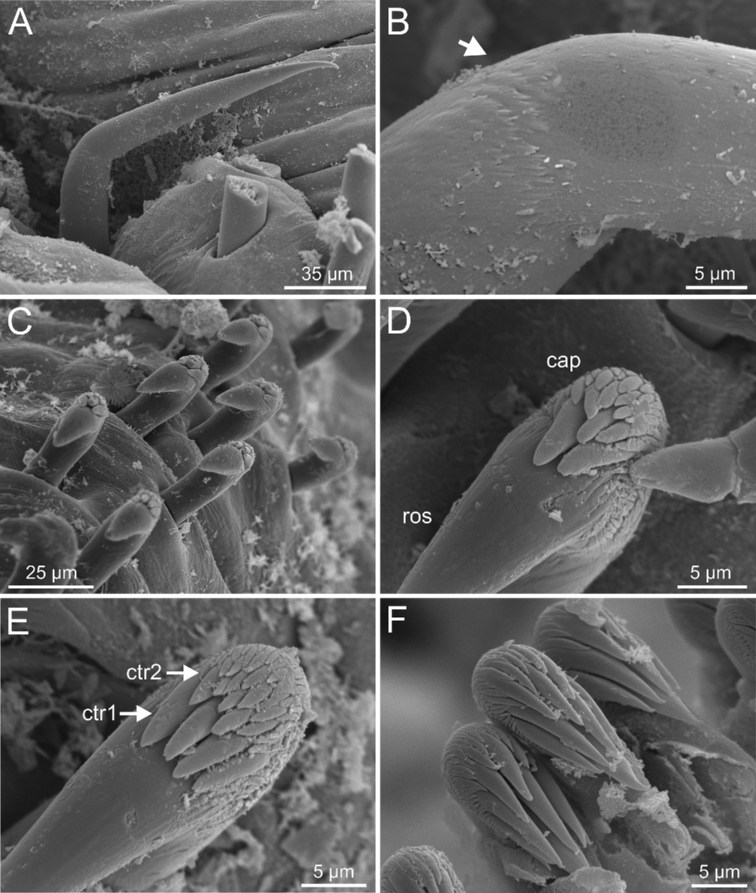
*Terebellides scotica* sp. nov. (species 9; paratype, ZMBN 1163887), SEM micrographs. **A** TC6 (TU1), geniculate chaetae **B** detail of geniculate chaeta (arrow pointing to capitium) **C** double row of thoracic uncini **D, E** thoracic uncini, capitium **F** abdominal uncini. Abbreviations: cap – capitium; ctr1 – first row of capitium teeth; ros – rostrum.

Complete individuals ranging from 6.0–45.0 mm in length and 1.0–4.0 mm in width (Figs 9, 17F). Branchial dorsal lobes lamellae provided with low anterior papillary projections (Fig. 24B). Ventral branchial lobes hidden (Fig. 24A) or not (Figs 3H, 19D) by dorsal ones. Lateral lappets and dorsal projection low marked being only discernible on TC1–3 (Fig. 24A). Geniculate chaetae acutely bent and provided with hardly distinguishable capitium (Fig. 25A, B). Ciliated papilla dorsal to thoracic notopodia not observed. Thoracic uncini in one or two rows (Fig. 25C) with rostrum/capitium length ratio of approximately 2 : 1, and capitium with a first row of 2–4 medium-sized teeth, followed by several progressively smaller teeth (Fig. 25D, E). Abdomen with 18–33 uncinigers provided with type 2 uncini (Fig. 25F).

###### Nucleotide diagnostic features.

There are no unique apomorphic nucleotides in the fragments of COI analysed for *T. scotica* sp. nov., when considering all *Terebellides* species present in the NEA (Suppl. material 2: Table S2). However, when comparing homologous nucleotide positions with members of only Group A (192 sequences in the COI alignment), the following autapomorphies arise: 279 (G), 444 (C), 517 (A), 630 (C).

###### Type locality.

East Orkney Island; 85 m deep (Fig. 18D).

###### Distribution and bathymetry.

North Sea; 48–111 m deep (Nygren et al. 2018) (Fig. 18D; Suppl. material 1: Table S1). Two specimens (*Terebellides* sp. in Lavesque et al. 2019) were identified as *T. scotica* sp. nov. according to molecular sequences; Bay of Brest (France), in rhodolith beds, 5 m deep.

###### Etymology.

This new species is named after Scotland, since its type locality is in the Scottish Orkneys Islands.

###### Remarks.

Among A2 species, *T. scotica* sp. nov., *T. europaea* and *T. norvegica* sp. nov. have thoracic uncini of type 3 and show ventral branchial lobes that may be hidden in between dorsal lobes in some specimens. As stated previously, these species can only be distinguished according to DNA sequences.

The specimen studied under SEM shows a small knob near the notopodial lobe of TC1 (nop, Fig. 24C); its biological role is unknown and it may correspond to an artefact.

Two different sequences (see Suppl. material 2: Table S2; 0.2% distance) have been attributed to this species (Nygren et al. 2018). As stated above, the closest NEA congener is *T. norvegica* sp. nov., at 10.5% genetic distance.

### Subgroup A3

Analyses of molecular data recovered a strongly supported subgroup A3 (Figs 1, 2; Nygren et al. 2018). This group is composed by species 20 + 28 (= *T. bigeniculatus*), and species 21; the latter will be described elsewhere (Gaeva and Jirkov, pers. comm.) but some comments are also provided here (*Terebellides* sp. 2 hereafter).

#### Character/s present only in subgroup A3

**Figure 26.**
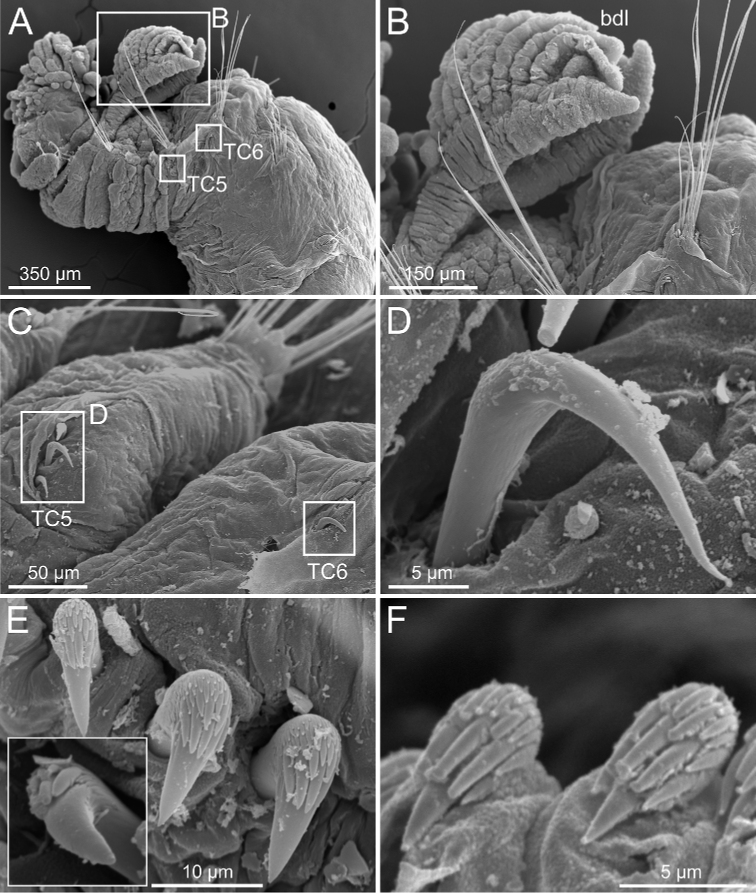
*Terebellides bigeniculatus* Parapar, Moreira & Helgason, 2011 (species 20 + 28; non-type specimens, ZMBN 116512 and ZMBM 116513), SEM micrographs. **A** anterior end, left lateral view **B** branchiae, ventral view **C** TC5 and TC6 (framed: geniculate chaetae location) **D** geniculate chaeta (framed in **C**) **E** thoracic uncini (framed: uncinus rostrum with curved distal end) **F** abdominal uncini. Abbreviations: bdl – branchial dorsal lobe; TC – thoracic chaetiger.

- Branchiae stroemii-type but irregular in many specimens, with all four lobes slightly fused; ventral lobes shorter and slimmer than dorsal ones and not hidden in between.
- First thoracic neuropodia on TC5; several sharply bent, acute-tipped geniculate chaetae present in two chaetigers (TC5 and TC6) (Fig. 26C).

#### Character/s shared with subgroup A1

**Figure 27.**
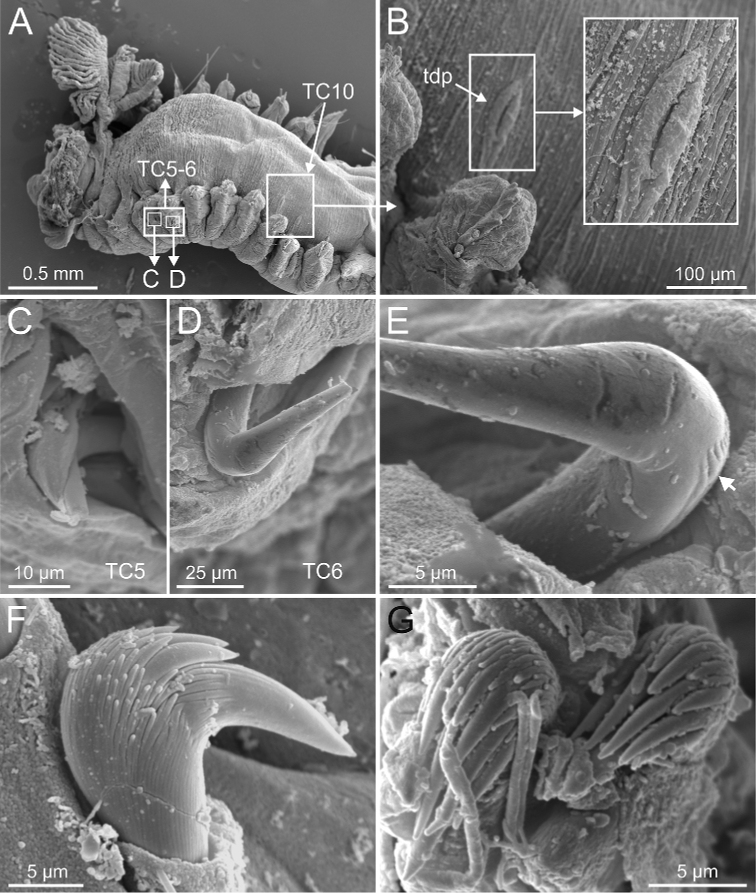
*Terebellides* sp. 2 (species 21; ZMBN 116481 and ZMBN 116486), SEM micrographs. **A** anterior end, left lateral view **B** TC10, thoracic dorsal papilla (framed in **A**) **C, D** geniculate chaetae of TC5 and TC6 respectively (framed in **A**) **E** TC6, geniculate chaeta (arrow pointing to capitium teeth) **F** thoracic uncinus **G** abdominal uncini. Abbreviations: TC – thoracic chaetiger; tdp – thoracic dorsal papilla.

- Border of anterior region of dorsal branchial lamellae not provided with papillary projections.
- Ciliated papilla present, dorsal to thoracic notopodia (Fig. 27B).
- Thoracic uncini type 3 (Fig. 26E).

#### Character/s shared with subgroup A2

- None (Table 1).

#### Character/s variable within subgroup A3

- None (Table 1).

##### Terebellides bigeniculatus

Taxon classificationAnimaliaTerebellidaTrichobranchidae

Parapar, Moreira & Helgason, 2011

29269F26-E684-5C28-AE30-502E279F526E

Figs 1 , 2 , 3D , 4D , 8D , 9 , 10 , 26 , 28E ; Table 1 ; Suppl. material 1 : Table S1; Suppl. material 2: Table S2

Terebellides bigeniculatus Parapar, Moreira & Helgason, 2011: 6–10, figs 1b, 4–7.
Species 20 + 28 Nygren et al. 2018: 18–22, figs 6, 10.

###### Type locality.

Off North West Iceland; 333 m deep (Parapar et al. 2011).

###### Material examined.

6 specimens: Barents Sea (ZMBN 116511); Norwegian coast and shelf (ZMBN 116417, ZMBN 116510, ZMBN 116512, ZMBN 116513, ZMBN 116514).

###### Additional material.

*T. bigeniculatus*: ***Holotype*** (IIH 24923) and 5 ***paratypes*** (IINH 24925) (Suppl. material 1: Table S1).

###### GenBank accession numbers of material examined (COI).

MG025318, MG025319, MG025351, MG025352, MG025353, MG025354, MG025355.

###### Diagnostic features of studied material.

**Figure 28.**
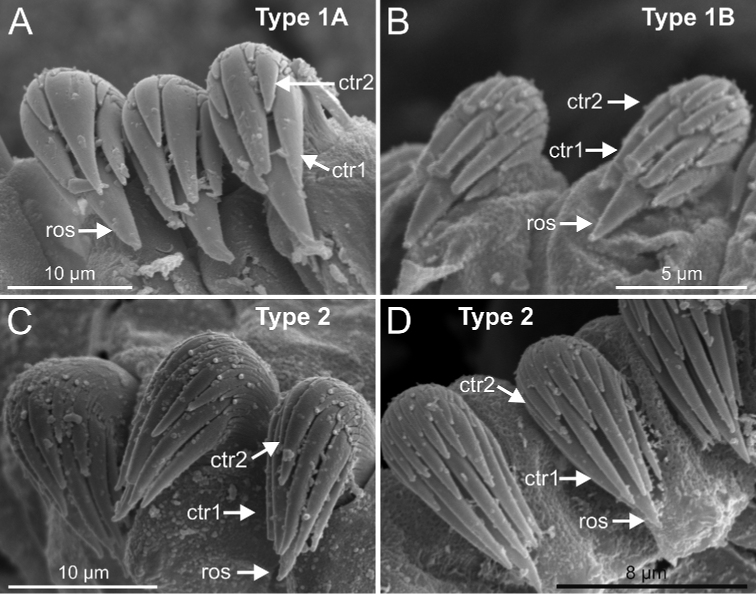
SEM micrographs of abdominal uncini types of *Terebellides* species. **A***T. kongsrudi* sp. nov. (species 13; ZMBN-116409) **B***T. bigeniculatus* Parapar, Moreira & Helgason, 2011 (species 20 + 28; ZMBN-116513) **C***T. ronningae* sp. nov. (species 7; ZMBN-116353) **D***Terebellides stroemii* Sars, 1835 (species 11; ZMBN-116399). Abbreviations: ctr – capitium teeth row; ros – rostrum.

Complete individuals ranging from 10.0–24.0 mm in length. Branchiae clearly fitting with type 1 only in some specimens, irregular in others; dorsal lobes lamellae not provided with papillary projections. Lateral lappets from TC1-TC5 and well-marked dorsal projection of notopodia in TC3 (Figs 3D, 4D). Geniculate chaetae present in TC5 and TC6 (Fig. 26C), acutely bent and provided with hardly distinguishable capitium (Fig. 26D). Ciliated papilla dorsal to thoracic notopodia. Thoracic uncini of type 3, with rostrum/capitium length ratio of approximately 2 : 1 (Fig. 26E), and capitium with a first row of four medium-sized teeth, followed by several progressively smaller teeth. Abdomen with 20–25 chaetigers provided with type 1 uncini (Figs 26F, 28B).

Material examined herein corresponds to a few small and incomplete specimens. Therefore, the list of diagnostic characters given was developed with the aid of the type specimens re-examined and the original description.

###### Nucleotide diagnostic features.

All sequences of *T. bigeniculatus* share the unique apomorphic nucleotides in positions 67 (G) and 138 (G) of the alignement.

###### Distribution and bathymetry.

Around Iceland at both sides of the GIF Ridge; 179–968 m deep (Parapar et al. 2011). Material examined here also confirms its presence in shallow and deep bottoms of Norway and Barents Sea (Fig. 8D).

###### Remarks.

In some of the species delimitation analyses performed, Nygren et al. (2018) were able to distinguish between two closely related lineages, clades 20 and 28, but some analyses of nuclear and mitochondrial datasets lump them together in a single entity. Given that all specimens examined share characteristic features that are distinct from other *Terebellides* species studied herein, clades 20 and 28 have been considered in the present study as a single species and identified as *T. bigeniculatus*.

As stated above, the sequenced specimens are small and not well preserved, hindering the examination of relevant morphological features with taxonomic value (i.e., branchial type). However, this species is characterised by having geniculate chaetae on TC5 and TC6 instead of only on one chaetiger (Parapar et al. 2011: 7) as in congeners listed in the Key of the present study. Furthermore, *T. bigeniculatus* is characterised by the low fusion of the usually irregularly-shaped branchial lobes (Parapar et al. 2011: 7–8, figs 4, 5a, b), ventral lobes are not obscured by dorsal ones, the lack of marginal papillae in the anterior region of the branchial dorsal lamellae, the presence of ciliated papilla dorsal to thoracic notopodia, and by having thoracic uncini of type 3 and abdominal uncini of type 1. However, it is likely that the irregular shape of the branchiae may correspond to an artefact related to fixation/preservation; other specimens show instead well-defined branchiae that agree with those of A1 and A2 species but less developed (Fig. 26A, B; Parapar et al. 2011: 8, fig. 5a). Regarding the four branchial types as defined by Parapar et al. (2016c), branchiae of *T. bigeniculatus* might correspond therefore to type 3 but with lobes showing a more variable shape.

The original description states that nephridial papillae are located on TC3–TC4 or TC4–TC5 (Suppl. material 1: Table S1; Parapar et al. 2011: 7–9, figs 5c, 6d). Examination of the holotype and several paratypes confirmed that pores are on TC4 and TC5, as in other Group A species. Nephridial pores, as found in most *Terebellides* species, are usually flat and can be easily overlooked when examined with STM and even SEM; those of *T. bigeniculatus* are larger and easier to distinguish comparatively with STM (Parapar et al. 2011: 9, fig. 6d).

Members of species 21 (see below, as *Terebellides* sp. 2) also bear geniculate chaetae in two chaetigers; this feature had been considered as unique to *T. bigeniculatus* regarding other NEA species. However, species 21 is present in Arctic waters (cf. Nygren et al. 2018: fig. 6) while the distribution of members of species 20 + 28 and identified here as *T. bigeniculatus* agrees with that of the type specimens (see Fig. 8D).

##### Terebellides

Taxon classificationAnimaliaTerebellidaTrichobranchidae

sp. 2

9FDD6827-E227-5DDE-8EA6-8CBEAE070DE2

Figs 1 , 2 , 9 , 27 ; Table 1 ; Suppl. material 1 : Table S1; Suppl. material 2: Table S2

Species 21 Nygren et al. 2018: 18–22, figs 5, 6, 10.

###### Material examined.

4 specimens: **Barents Sea**. ZMBN 116481; ZMBN 116486.

###### Remarks.

As explained for *Terebellides* sp. 1, two specimens were examined under SEM; these share with *T. bigeniculatus* the irregular shape of branchial lobes (Fig. 27A), the presence of geniculate chaetae on TC5 and TC6 (Fig. 27C–E) and abdominal uncini of type 1B (Fig. 27G). They share with subgroup A1 the presence of one ciliated papilla dorsal to thoracic notopodium (Fig. 27B) and thoracic uncini of type 3 (Fig. 27F).

On the other hand, species 18 and 19 of A1 (not described here because of the few specimens being available) and 23 (A4) have a geographic distribution similar to that of *T. bigeniculatus* but their position in the cladogram by Nygren et al. (2018: fig. 5) suggests that they may not bear geniculate chaetae in two chaetigers.

There are no unique diagnostic nucleotide positions that are shared by the two haplotypes (in 18 sequences) in COI. Eighteen sequences, in one single haplotype, have been attributed to this species (Nygren et al. 2018). Members of this species show a minimum of 3.0% uncorrected genetic distance, with its closest relative being *T. bigeniculatus* (Fig. 1).

##### Key to European species of *Terebellides*

The following key of European *Terebellides* species is based on Lavesque et al. (2019) and updated by including all species of Group A (in bold) apart from those that will be described elsewhere. The known geographic or bathymetric distribution has been used when there is a lack of discriminatory morphological characters between some species (e.g., subgroup A2).

**Table:** 

1	Geniculate chaetae on TC5 and TC6^1^	**(subgroup A3) *T. bigeniculatus* Parapar, Moreira & Helgason, 2011**
–	Geniculate chaetae on TC6 only	**2**
2	Branchial lamellae margins lacking papillae^2^	**3**
–	Branchial lamellae margins with papillae	**11**
3	Lower branchial lobes with long posterior projections as filaments	**4**
–	Lower branchial lobes with short posterior projections	**5**
4	Glandular region on TC3 present; branchial lamellae pointed; notochaetae from TC1 longer than following ones; dorsal papillae absent	***T. parapari* Lavesque, Hutchings, Daffe, Nygren & Londoño-Mesa, 2019**
–	Glandular region on TC3 absent; branchial lamellae rounded; all notochaetae equal-sized; dorsal papillae present	***T. shetlandica* Parapar, Moreira & O’Reilly, 2016**
5	Ventral white band present on TC4 after MG staining	**6**
–	No distinct pattern on TC4 after MG staining	**7**
6	Large species (>30 mm in length); 5^th^ branchial lobe present; notochaetae of TC1 similar to following ones; main fang of thoracic uncini straight	***T. gracilis* Malm, 1874**
–	Small species (<20 mm in length); 5^th^ branchial lobe absent; notochaetae of TC1 absent or shorter than following ones; main fang of thoracic uncini ‘eagle head’-shaped	***T. ceneresi* Lavesque, Hutchings, Daffe, Nygren & Londoño-Mesa, 2019**
7	First notopodia and notochaetae longer than following ones	***T. mediterranea* Parapar, Mikac & Fiege, 2013**
–	First notopodia and notochaetae similar or shorter than following ones	**8**
8	Large-sized species (>50 mm); dorsal rounded projections on TC1–TC5 conspicuous	**(subgroup A1) 9**
–	Small-sized species (<20 mm); dorsal rounded projections on TC1–TC5 absent; main fang of thoracic uncini straight	**10**
9	Abdominal uncini type 1^3^	***T. kongsrudi* sp. nov.** and ***T. bakkeni* sp. nov.**
–	Abdominal uncini type 2^3^	***T. stroemii* Sars, 1835**
10	5^th^ branchial lobe absent	***T. atlantis* Williams, 1984**
–	5^th^ lobe present	***T. gralli* Lavesque, Hutchings, Daffe, Nygren & Londoño-Mesa, 2019**
11	Glandular region on TC3 round or oval	**12**
–	Glandular region on TC3 otherwise	**13**
12	Glandular region on TC3 stained white; branchial lamellae with rounded papillae; TC1–3 without conspicuous dorsal projection	***T. lilasae* Lavesque, Hutchings, Daffe, Nygren & Londoño-Mesa, 2019**
–	Glandular region on TC3 stained blue; branchial lamellae with conical papillae; TC1–3 with conspicuous dorsal projection	***T. bonifi* Lavesque, Hutchings, Daffe, Nygren & Londoño-Mesa, 2019**
13	Most branchial lamellae with marginal papillae; upper lip elongated	***T. resomari* Lavesque, Hutchings, Daffe, Nygren & Londoño-Mesa, 2019**
–	Only anterior branchial lamellae with marginal papillae; upper lip not elongated	**(subgroup A2) 14**
14	Thoracic uncini type 1^4^	***T. ronningae* sp. nov.**
–	Thoracic uncini type 3^4^	**15**
15	Deep-water species; mostly below 200 m deep	***T. norvegica* sp. nov.**
–	Shallow-water species; mostly above 100 m deep	**16**
16	Present from Southern Norway to NW Iberian Peninsula	***T. europaea* Lavesque, Hutchings, Daffe, Nygren & Londoño-Mesa, 2019**
–	Present in the Shetland and Orkneys Islands and in Brittany	***T. scotica* sp. nov.**

## Discussion

### Group A species: taxonomy and distribution

The comprehensive study by Nygren et al. (2018) revealed that the genus *Terebellides* holds a large species diversity in NEA waters regardless its morphological homogeneity. Over 25 molecular entities that meet the requirements to be recognized as species were recovered forming four main and robust clades (A–D); Group A is composed, in turn, by thirteen species. Among the latter, members of only three species were identified herein as current nominal species: *T. stroemii*, *T. bigeniculatus*, and *T. europaea*; the remaining ten represent undescribed taxa.

Within Group A, three subgroups (A1–A3) can be defined based on molecular data, being only A2 and A3 well supported and congruent among all molecular analyses and datasets (Figs 1, 2; Nygren et al. 2018) but also by morphological features. A1 and A2 gather species morphologically similar to *T. stroemii*, while species included in subgroup A3 share morphological features with *T. bigeniculatus*. The original description of *T. stroemii* by Sars (1835) lacks detailed specific diagnostic features as are recognised nowadays in many closely related species, most of them described in the last years. On the contrary, *T. bigeniculatus* belongs to a small group of species bearing geniculate chaetae in two thoracic chaetigers (TC5 and TC6) instead of one (TC6), a distinct morphological trait for the group; *T. bigeniculatus* was described from deep Icelandic waters by Parapar et al. (2011), and only later reported NEA by Nygren et al. (2018). *Terebellides europaea* was recently described after molecular analyses by Lavesque et al. (2019) and fits within species of A1+A2. Other species from NEA, namely *T. gracilis*, *T. atlantis*, *T. williamsae*, *T. irinae* and *T. shetlandica* Parapar, Moreira & O’Reilly, 2016, differ from members of Group A in shape and body length, ventral colouration in a number of thoracic chaetigers, branchiae shape and degree of fusion and relative size of dorsal/ventral lobes (see Holthe 1986; Jirkov 2001; Parapar et al. 2011, 2016c). The aforementioned species fit either within groups B, C, or D sensu Nygren et al. (2018) and will be dealt with in a forthcoming paper.

The characters considered to delineate morphologically the aforementioned subgroups (A1–A3) should be taken with care because there are limitations due to number of specimens available to be studied and their condition of preservation. However, considering the variety and origin of the material examined we were able to elucidate some general patterns on taxonomy and distribution of the studied species. Thus, all studied species seem quite homogeneous in terms of general body features and share many characters; however, presence/absence of some macroscopic/microscopic characters has allowed their organization in the subgroups proposed above. Nevertheless, some species could not be differentiated according to morphological characters but genetic data. On the other hand, geographic distributions of species do not show apparent gaps; some species have a wider distribution and were more frequent in samples such as *T. norvegica* sp. nov. and *T. kongsrudi* sp. nov.; this suggests that many previous reports of *T. stroemii* in NEA might correspond to the aforementioned species. Other species apparently show a more restricted distribution, i.e., *T. bakkeni* sp. nov. in northern Norway or have their limit of distribution in southern Norway, as *T. europaea*. Similarly, there are no gaps in the bathymetric distribution of species, but some seem to appear typically at shallow depths, reaching the continental shelf (0–200 m) such as *T. europaea*, *T. ronningae* sp. nov. and *T. scotica* sp. nov. On the contrary, *T. bigeniculatus* and *T. norvegica* sp. nov. are found at depths of below 200 m while *T. stroemii*, *T. bakkeni* sp. nov. and *T. kongsrudi* sp. nov. show a wider bathymetric distribution.

Given the morphological homogeneity, DNA sequences have been shown to provide advantageous data and support when it comes to species delineation in *Terebellides*. The most informative markers in previous studies are COI and ITS (Nygren et al. 2018; Lavesque et al. 2019). In the present study, analyses have been mainly based on mitochondrial COI, the universal barcoding gene, because it offers no ambiguities in the alignment process, and is the most commonly used in molecular taxonomy in annelids (e.g., Borda et al. 2013; Tomioka et al. 2016; Álvarez-Campos et al. 2017; Aguado et al. 2019; Grosse et al. 2020) and other taxa (e.g., Kekkonen and Hebert 2014). After species delimitation, identification to the correct nominal species level is ideal, as species names allow the communication, study, quantification, classification, use and management of life on the planet. This has been the motivation of recognising unequivocal diagnostic nucleotides in specific positions for the species described in the present study. As with morphological traits, molecular diagnostic characters are tested continuously when additional intraspecific and interspecific variation within the groups has been found. Nevertheless, and as pointed out by previous studies, diagnostic nucleotides may be an effective and relatively simple way for species identification (Rach et al. 2008; Wong et al. 2009).

### Comparisons with other NE Atlantic *Terebellides*

Lavesque et al. (2019) described eight new species of *Terebellides* from continental France considering an integrative taxonomy approach. Those species could be informally grouped in two assemblages:

1. Species similar to Group A sensu Nygren et al. (2018) regarding body colour and shape, and branchiae features: T. bonifi, T. europaea, T. gentili, T. gralli and T. lilasae.
2. Species closer to groups B, C or D sensu Nygren et al. (2018): T. ceneresi, T. parapari and T. resomari.

The first five species were already discussed above. Regarding the remaining three species, only *T. ceneresi* was sequenced by Lavesque et al. (2019) and according to their phylogenetic analyses, it is not related to any species of Group A; in fact, it differs from Group A species: a) in having a very distinct MG staining pattern corresponding to a solid stain manifested in the first ten thoracic chaetigers, being lighter in TC4; b) the anterior branchial lobe (5^th^ lobe) is not present; c) the outer edge of branchial lamellae bears tufts of cilia. These characters would relate *T. ceneresi* to Group D sensu Nygren et al. (2018). This species was described with ‘eagle head’-shaped thoracic uncini, which are similar to those of *T. stroemii*, *T. ronningae* sp. nov. and *T. kongsrudi* sp. nov. as described here and *T. stroemii* sensu Parapar and Hutchings (2014). However, as explained above (see Remarks for *T. stroemii*), the taxonomic value of this character should be viewed cautiously and its consistent presence across the three aforementioned species needs to be assessed.

*Terebellides parapari* differs from Group A species in the shape and arrangement of branchial lobes that are free from each other, and by the presence of terminal filament in ventral lobes. These features and its short body length relate *T. parapari* to *T. shetlandica* and Group B sensu Nygren et al. (2018). Finally, *T. resomari* is unique among NEA*Terebellides* because of having “not well packed (separated) disposition of the branchial lamellae” (Lavesque et al. 2019: 177, fig. 18B) and therefore branchiae seem lacking a defined shape. In addition, this species also shows the “upper lip very elongated with convoluted margins” (Lavesque et al. 2019: 177, fig. 18C), that was also reported by Parapar et al. (2020) for *Terebellides* sp. from the Atlantic African coast. Therefore, these unusual features do not allow for the allocation of *T. resomari* to any group as defined by Nygren et al. (2018).

### Discriminant vs. non-discriminant body characters in species delineation

This study has revealed that some of the traditionally morphological-based taxonomic characters are not appropriate for *Terebellides* species identification. The number of species in the genus is now large and their morphological homogeneity high. Regarding Group A, two macroscopic characters have, however, been useful: 1) presence of geniculate chaetae in one or two chaetigers (A1+A2 vs A3), 2) presence of papillary projections in the border of branchial lamellae (A2 vs A1+A3). On the contrary, we found that the development of lateral lappets and the presence of a dorsal projection on the anterior thoracic notopodia seem dependent on size/age and preservation, and therefore these characters should be taken with care for species identification. Similarly, the species in Group A seem quite homogeneous when considering branchial morphology, particularly within A1 and A2. Some of the morphological differences observed between *Terebellides* species rely in the exposure of the ventral lobes (hidden or not behind the dorsal lobes). However, we have also observed some degree of variability between specimens belonging to the same species and could be due to size or the contraction of specimens after fixation.

Morphology of thoracic and abdominal uncini seems useful for species identification; such features need to be examined under SEM and are being considered in descriptions of *Terebellides* in the last years. Recently, Parapar et al. (2020) describe tentatively several types of thoracic uncini. The uncini of the NEA species treated here are quite similar because of their phylogenetic proximity, being *T. ronningae* sp. nov. the only species that differ in uncini type from other congeners of subgroup A2. There were, however, differences in abdominal uncini that correspond to two morphologies that agree well, in turn, with groups of species as defined by molecular-based phylogenetic analyses. Following Parapar et al. (2020), we propose here the use of similar criteria for the characterization of abdominal uncini, that are based on the rostrum vs. capitium length ratio (RvC), and the number of the capitium teeth and their relative size. Therefore, considering our results after SEM examination and other previous work, two main types of abdominal uncini can be defined:

#### Type 1

Capitium of ca. 0.7 of total length of rostrum (RvC = 1/0.7); capitium simple, composed of a few wide denticles, being 3(5) in first row and 1(2) in a second row (Fig. 28A, B). In turn, Type 1A and 1B would differ in number of capitium teeth, being higher in B (Fig. 28A, B, Table 1). This typology is present in *T. bakkeni* sp. nov. (1A), *T. kongsrudi* sp. nov. (1A) and *T. bigeniculatus* (1B; see also Parapar et al. 2011: fig. 7f). Type 1 uncini are apparently also present in *T. gracilis* (sensu Parapar et al. 2011, 2013), *T. narribri* Schüller & Hutchings, 2010, *T. mediterranea* Parapar, Mikac & Fiege, 2013, *T. toliman* Schüller & Hutchings, 2013, *T. ectopium* Zhang & Hutchings, 2018, *T. kirkegaardi* Parapar, Martin & Moreira, 2020 and *T. longiseta* Parapar, Martin & Moreira, 2020 (Parapar et al. 2013, 2020; Schüller and Hutchings 2010, 2013; Zhang and Hutchings 2018).

#### Type 2

Capitium of almost same length as rostrum (RvC = 1/0.9); capitium much complex than in Type 1, composed of a first row of 4(5) denticles and a variable number of teeth in two more rows with decreasing number and size posterior to them (Fig. 28C, D). Present in *T. europaea*, *T. ronningae* sp. nov., *T. norvegica* sp. nov., *T. scotica* sp. nov., and *T. stroemii* (Table 1). Type 2 is apparently also present in *T. kergelensis* McIntosh, 1885 (sensu Parapar and Moreira 2008a), *T. jitu* Schüller & Hutchings, 2010, *T. canopus* Schüller & Hutchings, 2013, *T. persiae* Parapar, Moreira, Gil & Martin, 2016, *T. baliensis* Hsueh & Li, 2017, *T. guangdongensis* Zhang & Hutchings, 2018, *T. augeneri* Parapar, Martin & Moreira, 2020, *T. fauveli* Parapar, Martin & Moreira, 2020, *T. nkossa* Parapar, Martin & Moreira, 2020, and *T. ramili* Parapar, Martin & Moreira, 2020 (Parapar et al. 2016a, 2020; Hsueh and Li 2017; Zhang and Hutchings 2018). This “more complex” type 2 condition of abdominal uncini does not seem related to body size; for instance, small species such as *T. atlantis* sensu Parapar et al. (2011: 5, fig. 3f) and *T. shetlandica* (Parapar et al. 2016c: 218, fig. 6f) are provided with such uncini. The validity of this proposed uncini classification should be assessed across species considering specimens of different sizes and across abdominal chaetigers.

On the other hand, we observed differences in whether the capitium is defined or not in geniculate chaetae of TC5/TC6, as previously highlighted by Parapar et al. (2011, 2013, 2016a, 2016b, 2016c). For instance, *T. ginkgo* Schüller & Hutchings, 2012 shows a well-defined capitium conformed by many large-sized teeth whereas other species bear an almost inconspicuous capitium (e.g., *T. bakkeni* sp. nov., *T. kongsrudi* sp. nov.) (Schüller and Hutchings 2012: 10, fig. 5a–c; Figs 6G, 12G); Parapar et al. (2011) also reported from Iceland several species with conspicuous capitium, i.e., *T. atlantis*, *T. gracilis* and *T. stroemii*. In this sense, the specimens of *T. stroemii* examined here bear a low capitium in comparison to those aforementioned from Iceland (Parapar et al. 2011); this suggests that the latter might not correspond to *T. stroemii* but to other taxa as explained above. Again, the taxonomic value of this character should be tested in other species considering potential intraspecific variation.

### Methyl Green staining pattern

The MG staining pattern was mostly similar across the studied species and according to type 1 sensu Schüller and Hutchings (2010), being solid in three to five anterior chaetigers, TC1–TC3(5), striped in subsequent seven or eight chaetigers, i.e., TC4(6)–TC10(11), and fading towards the end of the thorax at TC18; minor observed differences can be attributed to body size, degree of contraction and preservation of specimens. Parapar et al. (2011) reported a similar pattern for specimens identified as *T. stroemii* from Iceland: solid in the first six chaetigers after turning into a striped pattern and fading in the posterior thoracic segments, while for *T. bigeniculatus* staining is solid from TC1 to TC11, striped between TC12 and TC14, and then fading in the following segments. The first pattern only partially agrees with that of *T. stroemii* (species 11) and the second one would match better with that of *T. bigeniculatus* (species 20 + 28) as examined here. Parapar and Hutchings (2014) reported a MG staining pattern for neotypes of *T. stroemii* being solid from TC1 to TC3, striped from TC4 to TC12 and fading in the last thoracic segments; this is exactly the same pattern as observed in *T. stroemii* from Norway (Suppl. material 1: Table S1).

### Nephridial papillae

Schüller and Hutchings (2010) and Parapar et al. (2011), among others, suggest that position of thoracic papillae (nephridial/genital) should be considered as of taxonomic value. We agree with this and have found that papillae are present always in TC4 and TC5 in the species/clades studied here. This position has also been reported in *T. gracilis* sensu Parapar et al. (2011, 2013), *T. mediterranea*, *T. kerguelensis*, and *T. hutchingsae* Parapar, Moreira & Martin, 2016. On the contrary, other species reported elsewhere have such papillae in TC1 instead, including *T. persiae* Parapar, Moreira, Gil & Martin, 2016, *T. mediterranea*, and *T. hutchingsae*.

## Conclusions

To sum up all results and according to the discussion of the aforementioned characters, the general characteristics for each subgroup of Group A sensu Nygren et al. (2018) are listed below. A1 and A2 are particularly close to each other and were informally designed by Nygren et al. (2018) as “*stroemii*-group”; subgroup A3 is the most dissimilar, with *T. bigeniculatus* as the typical species.

### Subgroup A1

Species are similar morphologically and differ from A2 in lacking papillae on branchial lamellae and in having ciliated papillae on thoracic notopodia. Regarding morphology and distribution, *T. bakkeni* sp. nov. and *T. kongsrudi* sp. nov. are closest to each other than to *T. stroemii*. *Terebellides stroemii* (as species 11 here) shows also a similar geographic and bathymetric distribution (Table 1), but seems less frequent across Norway and differs in abdominal uncini type (cf. Fig. 7G vs. Figs 6G, 12G).

### Subgroup A2

The subgroup is morphologically homogeneous. It differs from A1 in having lamellae papillae and by the lack of thoracic ciliated papillae (at least not observed with SEM). The most recognisable species is *T. ronningae* sp. nov. because of having thoracic uncini of type 1, a long rostrum and a capitium provided with long first row teeth; the other three species bear thoracic uncini of type 3 and differ of each other in the geographic (*T. europaea*, *T. scotica* sp. nov.) and bathymetric distribution (*T. norvegica* sp. nov.).

### Subgroup A3

This subgroup is composed by *T. bigeniculatus* (species 20 + 28) and species 21 (not formally described here). Branchial shape is irregular and geniculate chaetae are present in two thoracic chaetigers (TC5 and TC6). Other features are shared with A1 such as lack of lamellae papillae; thoracic uncini type 3 or presence of thoracic ciliated papillae. The bathymetric distribution of species is similar to A1.

## Supplementary Material

XML Treatment for Terebellides

XML Treatment for Terebellides

XML Treatment for Terebellides bakkeni

XML Treatment for Terebellides stroemii

XML Treatment for Terebellides kongsrudi

XML Treatment for Terebellides

XML Treatment for Terebellides europaea

XML Treatment for Terebellides ronningae

XML Treatment for Terebellides norvegica

XML Treatment for Terebellides scotica

XML Treatment for Terebellides bigeniculatus

XML Treatment for Terebellides

## Footnotes

This character is also present in clade 21, which will be described elsewhere.

This character is also present in clade 12, which will be described elsewhere.

Types of abdominal uncini as described in this work.

Types of thoracic uncini sensu Parapar et al. (2020).

## Supplementary materials

Supplementary material 1 Table S1. Locality and collecting data, museum registration numbers and references to figures of *Terebellides* specimens Data type occurences Explanation note Locality and collecting data, museum registration numbers and references to figures of *Terebellides* specimens described in this work. Country names are transcribed from original museum vials. https://binary.pensoft.net/file/474823This dataset is made available under the Open Database License (http://opendatacommons.org/licenses/odbl/1.0/). The Open Database License (ODbL) is a license agreement intended to allow users to freely share, modify, and use this Dataset while maintaining this same freedom for others, provided that the original source and author(s) are credited.Julio Parapar, María Capa, Arne Nygren, Juan Moreira

Supplementary material 2 Table S2. List of COI sequences considered in present study (Group A), museum vouchers and GenBank accession numbers Data type COI sequences, museum vouchers and GenBank accession numbers Explanation note List of COI sequences considered in present study (Group A), museum vouchers and GenBank accession numbers. Abbreviations of housing institutions: ZMBN = Department of Natural History, University Museum of Bergen; GNM = The Gothenburg Museum of Natural History; NTNU-VM = Norwegian University of Science and Technology, University Museum, Trondheim; SMF = Senckenberg Museum Frankfurt. https://binary.pensoft.net/file/474824This dataset is made available under the Open Database License (http://opendatacommons.org/licenses/odbl/1.0/). The Open Database License (ODbL) is a license agreement intended to allow users to freely share, modify, and use this Dataset while maintaining this same freedom for others, provided that the original source and author(s) are credited.Julio Parapar, María Capa, Arne Nygren, Juan Moreira

## References

[B1] Aguado MT Capa M Lago-Barcia D Gil J Pleijel F Nygren A (2019) Species delimitation in *Amblyosyllis* (Annelida, Syllidae). PLOS One 14(4): e0214211. 10.1371/journal.pone.0214211 PMC6457521 30970025

[B2] Álvarez-Campos P Giribet G Riesgo A (2017) The *Syllis gracilis* species complex: a molecular approach to a difficult taxonomic problem (Annelida, Syllidae). Molecular Phylogenetics and Evolution 109: 138–150. 10.1016/j.ympev.2016.12.036 28043876

[B3] Bakken T Hårsaker K Daverdin M (2020) Marine invertebrate collection NTNU University Museum. Version 1.535. NTNU University Museum. [Occurrence dataset:] 10.15468/ddbs14 [accessed via GBIF.org on 26 June 2020]

[B4] Barraclough TG (2010) Evolving entities: towards a unified framework for understanding diversity at the species and higher levels. Philosophical Transactions of the Royal Society B – Biological Sciences 365(1547): 1801–1813. 10.1098/rstb.2009.0276 PMC2871889 20439282

[B5] Borda E Kudenov JD Chevaldonné P Blake JA Desbruyères D Fabri M-C Hourdez S Pleijel F Shank TM Wilson NG Schulze A Rouse GW (2013) Cryptic species of *Archinome* (Annelida: Amphinomida) from vents and seeps. Proceedings of the Royal Society B – Biological Sciences 28(1770): e20131876. 10.1098/rspb.2013.1876 PMC3779335 24026823

[B6] Gagaev SY (2009) *Terebellides irinae* sp. n., a new species of *Terebellides* (Polychaeta: Terebellidae) from the Arctic basin. Russian Journal of Marine Biology 35: 474–478. 10.1134/S1063074009060042

[B7] Grosse M Bakken T Nygren A Kongsrud JA Capa M (2020) Species delimitation analyses of NE Atlantic *Chaetozone* (Annelida, Cirratulidae) reveals hidden diversity among a common and abundant marine annelid. Molecular Phylogenetics and Evolution 149: e106852. 10.1016/j.ympev.2020.106852 32417496

[B8] Hoang DT Chernomor O Von Haeseler A Minh BQ Vinh LS (2018) UFBoot2: improving the ultrafast bootstrap approximation. Molecular Biology and Evolution 35(2): 518–522. 10.1093/molbev/msx281 29077904 PMC5850222

[B9] Holthe T (1986) PolychaetaTerebellomorpha. Marine Invertebrates of Scandinavia 7: 1–194.

[B10] Hsueh P-W Li K-R (2017) Additions of new species to *Thelepus* (Thelepodidae), with description of a new *Terebellides* (Trichobranchidae) from Taiwan. Zootaxa 4244(3): 429–439. 10.11646/zootaxa.4244.3.10 28610116

[B11] Imajima M Williams SJ (1985) Trichobranchidae (Polychaeta) chiefly from the Sagami and Saruga Bays, collected by R/V Tansei-Maru (Cruises KT-65/76). Bulletin of the National Science Museum of Tokyo 11(1): 7–18.

[B12] Jirkov IA (1989) Bottom fauna of the USSR. Polychaeta. Moscow State University Press, Moscow, 141 pp. [English translation from Russian]

[B13] Jirkov IA (2001) Polychaeta of the Arctic Ocean. Yanus-K, Moskva, 632 pp. [in Russian]

[B14] Jirkov IA Leontovich MK (2013) Identification keys for Terebellomorpha (Polychaeta) of the eastern Atlantic and the North Polar Basin. Invertebrate Zoology 10: 217–243. 10.15298/invertzool.10.2.02

[B15] Jouin-Toulmond C Hourdez S (2006) Morphology, ultrastructure and functional anatomy of the branchial organ of *Terebellides stroemii* (Polychaeta: Trichobranchidae) and remarks on the systematic position of the genus *Terebellides*. Cahiers de Biologie Marine 47: 287–299.

[B16] Katoh K Misawa K Kuma KI Miyata T (2002) MAFFT: a novel method for rapid multple sequence alignment based on fast Fourier transform. Nucleic Acids Research 30(14): 3059–3066. 10.1093/nar/gkf436 12136088 PMC135756

[B17] Kekkonen M Hebert PD (2014) DNA barcode‐based delineation of putative species: efficient start for taxonomic workflows. Molecular Ecology Resources 14(4): 706–715. 10.1111/1755-0998.12233 24479435 PMC4264940

[B18] Lavesque N Hutchings P Daffe G Nygren A Londoño-Mesa MH (2019) A revision of the French Trichobranchidae (Polychaeta), with descriptions of nine new species. Zootaxa 4664(2): 151–190. 10.11646/zootaxa.4664.2.1 31716675

[B19] Malmgren AJ (1866) Nordiska Hafs–Annulater. Öfversigt af Königlich Vetenskapsakademiens förhandligar, Stockholm 22: 51–410.

[B20] McIntosh WC (1885) Report on the AnnelidaPolychaeta collected by H.M.S. Challenger during the years 1873–1876. Challenger Reports 12: 1–554.

[B21] Nguyen LT Schmidt HA Von Haeseler A Minh BQ (2015) IQ-TREE: a fast and effective stochastic algorithm for estimating maximum-likelihood phylogenies. Molecular Biology and Evolution 32(1): 268–274. 10.1093/molbev/msu300 25371430 PMC4271533

[B22] Nygren A Parapar J Pons J Meißner K Bakken T Kongsrud JA Oug E Gaeva D Sikorski A Johansen RA Hutchings PA Lavesque N Capa M (2018) A mega-cryptic species complex hidden among one of the most common annelids in the North East Atlantic. PLOS ONE 13(6): e0198356. 10.1371/journal.pone.0198356 PMC6010226 29924805

[B23] Parapar J Hutchings P (2014) Redescription of *Terebellides stroemii* (Polychaeta, Trichobranchidae) and designation of a neotype. Journal of the Marine Biological Association of the United Kingdom 95: 323–337. 10.1017/S0025315414000903

[B24] Parapar J Martin D Moreira J (2020) On the diversity of *Terebellides* (Annelida, Trichobranchidae) in West Africa, seven new species and the redescription of *T. africana* Augener, 1918 stat. prom. Zootaxa 4771(1): 1–61. 10.11646/zootaxa.4771.1.1 33055633

[B25] Parapar J Mikac B Fiege D (2013) Diversity of the genus *Terebellides* (Polychaeta: Trichobranchidae) in the Adriatic Sea with the description of a new species. Zootaxa 3691(3): 333–350. 10.11646/zootaxa.3691.3.3 26167589

[B26] Parapar J Moreira J (2008a) Redescription of *Terebellides kerguelensis* stat. nov. McIntosh, 1885 (Polychaeta: Trichobranchidae) from Antarctic and subantarctic waters. Helgoland Marine Research 62: 143–152. 10.1007/s10152-007-0085-4

[B27] Parapar J Moreira J (2008b) Revision of three species of *Terebellides* (Polychaeta: Trichobranchidae) described by C. Hessle in 1917 from the Southern Ocean. Journal of Natural History 42: 1261–1275. 10.1080/00222930801989997

[B28] Parapar J Moreira J Gil J Martin D (2016a) A new species of the genus *Terebellides* (Polychaeta, Trichobranchidae) from the Iranian coast. Zootaxa 4117(3): 321–340. 10.11646/zootaxa.4117.3.2 27395177

[B29] Parapar J Moreira J Helgason GV (2011) Taxonomy and distribution of *Terebellides* (Polychaeta, Trichobranchidae) in Icelandic waters, with the description of a new species. Zootaxa 2983: 1–20. 10.11646/zootaxa.2983.1.1

[B30] Parapar J Moreira J Martin D (2016b) On the diversity of the SE Indo-Pacific species of *Terebellides* (Annelida; Trichobranchidae), with the description of a new species. PeerJ 4: e2313. 10.7717/peerj.2313 PMC4991861 27602280

[B31] Parapar J Moreira J O’Reilly M (2016c) A new species of *Terebellides* (Polychaeta: Trichobranchidae) from Scottish waters with an insight into branchial morphology. Marine Biodiversity 46: 211–225. 10.1007/s12526-015-0353-5

[B32] Rach J DeSalle R Sarkar IN Schierwater B Hadrys H (2008) Character-based DNA barcoding allows discrimination of genera, species and populations in Odonata. Proceedings of the Royal Society B – Biological Sciences 275(1632): 237–247. 10.1098/rspb.2007.1290 PMC2212734 17999953

[B33] Read G Fauchald K (2020) World Polychaeta database. *Terebellides* Sars, 1835. [Accessed through: World Register of Marine Species at] http://www.marinespecies.org/aphia.php?p=taxdetails&id=129717 [accessed on 23 Sept 2020]

[B34] Sars M (1835) Beskrivelser og iagttagelser over nogle maerkelige eller nye i Havet ved den Bergenske Kyst levende Dyr af Polypernes, Acephalernes, Radiaternes, Annelidernes og Molluskernes Classer, med en kort Oversigt over de hidtil af Forfatteren sammesteds fundne Arter og deres Forekommen. Thorstein Hallagers Forlag hos Chr. Dahl, Bergen, 81 pp 10.5962/bhl.title.13017

[B35] Schüller M Hutchings PA (2010) New insights in the taxonomy of Trichobranchidae (Polychaeta) with the description of a new *Terebellides* from Australia. Zootaxa 2395: 1–16. 10.11646/zootaxa.2395.1.1

[B36] Schüller M Hutchings PA (2012) New species of *Terebellides* (Polychaeta: Trichobranchidae) indicate long-distance dispersal between western South Atlantic deep-sea basins. Zootaxa 3254: 1–31. 10.11646/zootaxa.3254.1.1 26131462

[B37] Schüller M Hutchings PA (2013) New species of *Terebellides* (Polychaeta: Trichobranchidae) from deep Southern Ocean. Zootaxa 3619: 1–45. 10.11646/zootaxa.3619.1.1 26131462

[B38] Tomioka S Kondoh T Sato-Okoshi W Ito K Kakui K Kajihara H (2016) Cosmopolitan or cryptic species? A case study of *Capitella teleta* (Annelida: Capitellidae). Zoological Science 33(5): 545–554. 10.2108/zs160059 27715419

[B39] Wong EHK Shivji MS Hanner RH (2009) Identifying sharks with DNA barcodes: assessing the utility of a nucleotide diagnostic approach. Molecular Ecology Resources 9: 243–256. 10.1111/j.1755-0998.2009.02653.x 21564984

[B40] Zhang J Hutchings P (2018) Taxonomy and distribution of *Terebellides* (Polychaeta: Trichobranchidae) in the northern South China Sea, with description of three new species. Zootaxa 4377(3): 387–411. 10.11646/zootaxa.4377.3.4 29690048

