# Supplementary material for: To name but a few: descriptions of five new species of Terebellides (Annelida, Trichobranchidae) from the North East Atlantic
Source: Zookeys. 2020 Nov 12;992:1–58. doi: 10.3897/zookeys.992.55977 (PMC7677295; doi:10.3897/zookeys.992.55977)
Supplement: Supplementary material 1 — Table S1. Locality and collecting data, museum registration numbers and references to figures of Terebellides specimens [file zookeys-992-001-s001.doc]

Supplementary Material - Table S1

| **Species 6 - *Terebellides europaea* Lavesque et al., 2019** | | | | | | | | | | | | | | | | |
| --- | --- | --- | --- | --- | --- | --- | --- | --- | --- | --- | --- | --- | --- | --- | --- | --- |
| **Specimen voucher** | **Site** | | **Geographic area** | **Locality** | | **Latitude** | | **Longitude** | **Depth (m)** | **Collecting date** | | **Habitat** | **Remarks** | | | **Figures** |
| GNM14625_d | KA6 | | Kattegat | Fladen | | 57.19717 | | 11.82517 | 38 | 17/06/2005 | | Silt, sand |  | | | 12E–F |
| GNM14625_2 | “ | | “ | “ | | “ | | “ | “ | “ | | “ |  | | |  |
| GNM14625_3 | “ | | “ | “ | | “ | | “ | “ | “ | | “ |  | | |  |
| GNM14628 | SK6 | | Skagerrak | Bonden | | 58.21947 | | 11.38658 | 8–18 | 26/04/2006 | | Mud, shells |  | | | 1D, 9B, 12–B |
| GNM15107 |  | |  |  | |  | |  |  |  | |  |  | | |  |
| GNM15114 | SK2 | | Skagerrak | W Kungälv | | 57.81822 | | 11.40038 | 39–67 | 09/06/2008 | | Shell, gravel |  | | |  |
| GNM15115 | SK1 | | “ | “ | | 57.80798 | | 11.56585 | 20–28 | “ | | “ |  | | |  |
| GNM15116 | SK22 | | “ | W Tanum | | 58.73875 | | 10.73752 | 102–173 | 15/06/2008 | | Clay, mud |  | | |  |
| GNM15120 | SK20 | | “ | E Väderöarna | | 58.58353 | | 11.08332 | 55–121 | “ | | Mixed bottom |  | | |  |
| GNM15121 | “ | | “ | “ | | “ | | “ | “ | “ | | “ |  | | |  |
| GNM15122 | SK23 | | “ | W Tanum | | 58.7398 | | 10.73842 | 98–148 | “ | | “ |  | | |  |
| GNM15123 | “ | | “ | “ | | “ | | “ | “ | “ | | “ |  | | |  |
| GNM15124 | “ | | “ | “ | | “ | | “ | “ | “ | | “ |  | | |  |
| GNM15125 | “ | | “ | “ | | “ | | “ | “ | “ | | “ |  | | |  |
| GNM14526 | “ | | “ | “ | | “ | | “ | “ | “ | | “ |  | | |  |
| GNM15127 | “ | | “ | “ | | “ | | “ | “ | “ | | “ |  | | |  |
| GNM15128 | “ | | “ | “ | | “ | | “ | “ | “ | | “ |  | | |  |
| ZMBN116334 | SK24 | | “ | Koster Area | | 58.86667 | | 11.10000 | 60–80 | 04/2005 | | Mud |  | | |  |
| ZMBN116335 | “ | | “ | “ | | “ | | “ | “ | “ | | “ |  | | |  |
| ZMBN116336 | ISCS4 | | Irish Sea, Celtic Sea | S Isle of Man | | 53.73567 | | -04.83767 | 54 | 07/2010 | | Sand, gravel |  | | |  |
| ZMBN116337 | ISCS1 | | “ | “ | | 53.60867 | | -04.38783 | 50 | 07/2010 | | “ |  | | |  |
| ZMBN116338 | ISCS2 | | “ | “ | | 53.62600 | | -04.46967 | 43 | “ | | “ |  | | |  |
| ZMBN116339 | “ | | “ | “ | | “ | | “ | “ | “ | | “ |  | | |  |
| ZMBN116340 | ISCS5 | | “ | “ | | 53.9520 | | -04.27867 | 42 | 07/2010 | | Gravel |  | | |  |
| ZMBN116341 | ISCS3 | | “ | “ | | 53.72067 | | -04.28283 | 46 | 07/2010 | | Sand, gravel |  | | |  |
| ZMBN116342 | “ | | “ | “ | | “ | | “ | “ | “ | | “ |  | | | 12C–D |
| ZMBN116343 | SK16 | | Skagerrak | Aust-Agder, Utnes | | 58.41023 | | 08.74602 | 22–23 | 25/06/2011 | | Algae, ascidians |  | | |  |
| ZMBN116344 | “ | | “ | “ | | “ | | “ | “ | “ | | “ |  | | |  |
| ZMBN116346 | SK24 | | “ | Koster Area | | 58.86667 | | 11.10000 | 60–80 | 04/2005 | | Mud |  | | |  |
| ZMBN116347 | NCS13 | | Norwegian coast, shelf | Hordaland, Toskasundet | | 60.65862 | | 04.94718 | 13 | 04/06/2014 | | -- |  | | |  |
| **Species 7 - *Terebellides ronningae* sp. nov.** | | | | | | | | | | | | | | | | |
| **Specimen voucher** | **Site** | | **Geographic area** | **Locality** | | **Latitude** | | **Longitude** | **Depth (m)** | **Collecting date** | | **Habitat** | **Remarks** | | | **Figures** |
| ZMBN 116348 | SK25 | | Skagerrak | SW Yttre Vattenholmen | | 58.87417 | | 11.09472 | 62–71 | 08/04/2008 | | Mud |  | | |  |
| ZMBN 116349 | “ | | “ | “ | | “ | | “ | “ | “ | | “ | SEM | | | 19A, D, 20A–D |
| ZMBN 116350 | NCS2 | | Norwegian coast, shelf | Rogaland, S Kitsøy | | 59.02985 | | 05.44881 | 58–60 | 10/06/2014 | | Stones, gravel, sand |  | | |  |
| ZMBN 116352 | “ | | “ | “ | | “ | | “ | “ | “ | | “ |  | | |  |
| ZMBN 116353 | “ | | “ | “ | | “ | | “ | “ | “ | | “ | SEM | | | 19B–C, 20E–F, 26E |
| ZMBN 116354 | “ | | “ | “ | | “ | | “ | “ | “ | | “ |  | | |  |
| ZMBN 116355 | “ | | “ | “ | | “ | | “ | “ | “ | | “ |  | | |  |
| ZMBN 116356 | “ | | “ | “ | | “ | | “ | “ | “ | | “ |  | | |  |
| ZMBN 116357 | NCS7 | | “ | Hordaland. Lysefjord | | 60.21465 | | 05.3472 | 25–47 | 28/06/2007 | | n.d. | **HOLOTYPE** | | | 17A, 18A–B |
| ZMBN 116358 | “ | | “ | “ | | “ | | “ | “ | “ | | “ |  | | |  |
| ZMBN 116359 | NCS21 | | “ | Sogn Fjordane-Møre & Romsdal | | 62.27842 | | 05.45413 | 169–188 | 21/07/2012 | | “ |  | | |  |
| **Species 8 - *Terebellides norvegica* sp. nov.** | | | | | | | | | | | | | | | | |
| **Specimen voucher** | **Site** | | **Geographic area** | **Locality** | | **Latitude** | | **Longitude** | **Depth (m)** | **Collecting date** | | **Habitat** | **Remarks** | | | **Figures** |
| GNM146323 |  | |  |  | |  | |  |  |  | |  |  | | |  |
| GNM14637 | SK19 | | Skagerrak | Aust-Agder, Utnes | | 58.48285 | | 10.13443 | 491–531 | 06/06/2016 | | Soft bottom |  | | |  |
| GNM15130 | SK7 | | “ |  | | 58.2237 | | 09.9267 | 453–477 | 13/05/2009 | | Mud |  | | | 21B |
| GNM15131 | “ | | “ |  | | “ | | “ | “ |  | | “ |  | | |  |
| GNM15132 | “ | | “ |  | | “ | | “ | “ | “ | | “ |  | | |  |
| GNM15134 | “ | | “ |  | | “ | | “ | “ | “ | | “ |  | | | 21A, C–D, 22 |
| NTNU-VM61388 | NCS24 | | Norwegian coast. shelf | Sør-Trøndelang, Trondheimfjord | | 63.47672 | | 09.92872 | 534 | 17/01/2013 | | Mud |  | | |  |
| NTNU-VM61389 | “ | | “ | “ | | “ | | “ | “ | “ | | “ |  | | |  |
| NTNU-VM61390 | NCS25 | | “ | “ | | 63.47903 | | 10.21283 | 502–505 | “ | | “ |  | | |  |
| NTNU-VM66569 | NCS29 | | “ | Sør-Trøndelang, Frohavet | | 63.75767 | | 09.20882 | 350–357 | 10/05/2010 | | “ |  | | |  |
| NTNU-VM66573 | “ | | “ | “ | | “ | | “ | “ | “ | | “ |  | | |  |
| NTNU-VM66574 | “ | | “ | “ | | “ | | “ | “ | “ | | “ |  | | |  |
| NTNU-VM68197 | NCS24 | | “ | Sør-Trøndelang, Trondheimfjord | | 63.47672 | | 09.92872 | 534 | 17/01/2013 | | “ |  | | |  |
| NTNU-VM68198 | “ | | “ | “ | | “ | | “ | “ | “ | | “ |  | | |  |
| ZMBN116361 | SK13 | | Skagerrak | Aust-Agder, Ryvingdypet | | 58.36978 | | 08.72617 | 190 | 28/05/2011 | | “ |  | | |  |
| ZMBN116362 | NCS38 | | Norwegian coast. shelf | Nordland, Hellemofjord | | 67.87383 | | 16.35300 | 466 | 04/03/2008 | | n.d. |  | | |  |
| ZMBN116363 | NCS37 | | “ | “ | | 67.86733 | | 16.37033 | 461 | “ | | “ |  | | |  |
| ZMBN116364 | BS2 | | Barents Sea | Troms, Ullsfjorden, S Karlsøya | | 69.95333 | | 20.07183 | 243 | 07/122009 | | “ |  | | |  |
| ZMBN116365 | “ | | “ | “ | | “ | | “ | “ | “ | | “ |  | | |  |
| ZMBN116366 | BS3 | | “ | Finnmark, Altafjord | | 70.1165 | | 23.07533 | 392 | 09/12/2009 | | “ |  | | |  |
| ZMBN116367 | “ | | “ | “ | | “ | | “ | “ | “ | | “ |  | | |  |
| ZMBN116368 | NCS36 | | Norwegian coast, shelf | Nordland, Skjaersadfjord | | 67.26417 | | 14.86983 | 513 | 13/10/2010 | | “ |  | | |  |
| ZMBN116369 | NCS35 | | “ | “ | | 67.21783 | | 15.27833 | 476 | 14/10/2010 | | “ |  | | |  |
| ZMBN116370 | “ | | “ | “ | | “ | | “ | “ | “ | | “ |  | | |  |
| ZMBN116371 | NCS28 | | “ | Sør-Trøndelang, Trondheimfjord | | 63.73615 | | 10.97631 | 419 | 27/05/2012 | | “ |  | | |  |
| ZMBN116372 | “ | | “ | “ | | “ | | “ | “ | “ | | “ |  | | |  |
| ZMBN116373 | NCS27 | | “ | Sør-Trøndelang, Trondheimfjord | | 63.71208 | | 10.89915 | 420 | 27/05/2012 | | “ |  | | |  |
| ZMBN116374 | NCS45 | | “ | Nordland, Gullesfjord | | 68.71076 | | 16.01100 | 209 | 06/11/2008 | | “ |  | | |  |
| ZMBN116375 | “ | | “ | “ | | “ | | “ | “ | “ | | “ |  | | |  |
| ZMBN116376 | “ | | “ | “ | | “ | | “ | “ | “ | | “ |  | | |  |
| ZMBN116377 | NCS3 | | “ | Rogaland | | 59.20548 | | 05.78051 | 226–242 | 11/06/2014 | | “ |  | | |  |
| ZMBN116378 | NCS3 | | “ | “ | | “ | | “ | “ | “ | | “ | **HOLOTYPE** | | | 17B, 18C–D |
| ZMBN116379 | NCS20 | | “ | Sogn & Fjordane-More & Romsdal | | 61.82371 | | 05.21031 | 446–453 | 20/07/2012 | | “ |  | | |  |
| ZMBN116380 | NCS19 | | “ | “ | | 61.80178 | | 05.08135 | 370–375 | 20/07/2012 | | “ |  | | |  |
| ZMBN116381 | “ | | “ | “ | | “ | | “ | “ | “ | | “ |  | | |  |
| ZMBN116382 | NCS17 | | “ | Sogn & Fjordane, Sognefjorden | | 61.14484 | | 05.91575 | 1259–1268 | 16/11/2012 | | “ |  | | |  |
| ZMBN116383 | NCS20 | | “ | Sogn & Fjordane-More & Romsdal | | 61.82371 | | 05.21031 | 446–453 | 20/07/2012 | | “ |  | | |  |
| ZMBN116384 | NCS5 | | “ | Hordaland, Langeneuen | | 59.99000 | | 05.35000 | 250 | 26/06/2007 | | “ |  | | |  |
| **Species 9 - *Terebellides scotica* sp. nov.** | | | | | | | | | | | | | | | | |
| **Specimen voucher** | **Site** | | **Geographic area** | **Locality** | | **Latitude** | | **Longitude** | **Depth (m)** | **Collecting date** | | **Habitat** | **Remarks** | | | **Figures** |
| ZMBN 116382 | NS6 | | North Sea | S Shetland Islands | | 60.17983 | | -01.38883 | 48 | 07/2008 | | Sandy clay, gravel |  | | | 23–24 |
| ZMBN 116385 | NS3 | | “ | E Orkney Island | | 58.87367 | | -02.19000 | 85 | “ | | “ | **HOLOTYPE** | | | 17C, 18E–F |
| ZMBN 116386 | NS5 | | “ | W Shetland Islands | | 60.0675 | | -01.54467 | 111 | “ | | “ |  | | |  |
| ZMBN 116387 | NS6 | | “ | S Shetland Islands | | 60.17983 | | -01.38883 | 48 | “ | | “ |  | | |  |
| SMA_BR_23 | - | | Gulf of Biscay | Bay of Brest | | 48.31527 | | -04.36470 | 50 | 05/2018 | | maërl | In Lavesque et al. (2019) as *Terebellides* sp. | | |  |
| SMA_BR_33 | - | | “ | “ | | “ | | “ | “ | “ | | “ | “ | | |  |
| **Species 10 - *Terebellides bakkeni* sp. nov.** | | | | | | | | | | | | | | | | |
| **Specimen voucher** | **Site** | | **Geographic area** | **Locality** | | **Latitude** | | **Longitude** | **Depth (m)** | **Collecting date** | | **Habitat** | **Remarks** | | | **Figures** |
| ZMBN 116388 | BS7 | | Barents Sea | Finmark | | 70.77383 | | 30.78117 | 377–378 | 17/08/2103 | | Mud |  | | |  |
| ZMBN 116389 | BS2 | | “ | Troms, Ullsfjorden, S Karlsøya | | 69.95333 | | 20.07183 | 243 | 07/12/2009 | | n.d. |  | | |  |
| ZMBN 116390 | NCS41 | | Norwegian coast, shelf | Nordland, Sortlaandssunder | | 68.62817 | | 15.34959 | 128 | 07/11/2007 | | “ |  | | |  |
| ZMBN 116391 | NCS42 | | “ | “ | | 68.62856 | | 15.35318 | 122 | “ | | “ |  | | |  |
| ZMBN 116392 | NCS46 | | “ | “ | | 68.79015 | | 15.41222 | 108 | 08/11/2008 | | “ |  | | |  |
| ZMBN 1163893 | NCS47 | | “ | “ | | 68.79963 | | 15.41033 | 119 | “ | | “ |  | | |  |
| ZMBN 116394 | NCS47 | | “ | “ | | “ | | “ | “ | “ | | “ |  | | |  |
| ZMBN 116395 | NCS47 | | “ | “ | | “ | | “ | “ | “ | | “ | **HOLOTYPE** | | | 1A, 2A–B |
| ZMBN 116396 | NCS47 | | “ | “ | | “ | | “ | “ | “ | | “ |  | | |  |
| NTNU-VM 61376 | NCS30 | | “ | Sør-Trøndelag, Åfjord | | 63.99012 | | 10.04445 | 102–110 | 11/07/2007 | | “ |  | | | 3C, E, 4B, G, 26A |
| NTNU-VM 61377 | NCS30 | | “ | “ | | “ | | “ | “ | “ | | “ |  | | | 3A–B, D, F, 4C–F |
| **Species 11 - *Terebellides stroemii* Sars, 1835** | | | | | | | | | | | | | | | | |
| **Specimen voucher** | | **Site** | **Geographic area** | | **Locality** | | **Latitude** | **Longitude** | **Depth (m)** | **Collecting date** | **Habitat** | | | **Remarks** | **Figures** | |
| ZMBN 116397 | | NCS26 | Norwegian coast, shelf | | Sør-Trondelag, Trondheimfjord | | 63.48733 | 10.37383 | 271–334 | 15/01/2002 | Mud | | | Drawing | 1B, 2C | |
| ZMBN 116398 | | NCS31 | “ | | Storegga | | 64.19888 | 06.06968 | 387–388 | 26/06/2013 | Muddy sand | | |  |  | |
| ZMBN 116399 | | NCS33 | “ | | Skjolddryggen | | 65.50056 | 06.26848 | 397–420 | 23/06/2013 | Sandy mud | | |  | 5, 26B | |
| ZMBN 11400 | | NCS14 | “ | | Sogn & Fjordane, Aurlandsfjord | | 60.90389 | 07.16813 | 115 | 17/11/2012 | n.d. | | |  |  | |
| ZMBN 11401 | | NCS12 | “ | | Hordaland, Mangerfjord | | 60.62360 | 04.94120 | 325 | 07/02/2006 | “ | | |  |  | |
| NHMO C5896 | | - | Bergenfjord | | Helle, Manger | | 60.62083 | 05.03333 | 55–110 | - | sand | | | Neotype |  | |
| NHMO C5899 | | - | “ | | “ | | “ | “ | “ | - | “ | | | Neoparatype |  | |
| NHMO C5902 | | - | “ | | “ | | “ | “ | “ | - | “ | | | “ |  | |
| NHMO C5904 | | - | “ | | “ | | “ | “ | “ | - | “ | | | “ |  | |
| NHMO C5905 | | - | “ | | “ | | “ | “ | “ | - | “ | | | “ |  | |
| NHMO C5907 | | - | “ | | “ | | “ | “ | “ | - | “ | | | “ |  | |
| NHMO C5956 | | - | “ | | “ | | “ | “ | “ | - | “ | | | “ |  | |
| NHMO C5968 | | - | “ | | “ | | “ | “ | “ | - | “ | | | “ |  | |
| **Species 12 - *Terebellides* sp. 1** | | | | | | | | | | | | | | | | |
| **Specimen voucher** | | **Site** | **Geographic area** | | **Locality** | | **Latitude** | **Longitude** | **Depth (m)** | **Collecting date** | **Habitat** | | | **Remarks** | **Figures** | |
| GNM 14630-4 | | SK 9 | Skagerrak | |  | | 58.29293 | 11.51555 | 44–101 | 27/04/2006 | Mixed bottom | | |  | 13 | |
| GNM 14630-8 | | SK 9 | “ | |  | | “ | “ | “ | “ | “ | | |  | 13 | |
| **Species 13 - *Terebellides kongsrudi* sp. nov.** | | | | | | | | | | | | | | | | |
| **Specimen voucher** | | **Site** | **Geographic area** | | **Locality** | | **Latitude** | **Longitude** | **Depth (m)** | **Collecting date** | **Habitat** | | | **Remarks** | **Figures** | |
| GNM15136 | | SK7 | Skagerrak | |  | | 58.2237 | 09.9267 | 453–477 | 13/05/2009 | Mud | | |  |  | |
| GNM14632 | | SK12 | “ | |  | | 58.36037 | 10.24012 | 429–445 | 29/05/2006 | Soft bottom | | | **HOLOTYPE** | 1C, 2D–E | |
| GNM14638 | | SK14 | “ | |  | | 58.40322 | 10.51548 | 273–365 | 21/08/2006 | Mixed bottom | | |  |  | |
| ZMBN 116409 | | BS8 | Barents Sea | | Finnmark | | 71.05600 | 29.65567 | 337 | 21/04/2014 | Muddy sand | | |  | 9A–B, F, 10, 26C | |
| ZMBN 116411 | | BS11 | “ | | Finnmark, TOO | | 71.61527 | 32.99719 | 305–306 | 09/08/2013 | Mud, clay | | |  | 9C–E | |
| ZMBN 116412 | | NCS3 | Norwegian coast, shelf | | Rogaland | | 59.20548 | 05.78051 | 226–242 | 11/06/2014 | n.d. | | |  |  | |
| ZMBN 116413 | | NCS3 | “ | | “ | | “ | “ | “ | “ | “ | | |  |  | |
| ZMBN 116414 | | BS6 | Barents Sea | | Finnmark, Porsangerfjord | | 70.35324 | 25.26369 | 178 | 20/05/2009 | “ | | |  |  | |
| ZMBN 116415 | | NCS34 | Norwegian coast, shelf | | Nordland, Holmsund | | 67.03925 | 13.85357 | 259 | 13/05/2012 | “ | | |  |  | |
| ZMBN 116416 | | NCS22 | “ | | Møre & Romsdal, Harøyfjord | | 62.71988 | 06.58989 | 126 | 20/05/2012 | “ | | |  |  | |
| ZMBN 116417 | | NCS46 | “ | | Nordland, Sortlandssundet | | 68.79015 | 15.41222 | 108 | 08/11/2008 | “ | | |  |  | |
| ZMBN 116418 | | NCS46 | “ | | “ | | “ | “ | “ | “ | “ | | |  |  | |
| NTNU-VM-66568 | | NCS29 | “ | | Sør-Trondelag, Frohavet | | 63.75767 | 09.20882 | 350–357 | 10/05/2010 | Mud | | |  |  | |
| NTNU-VM-66570 | | NCS29 | “ | | “ | | “ | “ | “ | “ | “ | | |  |  | |
| NTNU-VM-66571 | | NCS29 | “ | | “ | | “ | “ | “ | “ | “ | | |  |  | |
| NTNU-VM-66572 | | NCS29 | “ | | “ | | “ | “ | “ | “ | “ | | |  |  | |
| NTNU-VM-68195 | | NCS25 | “ | | Sør-Trondelag, Trondheimfjord | | 63.47903 | 10.21283 | 502–505 | 17/01/2013 | “ | | |  |  | |
| NTNU-VM-72560 | | NCS39 | “ | | “ | | 68.47672 | 09.92872 | 534 | “ | “ | | |  |  | |
| NTNU-VM-72561 | | NCS25 | “ | | “ | | 63.47903 | 10.21283 | 502–505 | “ | “ | | |  |  | |
| NTNU-VM-72562 | | NCS24 | “ | | “ | | 63.47672 | 09.92872 | 534 | “ | “ | | |  |  | |
| NTNU-VM-72563 | | NCS25 | “ | | “ | | 63.47903 | 10.21283 | 502–505 | “ | “ | | |  |  | |
| **Species 21 - *Terebellides* sp. 2** | | | | | | | | | | | | | | | | |
| **Specimen voucher** | | **Site** | **Geographic area** | | **Locality** | | **Latitude** | **Longitude** | **Depth (m)** | **Collecting date** | **Habitat** | | | **Remarks** | **Figures** | |
| ZMBN 116481 | | BS10 | Barents Sea | | Finnmark, TOO | | 71.61416 | 33.0041 | 305 | 09/08/2013 | Mud, clay | | |  | 27 | |
| ZMBN 116486 | | BS16 | “ | | Svalbard | | 80.10100 | 22.20060 | 161 | 01/09/2009 | - | | |  | 27 | |
| **Species 20+28 - *Terebellides bigeniculatus* Parapar, Helgason & Moreira, 2011** | | | | | | | | | | | | | | | | |
| **Specimen voucher** | | **Site** | **Geographic area** | | **Locality** | | **Latitude** | **Longitude** | **Depth (m)** | **Collecting date** | **Habitat** | | | **Remarks** | **Figures** | |
| ZMBN 116477 | | NCS33 | Norwegian coast, shelf | | Skjoldryggen | | 65.50056 | 06.26848 | 397–420 | 26/03/2013 | Sandy mud | | | clade 20 |  | |
| ZMBN 116510 | | NCS31 | “ | | Storegga | | 64.19888 | 06.06965 | 387–388 | 26/06/2013 | Muddy sand | | | clade 28 |  | |
| ZMBN 116511 | | BS14 | Barents Sea | | Finnmark, TOO | | 72.57905 | 32.38726 | 271–272 | 03/08/2013 | Sandy mud | | | “ |  | |
| ZMBN 116512 | | NCS33 | Norwegian coast, shelf | | Skjoldryggen | | 65.50056 | 06.26848 | 397–420 | 26/03/2013 | “ | | | “ | 25C–D | |
| ZMBN 116513 | | NCS18 | “ | | Sogn & Fjordane, Lustra-Nattropefjorden | | 61.43212 | 07.47763 | 327–337 | 18/11/2012 | n.d. | | | “ | 25A–B, E–F | |
| ZMBN 116514 | | NCS11 | “ | | Hordaland, Herdlanfjord | | 60.51018 | 05.19228 | 375 | 20/04/2007 | “ | | | clade 28 | 2F, 17D | |
| IINH 24923 | | - | North Iceland | | - | | 67.21472 | -22.42944 | 333 | 16/07/1993 | Organic sediment | | | **Holotype** |  | |
| IINH 24925 | | - | East Iceland | | - | | 65.70250 | -12.88333 | 272 | 25/07/1991 | - | | | 5 Paratypes |  | |
