# Supplementary material for: To name but a few: descriptions of five new species of Terebellides (Annelida, Trichobranchidae) from the North East Atlantic
Source: Zookeys. 2020 Nov 12;992:1–58. doi: 10.3897/zookeys.992.55977 (PMC7677295; doi:10.3897/zookeys.992.55977)
Supplement: Supplementary material 2 — Table S2. List of COI sequences considered in present study (Group A), museum vouchers and GenBank accession numbers [file zookeys-992-001-s002.docx]

Supplementary Material – Table S2

List of COI sequences considered in present study (Group A), museum vouchers and GenBank accession numbers.

| **Type material** | **Specimen voucher** | **Sequence ID** | **GenBank Acc. #** |  |
| --- | --- | --- | --- | --- |
| ***Terebellides europaea*** | | | | |
| Non-type | GNM 15114 | 838_6 | MG025072 |  |
| Non-type | GNM 15115 | 839_6 | MG025073 |  |
| Non-type | GNM 15116 | 845_6 | MG025074 |  |
| Non-type | GNM 15117 | 846_6 | MG025075 |  |
| Non-type | GNM 15118 | 847_6 | MG025076 |  |
| Non-type | GNM 15119 | 848_6 | MG025077 |  |
| Non-type | GNM 15120 | 849_6 | MG025078 |  |
| Non-type | GNM 15121 | 850_6 | MG025079 |  |
| Non-type | ZMBN 116333 | 860_6 | MG025080 |  |
| Non-type | GNM 15122 | 1313_6 | MG025081 |  |
| Non-type | GNM 15123 | 1314_6 | MG025082 |  |
| Non-type | GNM 15124 | 1315_6 | MG025083 |  |
| Non-type | GNM 15125 | 1316_6 | MG025084 |  |
| Non-type | GNM 15126 | 1317_6 | MG025085 |  |
| Non-type | GNM 15127 | 1318_6 | MG025086 |  |
| Non-type | GNM 15128 | 1319_6 | MG025087 |  |
| Non-type | ZMBN 116335 | 1321_6 | MG025088 |  |
| Non-type | ZMBN 116336 | 1869_6 | MG025089 |  |
| Non-type | ZMBN 116337 | 1870_6 | MG025090 |  |
| Non-type | ZMBN 116338 | 1871_6 | MG025091 |  |
| Non-type | ZMBN 116339 | 1872_6 | MG025092 |  |
| Non-type | ZMBN 116340 | 1873_6 | MG025093 |  |
| Non-type | ZMBN 116341 | 1874_6 | MG025094 |  |
| Non-type | ZMBN 116342 | 1875_6 | MG025095 |  |
| Non-type | ZMBN 116343 | 1942_6 | MG025096 |  |
| Non-type | ZMBN 116344 | 1943_6 | MG025097 |  |
| Non-type | ZMBN 116345 | 1944_6 | MG025098 |  |
| Non-type | ZMBN 116346 | 2046_6 | MG025099 |  |
| Non-type | GNM 14625_d | 2167_6 | MG025100 |  |
| Non-type | GNM 14628_1 | 2172_6 | MG025101 |  |
| Non-type | GNM 14628 | 2173_6 | MG025102 |  |
| Non-type | GNM 14625 | 2190_6 | MG025103 |  |
| Non-type | ZMBN 116347 | 2774_6 | MG025104 |  |
| ***Terebellides ronningae* sp. nov**. | | | | |
| Paratype | ZMBN 116348 | 829_7 | MG025105 |  |
| Paratype | ZMBN 116349 | 1309_7 | MG025106 |  |
| Paratype | ZMBN 116350 | 2442_7 | MG025107 |  |
| Non-type | ZMBN 116351 | 2443_7 | MG025108 |  |
| Paratype | ZMBN 116352 | 2447_7 | MG025109 |  |
| Paratype | ZMBN 116353 | 2448_7 | MG025110 |  |
| Paratype | ZMBN 116354 | 2449_7 | MG025111 |  |
| Paratype | ZMBN 116355 | 2450_7 | MG025112 |  |
| Paratype | ZMBN 116356 | 2452_7 | MG025113 |  |
| Holotype | ZMBN 116357 | 2859_7 | MG025114 |  |
| Paratype | ZMBN 116358 | 2863_7 | MG025115 |  |
| Paratype | ZMBN 116359 | 2914_7 | MG025116 |  |
| ***Terebellides norvegica* sp. nov.** | | | | |
| Non-type | GNM 15129 | 1197_8 | MG025117 |  |
| Non-type | GNM 15130 | 1198_8 | MG025118 |  |
| Paratype | GNM 15131 | 1199_8 | MG025119 |  |
| Paratype | GNM 15132 | 1200_8 | MG025120 |  |
| Non-type | GNM 15133 | 1202_8 | MG025121 |  |
| Paratype | GNM 15134 | 1203_8 | MG025122 |  |
| Non-type | ZMBN 116360 | 1561_8 | MG025123 |  |
| Paratype | NTNU-VM-66569 | 1922_8 | MG025124 |  |
| Non-type | ZMBN 116361 | 1946_8 | MG025125 |  |
| Paratype | NTNU-VM-66574 | 1957_8 | MG025126 |  |
| Paratype | NTNU-VM-66573 | 1958_8 | MG025127 |  |
| Paratype | ZMBN 116362 | 1984_8 | MG025128 |  |
| Paratype | ZMBN 116363 | 1985_8 | MG025129 |  |
| Non-type | ZMBN 116364 | 1988_8 | MG025130 |  |
| Paratype | ZMBN 116365 | 1989_8 | MG025131 |  |
| Paratype | ZMBN 116366 | 1991_8 | MG025132 |  |
| Non-type | ZMBN 116367 | 1992_8 | MG025133 |  |
| Paratype | ZMBN 116368 | 1994_8 | MG025134 |  |
| Paratype | ZMBN 116369 | 1995_8 | MG025135 |  |
| Paratype | ZMBN 116370 | 1996_8 | MG025136 |  |
| Paratype | ZMBN 116371 | 2000_8 | MG025137 |  |
| Paratype | ZMBN 116372 | 2001_8 | MG025138 |  |
| Paratype | ZMBN 116373 | 2002_8 | MG025139 |  |
| Paratype | ZMBN 116374 | 2013_8 | MG025140 |  |
| Paratype | ZMBN 116375 | 2014_8 | MG025141 |  |
| Paratype | ZMBN 116376 | 2015_8 | MG025142 |  |
| Paratype | NTNU-VM-61388 | 2036_8 | MG025143 |  |
| Paratype | NTNU-VM-61389 | 2037_8 | MG025144 |  |
| Paratype | NTNU-VM-61390 | 2039_8 | MG025145 |  |
| Paratype | GNM 14637 | 2214_8 | MG025146 |  |
| Paratype | ZMBN 116377 | 2456_8 | MG025147 |  |
| Holotype | ZMBN 116378 | 2457_8 | MG025148 |  |
| Paratype | NTNU-VM-68197 | 2476_8 | MG025149 |  |
| Paratype | NTNU-VM-68198 | 2478_8 | MG025150 |  |
| Paratype | ZMBN 116379 | 2775_8 | MG025151 |  |
| Paratype | ZMBN 116380 | 2798_8 | MG025152 |  |
| Paratype | ZMBN 116381 | 2799_8 | MG025153 |  |
| Paratype | ZMBN 116382 | 2896_8 | MG025154 |  |
| Paratype | ZMBN 116383 | 2920_8 | MG025155 |  |
| Paratype | ZMBN 116384 | 2925_8 | MG025156 |  |
| ***Terebellides scotica* sp. nov.** | | | | |
| Holotype | ZMBN 116385 | 859_9 | MG025157 |  |
| Paratype | ZMBN 116387 | 862_9 | MG025158 |  |
| Non-type | SMA_BR_33 | SMA_BR_33 | MN207188 |  |
| ***Terebellides bakkeni* sp. nov.** | | | | |
| Paratype | ZMBN 116389 | 1990_10 | MG025159 |  |
| Paratype | ZMBN 116390 | 2024_10 | MG025160 |  |
| Paratype | ZMBN 116391 | 2026_10 | MG025161 |  |
| Paratype | ZMBN 116392 | 2029_10 | MG025162 |  |
| Paratype | ZMBN 116393 | 2031_10 | MG025163 |  |
| Paratype | ZMBN 116394 | 2032_10 | MG025164 |  |
| Holotype | ZMBN 116395 | 2033_10 | MG025165 |  |
| Paratype | ZMBN 116396 | 2034_10 | MG025166 |  |
| Non-type | SMF 24688 | 2304_10 | MG025167 |  |
| Paratype | ZMBN 116388 | 2321_10 | MG025168 |  |
| Paratype | NTNU-VM-61376 | TB25_10 | MG025169 |  |
| Paratype | NTNU-VM-61377 | TB26_10 | MG025170 |  |
| ***Terebellides stroemii*** | | | | |
| Non-type | ZMBN 116397 | 1560_11 | MG025171 |  |
| Non-type | ZMBN 116398 | 2323_11 | MG025172 |  |
| Non-type | ZMBN 116399 | 2347_11 | MG025173 |  |
| Non-type | ZMBN 116401 | 2786_11 | MG025174 |  |
| Non-type | ZMBN 116400 | 2899_11 | MG025175 |  |
| ***Terebellides* sp. 1** | | | | |
| Non-type | GNM 15135 | 1312_12 | MG025176 |  |
| Non-type | GNM 14644_1 | 2171_12 | MG025177 |  |
| Non-type | GNM 14630 | 2193_12 | MG025178 |  |
| Non-type | GNM 14630_1 | 2194_12 | MG025179 |  |
| Non-type | GNM 14630_2 | 2195_12 | MG025180 |  |
| Non-type | GNM 14630_3 | 2196_12 | MG025181 |  |
| Non-type | GNM 14630_4 | 2197_12 | MG025182 |  |
| Non-type | GNM 14630_5 | 2198_12 | MG025183 |  |
| Non-type | GNM 14630_6 | 2199_12 | MG025184 |  |
| Non-type | GNM 14630_7 | 2200_12 | MG025185 |  |
| Non-type | GNM 14630_8 | 2201_12 | MG025186 |  |
| Non-type | GNM 14631 | 2202_12 | MG025187 |  |
| Non-type | GNM 14644 | 2222_12 | MG025188 |  |
| Non-type | GNM 14644_2 | 2223_12 | MG025189 |  |
| Non-type | GNM 14644_3 | 2224_12 | MG025190 |  |
| Non-type | GNM 14644_4 | 2225_12 | MG025191 |  |
| Non-type | ZMBN 116404 | 2806_12 | MG025192 |  |
| Non-type | ZMBN 116408 | 2818_12 | MG025193 |  |
| Non-type | ZMBN 116406 | 2824_12 | MG025194 |  |
| Non-type | ZMBN 116407 | 2826_12 | MG025195 |  |
| Non-type | ZMBN 116402 | 2827_12 | MG025196 |  |
| Non-type | ZMBN 116405 | 2829_12 | MG025197 |  |
| Non-type | ZMBN 116403 | 2832_12 | MG025198 |  |
| ***Terebellides kongsrudi* sp. nov.** | | | | |
| Non-type | GNM 15136 | 1201_13 | MG025199 |  |
| Non-type | GNM 15137 | 1205_13 | MG025200 |  |
| Paratype | NTNU-VM-66572 | 1923_13 | MG025201 |  |
| Paratype | NTNU-VM-66571 | 1956_13 | MG025202 |  |
| Paratype | NTNU-VM-66568 | 1959_13 | MG025203 |  |
| Paratype | NTNU-VM-66570 | 1960_13 | MG025204 |  |
| Non-type | ZMBN 116414 | 1986_13 | MG025205 |  |
| Non-type | ZMBN 116415 | 1998_13 | MG025206 |  |
| Non-type | ZMBN 116416 | 1999_13 | MG025207 |  |
| Non-type | ZMBN 116417 | 2027_13 | MG025208 |  |
| Non-type | ZMBN 116418 | 2028_13 | MG025209 |  |
| Paratype | NTNU-VM-72560 | 2035_13 | MG025210 |  |
| Paratype | NTNU-VM-72561 | 2038_13 | MG025211 |  |
| Paratype | ZMBN 116409 | 2183_13 | MG025212 |  |
| Non-type | ZMBN 116410 | 2184_13 | MG025213 |  |
| Paratype | GNM 14638 | 2215_13 | MG025214 |  |
| Non-type | SMF 24659 | 2317_13 | MG025215 |  |
| Paratype | ZMBN 116411 | 2337_13 | MG025216 |  |
| Paratype | ZMBN 116412 | 2454_13 | MG025217 |  |
| Paratype | ZMBN 116413 | 2458_13 | MG025218 |  |
| Paratype | NTNU-VM-68195 | 2475_13 | MG025219 |  |
| Non-type | ZMBN 116419 | 2776_13 | MG025220 |  |
| Non-type | ZMBN 116420 | 2813_13 | MG025221 |  |
| Non-type | ZMBN 116421 | 2921_13 | MG025222 |  |
| Paratype | NTNU-VM-72562 | T01_13 | MG025223 |  |
| Paratype | NTNU-VM-72563 | T03_13 | MG025224 |  |
| ***Terebellides bigeniculatus*** | | | | |
| Non-type | SMF 24636 | 2302_20 | MG025318 |  |
| Non-type | ZMBN 116477 | 2349_20 | MG025319 |  |
| Non-type | ZMBN 116510 | 2324_28 | MG025351 |  |
| Non-type | ZMBN 116511 | 2329_28 | MG025352 |  |
| Non-type | ZMBN 116512 | 2348_28 | MG025353 |  |
| Non-type | ZMBN 116513 | 2875_28 | MG025354 |  |
| Non-type | ZMBN 116514 | 2903_28 | MG025355 |  |
| ***Terebellides* sp. 2** | | | | |
| Non-type | ZMBN 116478 | 2342_21 | MG025320 |  |
| Non-type | ZMBN 116479 | 2377_21 | MG025321 |  |
| Non-type | ZMBN 116480 | 2384_21 | MG025322 |  |
| Non-type | ZMBN 116481 | 2385_21 | MG025323 |  |
| Non-type | ZMBN 116484 | 2815_21 | MG025324 |  |
| Non-type | ZMBN 116482 | 2816_21 | MG025325 |  |
| Non-type | ZMBN 116483 | 2817_21 | MG025326 |  |
| Non-type | ZMBN 116493 | 2819_21 | MG025327 |  |
| Non-type | ZMBN 116494 | 2820_21 | MG025328 |  |
| Non-type | ZMBN 116485 | 2823_21 | MG025329 |  |
| Non-type | ZMBN 116486 | 2825_21 | MG025330 |  |
| Non-type | ZMBN 116495 | 2830_21 | MG025331 |  |
| Non-type | ZMBN 116487 | 2831_21 | MG025332 |  |
| Non-type | ZMBN 116488 | 2833_21 | MG025333 |  |
| Non-type | ZMBN 116489 | 2834_21 | MG025334 |  |
| Non-type | ZMBN 116490 | 2836_21 | MG025335 |  |
| Non-type | ZMBN 116491 | 2894_21 | MG025336 |  |
| Non-type | ZMBN 116492 | 2895_21 | MG025337 |  |
| ***Terebellides lilasae*** |  |  |  |  |
| Non-type | SMA_VOG8C2-A | SMA_VOG8C2-A | MN207186.1 |  |
| Non-type | SMA_VOG8C2-B | SMA_VOG8C2-B | MN207186.1 |  |
| Non-type | SMA_VOG8C2-C | SMA_VOG8C2-C | MN207186.1 |  |
| Non-type | SMA_BR_42 | SMA_BR_42 | MN207186.1 |  |
